# Time-resolved proteomics of adenovirus infected cells

**DOI:** 10.1371/journal.pone.0204522

**Published:** 2018-09-25

**Authors:** Alberto Valdés, Hongxing Zhao, Ulf Pettersson, Sara Bergström Lind

**Affiliations:** 1 Department of Chemistry-BMC, Analytical Chemistry, Uppsala University, Uppsala, Sweden; 2 The Beijer Laboratory, Department of Immunology, Genetics and Pathology, Science for Life Laboratory, Uppsala University, Rudbeck Laboratory, Uppsala, Sweden; University of St Andrews, UNITED KINGDOM

## Abstract

Viral infections cause large problems in the world and deeper understanding of the disease mechanisms is needed. Here we present an analytical strategy to investigate the host cell protein changes during human adenovirus type 2 (HAdV-C2 or Ad2) infection of lung fibroblasts by stable isotope labelling of amino acids in cell culture (SILAC) and nanoLC-MS/MS. This work focuses on early phase of infection (6 and 12 h post-infection (hpi)) but the data is combined with previously published late phase (24 and 36 hpi) proteomics data to produce a time series covering the complete infection. As many as 2169 proteins were quantitatively monitored from 6 to 36 hpi, while some proteins were time-specific. After applying different filter criteria, 2027 and 2150 proteins were quantified at 6 and 12 hpi and among them, 431 and 544 were significantly altered at the two time points. Pathway analysis showed that the *De novo purine and pyrimidine biosynthesis*, *Glycolysis* and *Cytoskeletal regulation by Rho GTPase* pathways were activated early during infection while inactivation of the *Integrin signalling* pathway started between 6 and 12 hpi. Moreover, upstream regulator analysis predicted MYC to be activated with time of infection and protein and RNA data for genes controlled by this transcription factor showed good correlation, which validated the use of protein data for this prediction. Among the identified phosphorylation sites, a group related to glycolysis and cytoskeletal reorganization were up-regulated during infection. The results show specific aspects on how the host cell proteins, the final products in the genetic information flow, are influenced by Ad2 infection, which would be overlooked if only knowledge derived from mRNA data is considered.

## Introduction

A virus infection consists of the entry of the virion into the host cell, the translation of viral mRNA by the host ribosomes, the replication of the viral genome, the assembly of the viral particles enclosing the genome, and the release of the infectious particles from the cell. Human adenoviruses (HAdVs) are non-enveloped icosahedral viruses with linear double stranded DNA genomes of 30–38 kb. They are among the most efficient DNA viruses that replicate in cell culture and therefore a good model system of infection. To ascertain an efficient production of progeny virus, HAdVs counteract the host cell antiviral defence and create an optimal condition for their DNA replication. For this purpose, HAdVs encode several proteins within the early regions: E1A, E1B, E3, and E4. The E1A proteins act as promiscuous transcriptional activators or repressors of both cellular and viral genes [1, 2, 3], and they are essential for forcing the host cell to enter the S phase. Two E1B proteins, E1B-55K and E1B-19K, play major roles in the counteraction of the pro-apoptotic program [4, 5]. Various proteins encoded in the adenovirus E3 transcription unit interact with the host immune system for maintaining cell viability [6]. Proteins encoded in the E4 transcription unit are mainly involved in the viral mRNA export from the nucleus, the viral DNA replication, and the host protein synthesis shut-off [7]. After the onset of HAdVs DNA replication, viral transcription switches from the early to the late mode. Concurrently, the host cell biomolecules are changed.

Several RNA-based technologies have revealed that human adenovirus type 2 (HAdV-C2 or Ad2) infection in human primary lung fibroblasts (IMR-90) can be divided into four periods, and the major changes in mRNAs, miRNA and lncRNA expression occur after 24 hours post-infection (hpi) [8, 9, 10, 11]. The first period is from 0 to 12 hpi, and during this time the viral gene expression begins. The first response to the incoming virus is most likely the growth suppression, since most of the observed regulated genes (RNAs) at early time points have functions linked to inhibition of cell growth [8]. The second period covers the time from 12 to 24 hpi, and it follows expression of the E1A gene. During this period, there are profound changes in the host cell gene expression that create an optimal environment for replication of the viral genome. At RNA level, about 50% of the altered genes are involved in cell cycle regulation, cell proliferation and antiviral response [9]. The third period extends from 24 to 36 hpi. By this time, the virus has gained control of the cellular metabolic machinery, resulting in an efficient replication of the viral genome. During the fourth and last period at 42 hpi, the cytopathic effect becomes apparent and the number of down-regulated genes increases dramatically including many genes involved in intra- and extracellular structures.

The fact that most studies on the host cell response to Ad2 infection are derived from mRNA studies limits our knowledge, since proteins are the final actors in cellular processes. Proteomes and their variations are efficiently studied by mass spectrometry-based methods [12]. Most proteomic studies report static snapshots, e.g. comparing control and infected conditions that provide valuable but limited information since proteomes dynamically respond in space and time. A better alternative is to use protein dynamics data, e.g. time series, to describe these processes [13, 14, 15]. Time series have been previously used to study the Herpes Simplex Virus Type 1 infection of human foreskin fibroblast [16], the macrophage response to Vesicular Stomatitis Virus infection [17], or the infection by H9N2 influenza virus in a human gastric carcinoma cell line [18], but also to investigate essential cellular functions [19]. Among the different strategies developed for the comparison of protein changes [20], the incorporation of stable isotopes using metabolic labelling of amino acids in cell culture (SILAC) has demonstrated to decrease the errors in quantification, and it offers good precision when comparing biological samples [21]. However, some additional problems at the MS quantification levels can be observed due to the incomplete incorporation of isotopic amino acids or experimental mixing problems. These problems can be solved using computational approaches [22, 23], or experimental corrections such as including SILAC label-swap replicates [24]. Different bioinformatics, statistical and graphical software have been developed to extract the relevant biological information from these analyses [25], and to visualize the massive amount of data generated by these high-throughput techniques [26]. SILAC in combination with LC−MS/MS has been used in Ad2-studies to investigate the protein degradation after inactivation of the virus by sunlight and UVC light [27], the quantitative changes in the protein composition of the nucleolus during infection [28], the temporal characterization of the non-structural proteome and phosphoproteome of Ad2 [29], or to develop a strategy to identify proteins using transcriptomic data from adenovirus type 5 (Ad5) infection of HeLa cells [30]. We have previously used this technology to study the host cell protein regulation during the late phase of infection [31]. This study demonstrated that at 24 hpi, the up-regulated proteins were related to the carbohydrate and nucleoside metabolism, and at 36 hpi, these proteins are also involved in protein translation, and protein and DNA metabolic processes. Proteins involved in cellular structures and in the integrin mediated cell signalling were down-regulated. Using the data from all the proteins and their corresponding mRNA, an overall low correlation (≈ 0.3) was observed [31]. This result is not surprising as it has been observed and discussed [30, 32, 33, 34]. The low correlation makes the prediction of the protein changes based on the use of RNA data extremely uncertain and it is therefore highly relevant to study the protein changes at specific times during Ad2 infection with separate technologies.

The aim of the present study is to characterize the host cell protein regulation induced by Ad2 infection using SILAC and high-resolution nanoLC−MS/MS at early and late phases. Stringent cut-off levels were applied to certify high quality data, and different bioinformatics and statistical tools were developed and combined for the identification of biological functions, the visualization of the protein regulation in the different pathways, and the analysis of the altered transcription factors affected during the infection process.

## Materials and methods

### Cell culture, infection and sample harvest

Human lung fibroblast (IMR-90) were purchased from American Type Culture Collection and cultured in 10 cm^2^ plates at the same conditions as previously described [31]. After six cell doublings and when the confluence was reached, cells were kept for 2 days at 37 °C for synchronization by growth inhibition. Then, 16.8 x 10^6^ labelled cells were either mock infected (only medium) or infected with Ad2 at a multiplicity of infection of 100 fluorescence-forming units/cell in 1 mL in serum free medium for 60 min. Afterwards, viruses were removed, and the cells were collected at 6, 12, 24 and 36 hpi. All samples were harvested at the same occasion, but late time points (24 and 36 hpi) were used in our previous study. A biological replicate in form of a swap-labelling experiment was performed. Cells were washed with PBS and directly snap frozen on dry ice.

### Cell lysis and sample preparation before LC–MS/MS

Cells were lysed as previously described [31]. Briefly, for 6 and 12 hpi, L labelled mock samples were mixed with H labelled cells infected with Ad2 in a 1:1 ratio, or the labels were swapped to obtain biological replicates at the same time points. Samples were subjected to SDS-PAGE, stained by Colloidal Coomassie Blue, and gel lanes were cut into 10 pieces and subjected to in-gel digestion [35]. Protein digestion was performed by trypsin 12.5 ng/μL (Promega Corporation, Madison, WI, USA) in 50 mM AmBi buffer at 37 °C overnight. Resulting peptides were extracted by acetonitrile and concentrated before analysis by LC–MS/MS.

### NanoLC–MS/MS

Two biological replicates with swapped amino acid labels were used to validate the reproducibility of the data. These biological replicates were preferred over technical replicates, and 40 samples were analysed once in the LC–MS/MS. The peptide separation was performed as previously described using the same instrumentation and parameters [31].

The raw data from 6 and 12 hpi, and the previously published raw data from 24 and 36 hpi (PRIDE Accession number: PXD004095), were processed together using MaxQuant (1.4.1.2)[36] and database searches were performed using the implemented Andromeda search engine [37]. MS/MS spectra were correlated to the Uniprot human database (release 2017–02, 156787 entries) combined with a human Ad2 database (release 2017–02, 560 entries). False discovery rate (FDR) was calculated based on reverse sequences from the target-decoy search, and an FDR of 1% was accepted for protein and peptide identification. For data processing, only peptides with a minimum of 7 amino acids and two maximum miss cleavages were accepted, and the mass tolerance was 4.5 ppm for the main search and 20 ppm for the fragment masses. Trypsin was selected as digesting enzyme, carbamidomethylation of cysteines as fixed modification, and oxidation of methionine, acetylation of the protein N-terminus and phosphorylation (STY) as variable modifications. For protein identification, at least one unique peptide and two peptides were required. For SILAC labelling quantification, Lys8 and Arg10 were set for heavy labels, and two ratio counts was the minimum. The mass spectrometry proteomics data have been deposited to the ProteomeXchange Consortium via the PRIDE [38] partner repository with the data set identifier PXD008980. The data for protein identification and quantification is provided in S1 Table.

### Statistical and bioinformatics analysis

After MaxQuant analysis, the “proteinGroups.txt” file obtained was loaded to the Perseus software (http://141.61.102.17/perseus_doku/doku.php?id=start). Prior to any statistical analysis, identifications flagged as reverse, potential contaminants, or proteins identified only by site were excluded for further analysis. MaxQuant automatically normalized each data set to the median of the ratios to correct for the mixing of H and L labelled cells at 1:1 ratios, and the values were transformed to the log2 scale for a better comparison between the different conditions. Principal Component Analysis (PCA) was carried out using Statistica software, and Pearson’s correlation test was performed using Perseus. To identify the proteins significantly altered/regulated, a 1.5-fold change cut-off (up- or down-regulated, equivalent to 0.585 and -0.585 in log2 scale) for each replicate was applied, and the average of the two replicates was obtained. The lists of significantly altered proteins were then uploaded into the web-based Panther software (http://www.pantherdb.org/) and into Ingenuity Pathway Analysis software (IPA; Qiagen, Redwood City, CA) to perform different analyses. Using Panther software, the overrepresented pathways in the lists of proteins were identified, and p-values were calculated using the Fischer’s exact test and considered significant when < 0.05. Moreover, IPA was used to perform a causal Upstream Regulator (UR) analysis, where a transcription factor state (activation or inactivation) is predicted based on a Fisher’s exact test between the list of proteins and the published targets of this regulator found in the literature. RNA data from a previous publication [31] was used for correlation of protein and RNA in the UR analysis.

After the functional annotation, and to generate heat maps, the log2-values of the proteins involved in the identified Panther pathways were plotted by hierarchical clustering. Thereafter, the values were normalized (subtracting the mean value of each data set and dividing by its standard deviation) and the Euclidean distances with respect to the centre were calculated and represented using box-plot graphs using GraphPad Prism software. In box-plot graphs, Euclidean distances are represented by different colours (red < 1; orange, 1–1.5; yellow 1.5–2; green, > 2).

Phosphopeptides were studied using the “Phospho (STY) sites.txt” file generated by MaxQuant. Identifications flagged as reverse or potential contaminants were excluded, and only phosphorylated sites with a localization probability > 0.75 were considered. To identify phosphorylated sites significantly altered, a 1.5-fold change cut-off (equivalent to 0.585 and -0.585 in log2 scale) for each replicate was applied, and the average of the two replicates was obtained. The data for the phosphorylated site identification and quantification is provided in S2 Table.

## Results and discussion

### Study of cellular protein regulation during early phase of Ad2 infection using SILAC-MS technology

**Fig 1:**
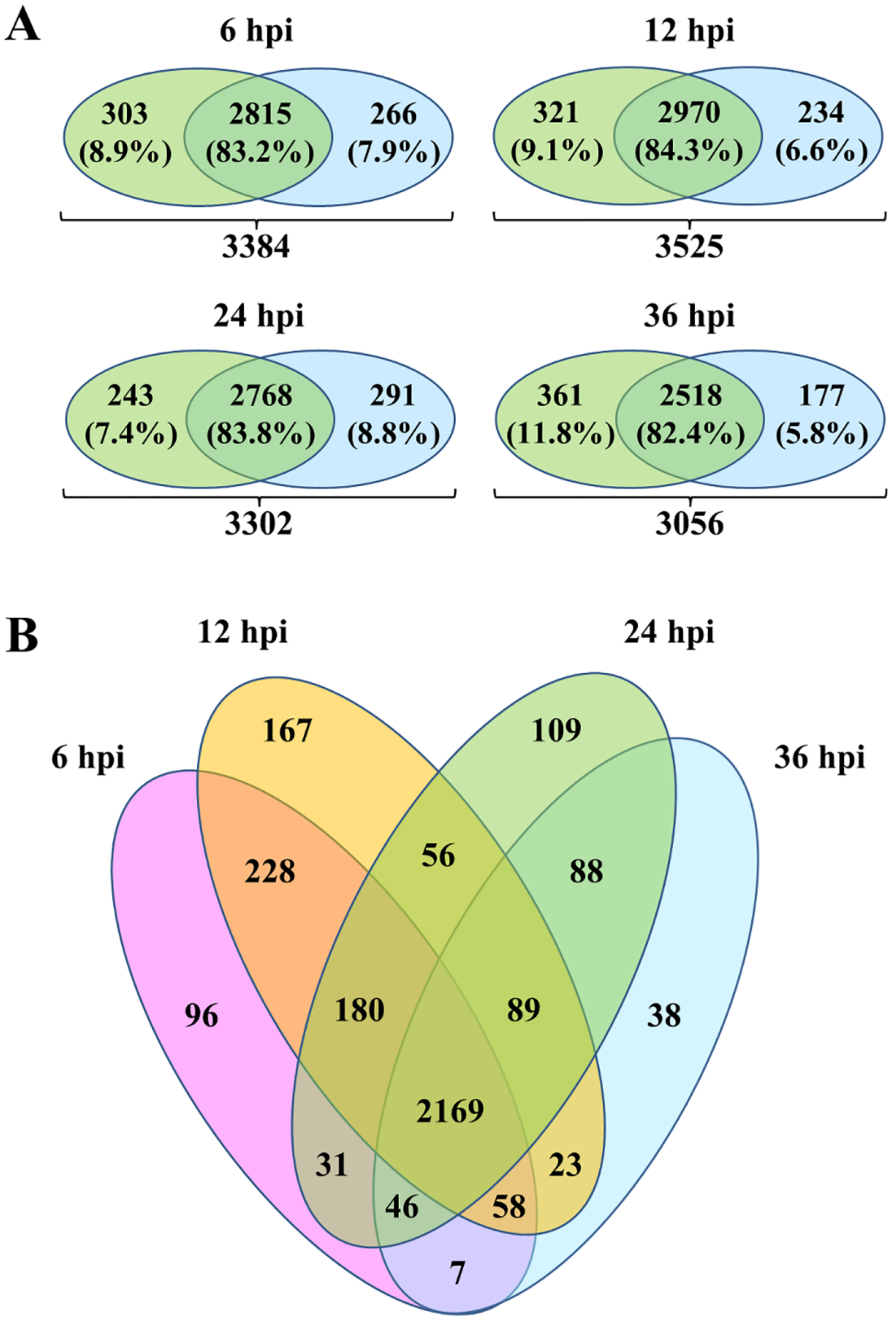
Overview of quantified host cell proteins in IMR-90 cells post Ad2 infection at 6, 12, 24 and 36 hours post-infection (hpi). (**A**) Overlap of proteins quantified in the SILAC label-swap replicates at different time points. (**B**) Venn Diagram of quantified proteins in both biological replicates in at least one time point.

As a first step, the incorporation efficiency/degree of heavy lysine and arginine was evaluated and was > 97% after five passages before infection. The infection process was then studied in time series with four time points and two biological replicates as swap labelling experiments, as previously suggested [24]. Proteins from Ad2(Light)/Mock(Heavy) and Mock(Light)/Ad2(Heavy) were combined 1:1, fractionated using SDS-PAGE, in-gel digested, and analysed by nano LC–MS/MS analysis to obtain SILAC protein ratios for accurate relative quantification of proteins. Using data from all four time points and applying a FDR of 1%, 4591 proteins were identified (S1 Table). An average of 3300 proteins could be quantified in at least one biological replicate at each time point (Fig 1A **and** Table 1) and among them, more than 82% were quantified in both biological replicates at each time point. This proved good reproducibility of the technology used. The results were comparable to previous studies by us and by Evans et al [30, 31]. In the latter, HeLa cells were infected by Ad5 and the cellular proteome was evaluated after 8 and 24 hpi. Even though the samples were fractionated in mores slices than in the present work (14 instead of 10) and a longer analytical column was used (250 instead of 100 mm), a similar number of proteins were quantified [30]. As presented in the Venn diagram (Fig 1B), 2169 proteins (64% of the quantified proteins) could be quantified at all time points, while some proteins were time-specific.

**Table 1:** Number of quantified proteins and phosphorylated sites in IMR-90 cells after Ad2 infection at 6, 12, 24 and 36 hours post-infection (hpi).

Parameter	6 hpi	12 hpi	24 hpi	36 hpi
Proteins in Ad2(L)/Mock(H)	3118	3291	3011	2879
Proteins in Mock(L)/Ad2(H)	3081	3204	3059	2695
Proteins in both biological replicates	2815	2970	2768	2518
Proteins with consistent regulation*	2027	2150	2360	2102
Significantly altered proteins (Ratio ≥ 1.5)	420	521	707	527
Significantly altered proteins (Ratio ≤ 0.67)	11	23	101	187
Phosphorylated sites in Ad2(L)/Mock(H)	48	76	55	71
Phosphorylated sites in Mock(L)/Ad2(H)	54	58	51	25
Phosphorylated sites in both biological replicates	30	36	27	16
Phosphorylated sites with consistent regulation*	15	20	21	9
Significantly altered phosphorylated sites (Ratio ≥ 1.5)	2	3	6	3
Significantly altered phosphorylated sites (Ratio ≤ 0.67)	0	1	4	5

* Proteins or phosphorylated sites quantified with the same direction of regulation in both biological replicates.

**Fig 2:**
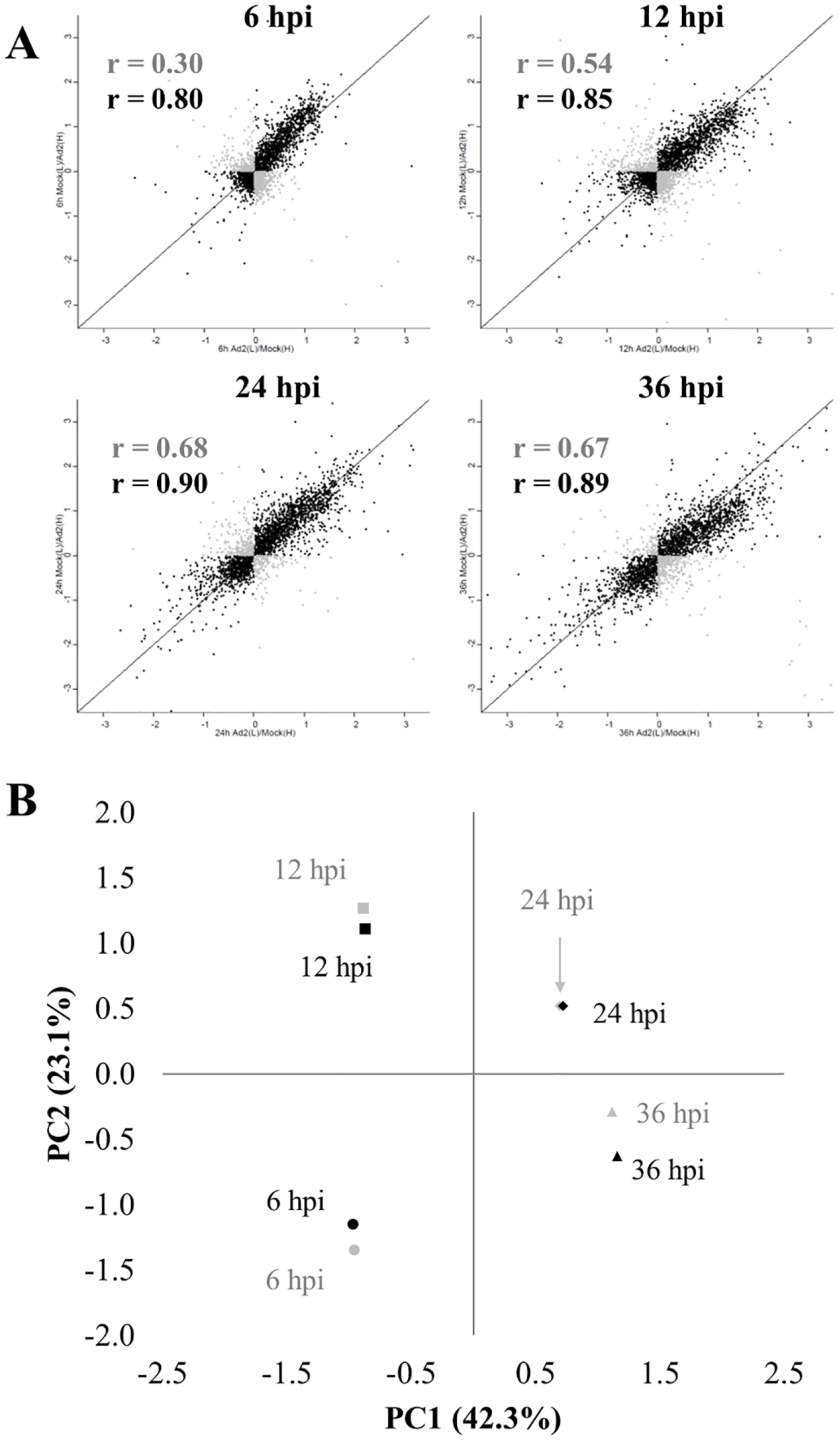
(**A**) Correlation between biological replicates after Ad2 infection at 6, 12, 24 and 36 hours post-infection (hpi): **Grey**, proteins quantified in both replicates; **Black**, proteins quantified in both replicates with consistent regulation. (**B**) Principal Component Analysis of quantified proteins in Ad2(L)/Mock(H) (**Grey**) and Mock(L)/Ad2(H) (**Black**) samples, in at least one time point after removing inconsistently regulated proteins between replicates.

Thereafter, the correlation between the two biological replicates at each time point was evaluated using the Pearson’s correlation test. Using all proteins quantified in both replicates (Fig 1A), the obtained Pearson’s correlation coefficient was low (r = 0.30) at 6 hpi, and medium (r = 0.54) at 12 hpi. However, the correlation increased at late time points (r = 0.68 for 24 hpi and r = 0.67 for 36 hpi) (Fig 2A, in grey). To increase the reliability of the protein quantification, different filtering criteria were applied. Firstly, proteins with opposite regulation profiles between the two biological replicates were removed from the analyses in order to only provide trustful biological data (Table 1). Resulting from this filtering, the Pearson’s correlation coefficient increased substantially (Fig 2A, in black), being > 0.80 in all time points. The number of proteins not passing the filtering criteria at early time points (≈ 800) was higher than the number of proteins removed at late time points (≈ 400) (Table 1). These results suggest that the changes in host proteome are more variable during early phase of infection and that the infection stabilizes during the progression of the infection, giving more reproducible proteomes at late time points. The number of proteins considered at 24 and 36 hpi was lower than in our previous study [31], since this combined analysis of all time points is more stringent. For instance, 60% of the proteins removed due to inconsistent changes between replicates had a fold change between 0 and ± 0.1 (in log2 scale) in at least one of the replicates. Such small changes should not be used for biological conclusions. As a result, higher correlation values were obtained (0.90 at 24 hpi and 0.89 at 36 hpi), compared to the previous ones (0.81 and 0.89) [31]. Proteins passing this first filtering criterion were analysed by PCA to determine, compare and visualize the overall relation between the four conditions studied (Fig 2B). This analysis indicated that the combination of the two main components, PC1 and PC2, captured more than 65% of the variance of the data. PC1 could separate the samples representing 6 and 12 hpi vs. 24 and 36 hpi, while PC2 could distinguish the two different early time points (6 and 12 hpi), and also the two different late time points (24 and 36 hpi).

Secondly, and in agreement with other publications [15], a 1.5-fold change cut-off (equivalent to 0.585 and -0.585 in log2 scale) was applied to identify the significantly altered/regulated proteins at each time point (Table 1). In total, approximately 25% (at early phase) and 35% (at late phase) of the quantified proteins were altered with this fold change, and their ratios can be found in Part A-D of S3 Table. During Ad5 infection in HeLa cells, only 1% and 8% of the proteins showed either twofold-or-greater change in protein abundance at 8 and 24 hpi, respectively [30]. These differences could, in addition to the higher cut-off level be explained by the varying cell models used in the experiments, since transcriptomics studies have demonstrated that more cellular genes are differentially expressed in IMR-90 than in HeLa cells during the course of adenovirus infection [8, 39, 40]. The replication of adenovirus in HeLa cells is also extremely efficient, and therefore the time window for examination of the details of cellular gene expression in this cell line is narrower than in IMR-90 cells [8, 39, 40]. This could also affect the protein regulation. However, the most relevant proteins, quantified during Ad5 infection of HeLa cells (HSPA1A, MRE11A, ITGA3, RAD50, POLDIP3), were similar (S1 Fig) to Ad2 infection of IMR-90 cells.

### Progression of the cellular protein regulation during infection

After the data filtering, different groups/clusters of proteins were formed according to their altered abundances.

#### Proteins uniquely altered at 6 hpi

Among the 431 proteins significantly altered at 6 hpi, 40 were unique compared to 12, 24 and 36 hpi data (Part A of S3 Table). Of them, 39 and 1 had positive and negative ratios, respectively. Enrichment analysis of these proteins did not reveal any overrepresented pathways due to the small number. However, by searching for functions using STRING database (https://string-db.org) and Genecards (https://www.genecards.org), it became apparent that some of them have interesting functions. For example, DSC1 is involved in cell-to-cell adhesion; ACTR1B and TRIP6 in actin polymerization and cytoskeletal organization; and KIF5B and TWF2 are microtubule related proteins. Other proteins are involved in protein folding (PPIL3), prevention of protein aggregation (CRYAB) or in the nucleolar-cytoplasmic transport (TCOF1). One especially interesting protein is BNIP2 (also known as BCL2/adenovirus E1B interacting protein). BNIP2 interacts with the adenovirus E1B 19 kDa protein, which has been implicated in the protection against the cell death program induced by viral infection [41]. The only supressed protein was CYR61. CYR61 is one of the six extracellular matrix-associated proteins (CCN family), and it plays diverse roles in cell proliferation, survival and migration through interactions with cell adhesion receptors, including integrins [42, 43]. Specifically, CYR61 binds to integrin αvβ3 to mediate endothelial cell adhesion [44] and can inhibit cell proliferation and down-regulate the mRNA expression of COL1A1 in normal human fibroblasts [45]. Integrin αvβ3 has been also reported to be used by adenoviruses for internalization [46, 47]. Moreover, CYR61 can also interact with ITGB5, a protein which becomes supressed from 6 to 24 hpi. ITGB5, together with ITGAV, has been demonstrated to act as primary receptor in Coxsackievirus and Adenovirus receptor (CAR)-negative cells [48]

#### Proteins uniquely altered at 12 hpi

Of the 544 proteins significantly altered at 12 hpi, 57 (54 and 3 had positive and negative ratios, respectively) were uniquely altered in comparison with the other three time points (Part B of S3 Table). The *Cytoskeletal regulation by Rho GTPase*, the *Inflammation mediated by chemokine and cytokine signalling pathway* and the *Integrin signalling pathways* were overrepresented among these proteins. Even though these proteins were uniquely altered at this time points, their corresponding pathways were also active at later time points and will therefore be described in the following sections.

#### Proteins uniquely altered at both 6 and 12 hpi

To compare early versus late time points of infection, protein data from 6 and 12 hpi were merged and compared with merged 24 and 36 hpi data. The set of proteins significantly altered only at early time points included 48 proteins (47 and 1 with positive and negative ratios, respectively) (Part C of S3 Table). All these proteins showed the same direction of regulation at 6 and 12 hpi. The functional enrichment analysis indicated an overrepresentation of the *Cytoskeletal protein binding* and the *Structural constituent of cytoskeleton* molecular functions. Other altered proteins are involved in the regulation of glutathione metabolism and detoxification processes (GCLC, GLRX, TXNDC17, GSTM3, TXN), in the organization of the cytoskeleton (FSCN1, TUBB2B, TBCA, TUBB3, TMSB4X, PFN1) or the proteasome system (UBE2L3, UCHL1), and in signalling pathways such as TGF-β (FKBP1A), Rho (ARHGDIA, GDI1, GDI2) and NF-κB (MTPN). These signalling pathways have also been observed as altered at the transcriptome level [9]. We and other groups have pointed out the stress response protein HSPA1A/B as the protein with the highest change at late phase of infection [31, 49]. However, the ratio of this protein was not significant at 6 hpi (0.41 in log2 scale), and it was mildly altered at 12 hpi (0.73 in log2 scale). Similarly, the ratio of mini-chromosome maintenance (MCM) proteins was significantly positive late, but not in the early phase. Conversely, the proteins with the lowest negative ratio at 6 and 12 hpi were collagen proteins (COL1A1, COL1A2, COL3A1, COL5A1, COL5A2, COL12A1) and related proteins such as PCOLCE, which drives the enzymatic cleavage of type I procollagen, and TIMP3, which can form complexes with collagenases. In most cases, similar negative ratios were observed at later time points. In addition to the CYR61 protein mentioned above, another protein involved in cell-to-cell interaction (THBS1) was less abundant, which fully agrees with previous mRNA results [9]. Other proteins involved in cell adhesion also had negative ratios: CSPG2 and CTGF. CTGF is another member of the CCN family, and it mediates cell adhesion, directional migration, and proliferation through integrin αvβ3 [50]. It is also related with the platelet-derived growth factor receptor PDGFRA, a protein with a negative ratio from 6 to 36 hpi. Moreover, a negative ratio was observed for SERPINE1, the principal inhibitor of tissue plasminogen activator and urokinase, as well as for the two proteins that are under its control, PLAT and PLAU.

### Pathway analysis (Functional annotation)

The lists of proteins significantly altered at each specific time were simultaneously analysed using the overrepresentation function included in the web-based software Panther. The significantly overrepresented pathways (p-value < 0.05) in at least one time point are shown in Table 2, and if so, the values for the other time points are presented for comparison.

**Table 2:** Overrepresented pathways in IMR-90 cells after Ad2 infection at 6, 12 24 and 36 hours post-infection (hpi).

Panther Pathway	N° proteins (reference)	6 hpi			12 hpi			24 hpi			36 hpi		
		N° proteins	Enrichment	p-value	N° proteins	Enrichment	p-value	N° proteins	Enrichment	p-value	N° proteins	Enrichment	p-value
Serine glycine biosynthesis	5	3	29.5	**2.6e-02**	3	23.5	5.0e-02	3	15.8	1.6e-01	1	6.0	1.0e+00
Mannose metabolism	6	3	24.6	**4.4e-02**	3	19.6	8.5e-02	3	13.2	2.7e-01	2	10.0	1.0e+00
Gonadotropin-releasing hormone receptor pathway	236	8	1.7	1.0e+00	18	3.0	**8.6e-03**	20	2.2	1.5e-01	18	2.3	2.1e-01
PDGF signalling pathway	149	10	3.3	1.8e-01	13	3.4	**2.6e-02**	12	2.1	1.0e+00	9	1.8	1.0e+00
Insulin/IGF pathway-mitogen activated protein kinase/MAP kinase cascade	33	3	4.5	1.0e+00	6	7.1	**3.8e-02**	5	4.0	1.0e+00	5	4.5	8.8e-01
Fructose galactose metabolism	12	3	12.3	3.2e-01	4	13.0	**4.6e-02**	4	8.8	2.0e-01	3	7.5	1.0e+00
Toll receptor signalling pathway	57	5	4.3	1.0e+00	10	6.9	**4.8e-04**	9	4.2	6.6e-02	6	3.1	1.0e+00
Ubiquitin proteasome pathway	62	7	5.6	5.2e-02	6	3.8	9.1e-01	11	4.7	**5.6e-03**	7	3.4	8.7e-01
Pentose phosphate pathway	8	2	12.3	1.0e+00	2	9.8	1.0e+00	4	13.2	**4.4e-02**	2	7.5	1.0e+00
Cytoskeletal regulation by Rho GTPase	82	11	6.6	**2.2e-04**	16	7.6	**1.3e-07**	9	2.9	7.6e-01	6	2.2	1.0e+00
CCKR signalling map	173	10	2.8	5.3e-01	20	4.5	**6.6e-06**	19	2.9	**8.5e-03**	14	2.4	4.1e-01
Ras Pathway	74	6	4.0	7.1e-01	12	6.3	**1.1e-04**	11	3.9	**2.6e-02**	9	3.6	1.7e-01
Angiogenesis	174	11	3.1	1.7e-01	15	3.4	**9.4e-03**	18	2.7	**2.7e-02**	13	2.2	1.0e+00
De novo pyrimidine deoxyribonucleotide biosynthesis	14	2	7.0	1.0e+00	2	5.6	1.0e+00	5	9.4	**3.6e-02**	6	12.8	**1.6e-03**
FGF signalling pathway	122	12	4.8	**1.7e-03**	19	6.1	**1.3e-07**	20	4.3	**1.5e-05**	10	2.5	1.0e+00
EGF receptor signalling pathway	139	12	4.3	**6.0e-03**	19	5.4	**1.1e-06**	22	4.2	**6.3e-06**	10	2.2	1.0e+00
Integrin signalling pathway	194	9	2.3	1.0e+00	20	4.0	**4.0e-05**	26	3.5	**9.9e-06**	26	4.0	**8.6e-07**
Glycolysis	20	11	27.1	**1.2e-10**	13	25.4	**2.3e-12**	13	17.1	**3.3e-10**	12	17.9	**1.3e-09**
De novo purine biosynthesis	31	11	17.5	**1.3e-08**	12	15.1	**8.9e-09**	17	14.4	**2.1e-12**	14	13.5	**1.1e-09**
De novo pyrimidine ribonucleotides biosynthesis	14	5	17.6	**1.9e-03**	5	14.0	**5.7e-03**	5	9.4	**3.6e-02**	5	10.7	**2.0e-02**

In grey, significant overrepresented pathways (p-value < 0.05).

#### 6 hpi pathways

Among the identified pathways, two were only found overrepresented at 6 hpi: the *Serine glycine biosynthesis* and the *Mannose metabolism*. Even though the ratios of the proteins considered in these pathways were significant (and positive) at 12 and 24 hpi (and some of them at 36 hpi), these pathways were only overrepresented at 6 hpi because other pathways were more significant at later time points. In the case of *Serine glycine biosynthesis* pathway, the proteins PSAT1, PHGDH, and PSPH were considered. These proteins are involved in the conversion of the glycolytic intermediate 3-phosphoglycerate into serine, which can then be transformed into glycine. In the case of *Mannose metabolism* pathway, the proteins GMPPA, PMM2, and GMPPB are involved in the generation of GDP-mannose, which contribute to N-glycosylation, O-glycosylation, C-mannosylation, and GPI anchor synthesis [51].

#### 12 hpi pathways

**Fig 3:**
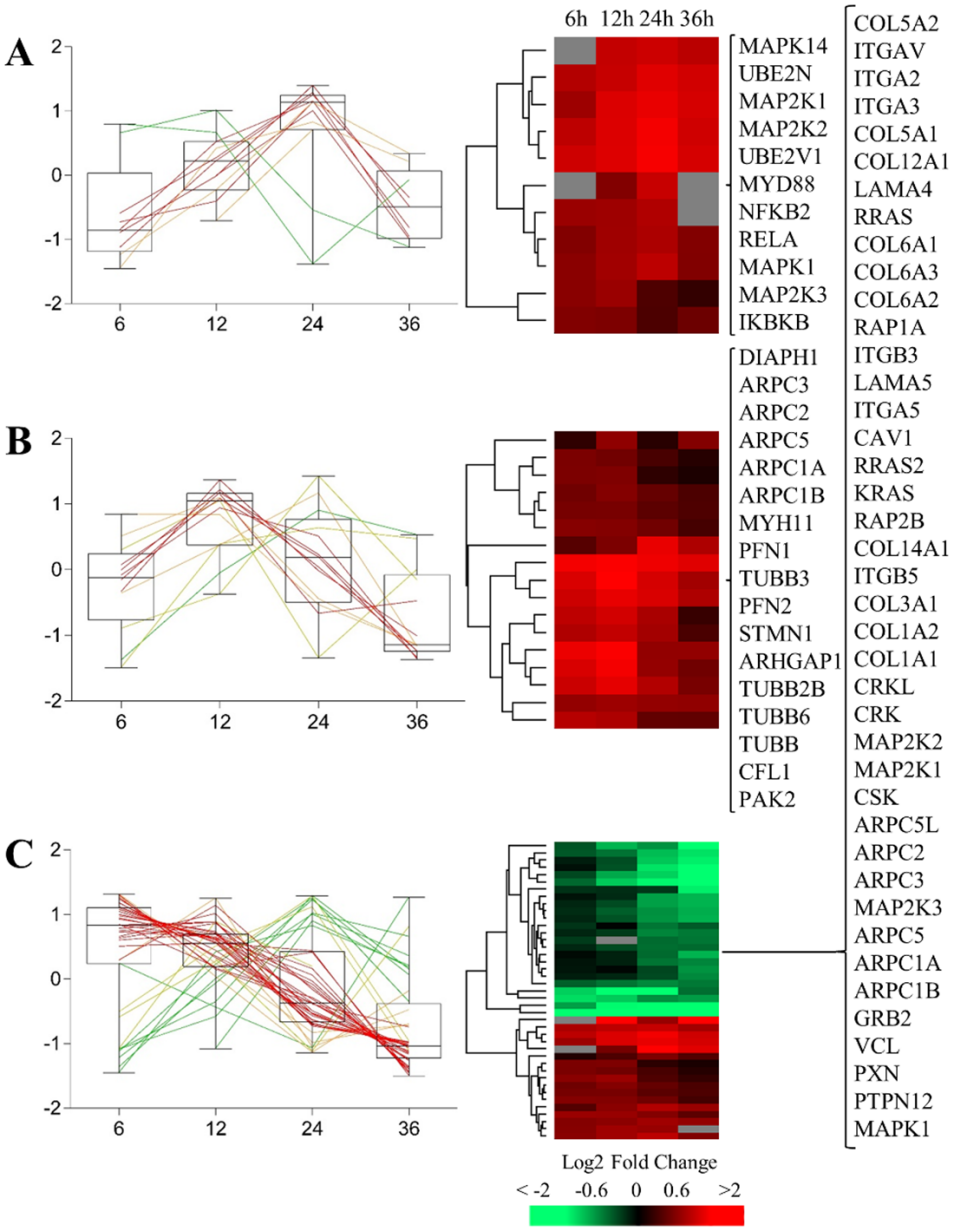
Box-plot graph and hierarchical clustering after Ad2 infection at 6, 12 24 and 36 hours post-infection (hpi) of the proteins involved in: (A) the *Toll Receptor pathway*, (B) the *Cytoskeletal regulation by Rho GTPase pathway*, and (C) the *Integrin signalling pathway*.

Different pathways were uniquely overrepresented at 12 hpi, such as the *PDGF signalling pathway*, the *Fructose galactose metabolism*, and the *Toll receptor signalling pathway*. In these cases, 13, 4 and 10 proteins were significantly altered, respectively. The *Toll receptor signalling pathway* was the most significantly overrepresented at this time, but as presented in Fig 3A, the overrepresentation was even higher at 24 hpi. In this pathway, several MAP kinases (MAPK1, MAP2K1, MAP2K2, MAPK14, MAP2K3), ubiquitin-conjugating enzymes (UBE2V1 and UBE2N), the NF-kappa-B subunits 2 (NFKB2) and 3 (RELA), and the Inhibitor of nuclear factor kappa-B kinase subunit beta (IKBKB), had positives ratios. Toll-like receptors play critical roles in the innate immune system by recognizing pathogen-associated molecular patterns derived from various microbes, such as adenoviruses [52, 53]. Upon the activation of the receptors, the proteins UBE2V1 and UBE2N can form a heterodimer that acts in concert with TRIM5 to activate the MAP3K7/TAK1, which in turn phosphorylates and activates IkB kinase (IKK), leading to the activation of NF-kB [54, 55].

Another interesting pathway overrepresented at 12 hpi was the *PDGF signalling pathway*. This pathway is initiated upon binding of PDGF to the PDGF receptor complex. Different homodimers of A-, B-, C- and D-polypeptide chains, and the heterodimer PDGF-AB can be formed, which are synthesized as precursor molecules and must be activated by cleavage [56]. In the case of PDGF-CC and–DD homodimers, the activation is performed by the tissue-type plasminogen activator (PLAT), and in the case of PDGF-DD, by the urokinase-type PA (PLAU). Both PLAT and PLAU had negative ratios at 12 hpi, indicating that the cell is trying to minimize the activation of the *PDGF signalling pathway*. Once activated, the PDGF receptors create docking sites for signalling molecules, such as the signal transducers and activators of transcription (STATs). However, these receptors also bind adaptor molecules which form complexes with other signalling molecules, such as the regulatory subunit p85 of the phosphatidylinositol 3′-kinase (PI3K), and GRB2, which binds the nucleotide exchange molecule SOS1, activating RAS and the ERK MAP-kinase pathway. The activation of PI3K can also lead to actin reorganization via the GTPase activator for the Rho, Rac and Cdc42 proteins (ARHGAP1). These proteins were found with a positive ratio in the early phase. Furthermore, the PDGF receptors can interact with other receptors, such as the EGF receptor [57] or, integrins [58]. The pathways controlled by these receptors were also found overrepresented in this study (Table 2), but the receptors had negative ratios in the late phase. In accordance with the regulation of theses receptors, the Platelet-derived growth factor receptor alpha (PDGFRA) had a negative ratio at all time points of the infection, and the beta receptor (PDGFRB) only at late time points, suggesting that the cell is trying to shut-off all these pathways to impede the ongoing infection.

The *Fructose galactose metabolism* function was also highly enriched, and the ratios of 4 proteins (ALDOA, ALDOC, GALM, and GALE) out of the 12 contained in this pathway were positive. These proteins are involved in the production of precursors that are used in the glycolysis cycle for energy production. Moreover, the *Gonadotropin-releasing hormone receptor pathway* and the *Insulin/IGF pathway-mitogen activated protein kinase/MAP kinase cascade* were also overrepresented. However, the proteins considered in these pathways are not pathway-specific, therefore these results were regarded as false positives.

The most interesting pathway overrepresented at early time points was the *Cytoskeletal regulation by Rho GTPase*. This pathway has been deeply studied during adenovirus infection [59]. After the attachment of adenovirus via CAR and/or the αv integrin, adenovirus endocytosis requires the reorganization of the host cell actin cytoskeleton. As commented above, PI3K is responsible of the activation of ARHGAP1, which activates the small GTP-binding proteins Ras, Rho, Rac, and CDC42. Rho GTPases also regulate certain mitogen-activated protein (MAP) kinase pathways following integrin or growth factor ligation [60]. Another interesting protein involved in the adenovirus internalization is PAK1, a member of the p-21 serine-threonine kinase family, which contains a high-affinity binding site for the GTP-bound form of Rac and CDC42 [61]. Binding of Rho GTPases to PAK1 or PAK2 results in autophosphorylation and activation of their kinase activity, leading to reorganization of the actin cytoskeleton. PAK2 also phosphorylates MAPK4 and MAPK6 and activates the downstream target MAPKAPK5, a regulator of F-actin polymerization and cell migration. Furthermore, the Arp2/3 complex is involved in regulation of actin polymerization. Together with the nucleation-promoting factor (PFN), it mediates the formation of branched actin networks [62]. Several components of the Arp2/3 complex were found with positives ratios during the infection (ARPC1A, ARPC1B, ARPC2, ARPC3, ARPC1A) as well as the PFN1 and PFN2 units. Different tubulin members (TUBB, TUBB2B, TUBB3, TUBB6) and STMN1 (involved in the regulation of the microtubule filament system) also had positive ratios. As it can be observed in Table 2, the number of proteins considered in this pathway was higher at 12 hpi, and for those found at both at 6 and 12 hpi, the ratios were higher at 12 hpi (Fig 3B). However, most of these proteins were not significantly altered at later time points. The functional annotation analysis performed in our previous work using transcriptomic data indicated that the most down-regulated mRNA at 24 hpi are involved in the regulation of cytoskeleton (such as RhoB, ARHGEF1, and ARHGAP22) [9]. The down-regulation of these mRNAs could explain the non-significant ratios observed at late time points, since it is known that mRNAs are less stable than proteins and it takes longer to degrade proteins [33].

#### 24 / 36 hpi pathways

When considering the late time points, we also identified the *Ras Pathway* as overrepresented at 12 and 24 hpi. This pathway plays a key role in transducing extracellular cues, and stimulating cellular proliferation, differentiation and survival. As we noted previously, this pathway could be triggered by the increased levels of GRB2 after activation of the *PDGF signalling pathway*, but the key protein KRAS was found with negative ratios at 24 and 36 hpi, as well as the protein RRAS2 at 36 hpi. In our previous study, we observed the overrepresentation of the *Pentose phosphate pathway* at 24 hpi at the protein level but not at the mRNA level [31]. In this combined study of all time points, the ratios of two proteins involved in this pathway were also higher than the threshold at 12 hpi (GPI and PGD). The *Pentose phosphate pathway* cooperates with glycolysis for energy production, indicating that the virus requires high amounts of nucleotides for its replication at late time points, and therefore it requires ribose-5-phosphate. Complementary to this pathway, the serine synthesized from the glycolytic intermediate 3-phosphoglycerate can be converted to glycine (an important precursor for purine biosynthesis), via the *Serine glycine biosynthesis* pathway [63]. Some proteins involved in this pathway (PSAT1, PHGDH, and PSPH) were also more abundant at 24 hpi. Because of its conversion to glycine, serine is also the donor of folate-linked one-carbon units which are required for nucleotide biosynthesis [64]. The *Pentose phosphate pathway* is tightly connected with the *de novo* synthesis of nucleotides [65] and we observed that the *De novo purine biosynthesis* and the *De novo pyrimidine ribonucleotides biosynthesis* were overrepresented at all time points, but with more proteins and higher ratios at late time points of infection (Table 2). Moreover, we observed the overrepresentation on the *Glycolysis* pathway at all time points, most strikingly at 12 hpi (Panel A in S2 Fig).

#### Time independent pathways

Several signalling pathways were also overrepresented in this study. Among them, the *FGF signalling pathway* and the *EGF receptor signalling pathway* were found significant at 6, 12 and 24 hpi while the *Integrin signalling pathway* was significant at all time points. The ratios of most of the proteins involved in the FGF and the EGF receptor signalling pathways were positive, but they decreased with time post infection (Panel B and C in S2 Fig). As well as for the regulation of the cytoskeletal function, the inactivation of these signaling pathways could be explained by the down-regulation of different growth factors at the mRNA level (such as FGF1, FGF2, CTGF or VEGFC) observed at 24 hpi [9]. On the other hand, several proteins involved in the *Integrin signalling pathway* had negative ratios, such as different integrins (ITGB5, ITGAV, ITGA5, ITGA2, ITGB3, ITGA3), collagens (COL1A1, COL3A1, COL1A2, COL12A1, COL5A2, COL5A1, COL6A2, COL6A1, COL6A3, COL14A1) and RAS family members (RRAS, RRAS2, KRAS). Fig 3C shows that, compared to mock infection, the negative ratios of the proteins in this pathway decreased with the time, and most of the genes encoding these proteins were also found down-regulated at 24 and 36 hpi at the mRNA level [31].

### Upstream regulator analysis

The UR analysis is a bioinformatic analysis that identifies upstream transcriptional regulators that are connected to gene or protein datasets through a set of direct or indirect relationships stored in the Ingenuity^®^ Knowledge Base. In the present work, our lists of significantly altered proteins were analysed with this tool and transcriptional regulators considered significant in at least one time point are reported in (Table 3).

**Table 3:** Upstream regulator analysis (IPA software) of IMR-90 cells after Ad2 infection at 6, 12 24 and 36 hours post-infection (hpi).

	6 hpi			12 hpi			24 hpi			36 hpi		
Transcription Factor	Activation z-score	p-value	Target molecules	Activation z-score	p-value	Target molecules	Activation z-score	p-value	Target molecules	Activation z-score	p-value	Target molecules
ARNT	2.4	1.3E-04	10	**2.6**	1.9E-04	11	1.5	1.9E-06	17	2.2	6.2E-08	18
ARNT2	**3.6**	2.5E-04	13	**3.1**	2.0E-03	13	**2.8**	6.8E-04	18	2.3	3.3E-03	15
ATF4	**3.8**	4.1E-07	15	**3.6**	6.7E-06	15	2.4	5.4E-05	17	0.8	1.5E-04	15
CREB1	**--**	--	--	--	--	--	**2.5**	2.0E-02	31	**2.6**	5.2E-04	34
E2F1	1.3	6.7E-07	28	1.4	6.7E-06	30	**3.1**	4.2E-16	60	**2.7**	9.6E-17	57
GATA1	-1.7	2.2E-03	12	-0.7	2.9E-02	11	**-2.5**	9.7E-04	19	-1.8	2.4E-05	21
GFI1	**--**	--	--	-2.4	3.7E-02	6	**-3.0**	1.1E-02	9	--	--	--
HNF1A	2.2	1.5E-03	19	**2.6**	5.4E-05	26	**2.6**	1.5E-02	26	--	--	--
HNF4A	**2.7**	1.5E-08	78	**2.9**	1.3E-12	104	**3.0**	1.8E-17	153	**2.6**	5.1E-13	128
IRF4	**-2.6**	2.7E-02	7	**-2.8**	3.1E-02	8	**-3.9**	3.7E-04	15	**-2.9**	3.3E-03	12
IRF7	--	--	--	--	--	--	**2.6**	4.3E-02	11	--	--	--
KLF3	**2.8**	1.4E-04	18	**-3.1**	3.0E-04	20	**-3.5**	3.5E-03	23	-2.0	4.6E-05	26
MEF2D	--	--	--	**--**	**--**	--	**-2.6**	7.8E-03	7	-2.4	1.6E-02	6
MYC	**4.9**	2.0E-23	78	**5.9**	1.1E-26	94	**6.5**	7.9E-36	134	**6.8**	2.6E-54	151
MYCN	**3.0**	9.0E-09	23	**3.6**	5.9E-13	32	**4.8**	2.0E-18	47	**6.4**	1.9E-27	55
NCOA3	2.2	1.3E+02	5	2.2	3.0E-02	5	**2.8**	4.2E-03	8	**2.8**	2.0E-03	8
NFE2L2	**4.9**	4.1E-13	35	**5.2**	3.5E-14	41	**6.0**	1.7E-12	48	**5.0**	2.6E-08	37
PPARGC1A	2.4	2.8E-03	12	**2.7**	3.5E-02	11	1.3	1.5E-02	16	1.0	4.7E-02	13
RB1	0.1	1.0E-02	15	0.1	8.1E-03	18	**-3.0**	1.4E-08	39	-2.2	1.7E-08	36
REL	**2.8**	4.6E-02	9	**3.3**	2.5E-03	14	**3.5**	3.0E-02	15	--	--	--
SIM1	**3.5**	1.1E-03	12	**2.9**	7.0E-03	12	**2.7**	2.4E-03	17	2.1	1.0E-02	
SMAD3	-1.7	4.4E-02	9	-1.2	5.9E-03	13	**-3.0**	1.2E-03	19	**-2.9**	4.5E-03	16
SPDEF	--	--	--	2.4	1.5E-03	7	**3.2**	6.6E-06	12	**3.5**	2.6E-07	13
SRF	**2.6**	1.2E-03	16	**2.6**	1.2E-04	21	2.4	1.8E-03	24	2.4	7.9E-03	20
STAT4	**3.5**	2.6E-06	17	**3.2**	5.1E-04	15	**3.1**	2.7E-04	20	1.1	1.5E-04	19
TCF7L2	**3.5**	1.4E-02	13	**3.2**	1.8E-02	15	2.1	2.1E-02	20	--	--	--
XBP1	**2.8**	7.7E-03	10	**2.9**	6.1E-04	14	--	--	--	--	--	--

In grey, transcription factors activated (z-score > 2.5) or inactivated (z-score < -2.5) with a p-value < 0.05.

This analysis was primarily developed to work with gene datasets, but it has also been useful for protein expression datasets [16, 66, 67]. Protein synthesis is a biosynthetic process downstream from transcription and its regulation. We have previously observed that the overall correlation between the RNA and the protein levels was low at 24 and 36 hpi (r ≈ 0.3) [31], and it has been observed even lower in other studies [30]. However, after the application of a 1.5-fold change cut-off at the protein level, the correlation significantly rose up (r ≈ 0.5). This strategy has previously been applied to increase the confidence for the use of differential mRNA for biological discovery [68]. The resulting correlation should reflect differences in mRNA and protein dynamics, as mRNAs are produced at a slower rate than proteins, and the mRNA turnover can be faster while the effect on the protein level may still prevail [34, 35]. Thus, the exact time at which transcription factors are activated/deactivated is difficult to determine based on the protein data. To validate our proteomics data used in the UR-prediction, the changes of those proteins controlled by altered transcription factors were correlated (by Pearson’s correlation test) with their corresponding RNA changes upon infection [31]. In this case, an acceptable correlation coefficient between proteins and RNAs > 0.7 was obtained, and the most relevant results are reported in Panel A in S3 Fig. This exemplifies that a good correlation between altered ratios of protein and RNA can be achieved when zooming in on specific processes, but not when considering all the processes. Similar results were noted for the correlations between mRNA and protein abundances for open reading frames (ORFs) that varied over the course of the cell cycle in yeast [69]. The correlation was observed higher (r = 0.89) for those ORFs that show a large degree of variation, while for ORFs with minimal variation, the correlation was poor (0.2).

Among all the transcription regulators included in Table 3, MYC was highly activated, and its activation increased with time. It has been shown that during adenovirus infection, the E1A adenovirus protein decreases MYC expression at the mRNA level, but not at the protein level [70]. Moreover, a later study demonstrated that stabilization of MYC requires the p400 protein, and that E1A promotes the association of MYC and p400 at MYC target genes, leading to induction of their transcription [71]. Different studies have characterized virus-induced changes in host cell metabolism [72], and it has been suggested that adenovirus E4orf1 binds to MYC and induces the expression of glycolytic target genes and nucleotide biosynthesis from glucose intermediates [73]. Therefore, the activation of MYC by E1A and E4orf1 adenovirus proteins could explain why the *Glycolysis* and the *Pentose phosphate pathways* were overrepresented in spite of the decrease in the MYC mRNA level.

Another transcription factor found activated at late time points was E2F1. We observed in our previous transcriptomic studies that the expression of several genes involved in cell cycle and DNA replication and controlled by the E2F were up-regulated at 12 hpi, caused by the interaction of E1A protein with retinoblastoma protein [9, 74]. Our proteomic data demonstrates that the ratios of several components of the minichromosome maintenance complex (MCM2, MCM3, MCM4, MCM5, MCM6, MCM7), some replication factors (RFC2, RCF3, RFC4, RCF5), and different cell cycle regulators (CDK2, CDKN2A), were consistent with the ratios for these genes at the mRNA levels at 24 hpi, but not at 12 hpi. It is therefore likely that the mRNA transcription starts at 12 hpi, but the effects in the protein levels are delayed and only observed at 24 hpi.

The transcriptomics data also revealed an up-regulation of genes controlled by the ATF/CREB family, predicting an overrepresentation of ATF1-4 and CREB1 at 24 hpi [9]. These transcription factors control the expression of genes involved in DNA and RNA metabolism, but also genes involved in the stress response. The UR analysis confirmed the activation of CREB1 at late time points but the activation of the transcription factor ATF4 could only be observed at early time points (6 and 12 hpi). The UR analysis also showed the activation of the transcription factor XBP1 at early time points. XBP1 is part of the Unfolded Protein Response (UPR) triggered by endoplasmic reticulum (ER) stress. Interestingly, different studies have demonstrated an ER stress response in virus infection [75]. XBP1 mRNA is specifically spliced and activated by the inositol-requiring kinase 1 (IRE1), which increases the transcription of chaperones and other proteins involved in vesicular trafficking and transport between the Golgi complex and the ER, such as the coatomer proteins. Many of these coatomer proteins (COPA, COPB1, COPE1, COPG1 and SEC31A), which had positive ratios mainly at early time points, have been suggested to play important roles in the infection by several viruses [76]. The UPR also generates excess levels of reactive oxygen species (ROS). One of the most important cellular defence mechanisms against ROS excess is controlled by the nuclear erythroid-related factor 2 (NFE2L2 or NRF2), highly activated at all time points in our study. NRF2 inhibits lipogenesis, activates the oxidation of fatty acids, simplifies the flux through the pentose phosphate pathway and increases NADPH regeneration and purine biosynthesis.

Finally, the UR analysis indicated that the RB1 transcription factor was inactivated after 24 hpi. Our previous transcriptomic study indicated that the RB1 mRNA level was increased at 24 hpi [9], which might due to the host cell trials in increasing its activity to combat the infection, possibly because of the E1A blockage. Moreover, adenoviruses can block apoptosis via two proteins: E1B-55K and E1B-19K. The E1B-19K protein blocks apoptosis by interacting with and inhibiting the p53-inducible and death-promoting BAX protein [77]. In the present study, the ratio of BAX (and its activator BID) was positive at all-time points, which indicates that the host cell tries to stop the anti-apoptotic effect of the E1B-19K protein. In fact, this non-structural adenoviral protein was identified late during Ad2 infection in a recent work using the same model [29].

### Comparison with previous functional annotation and transcription factor analyses

In our previous RNA data, we observed that most of the up-regulated genes (at 12 and 24 hpi) are involved in DNA replication, nucleic acid biosynthesis, biosynthesis of DNA, RNA and protein, or related with the cell cycle progression [9]. Most of the down-regulated genes, were involved in signal transduction, vesicle trafficking and cytoskeletal organization [9]. These results agree well with most of the current results as we observed pathways such as the *De novo purine and pyrimidine biosynthesis pathways*, which are intimately connected with the nucleic acid biosynthesis, as overrepresented. The ratios of the MCM components, DNA replication factors, and some proteins related with the cell cycle (CDK2, CDK5, CDKN2A, CDC5L) were also positive at the protein level. We also observed the inactivation of the *Integrin signalling pathway* and the negative ratios of several transport proteins (RAB3B, RAB3D), solute carrier family proteins (SLC35F6, SLC35B2, SLC4A7, SLC38A2) and syntaxins (STXBP3, STX12, STX4, STX7). However, some differences were also noted. The ratios of proteins involved in the vesicle trafficking such as SEC24D, SEC16A, SEC31A, SEC24A, SEC23IP were positive, while they were negative at the mRNA level. Moreover, the *Cytoskeletal regulation by Rho GTPase pathway* was only scored at the protein level. A previous study using RNA data from mouse embryo fibroblasts infected with Ad5, demonstrated that several pathways related with the cytoskeletal regulation (*Focal Adhesion*, *Tight Junction* and *Actin Cytoskeletal pathways*) are up-regulated [78]. In addition, different studies suggest that some of these pathways are interlinked with *Toll-Like Receptor pathway* [79, 80], a pathway also activated after infection of human A549 cells with Ad5 [78]. This pathway was not found overrepresented in the mRNA data, but it was the most overrepresented in the protein data at 12 hpi.

Among the transcription factors predicted from the RNA data, AP2, E2F1 and different ATF/CREB members were the most overrepresented, while SRF, NFKB and EGR1 were overrepresented in the down-regulated category [9]. The transcription factors E2F1 and ATF4 were also scored as activated using the protein data, and one of the members of the AP2 family (TFAP2A) was found slightly activated at late time points but with a score below the cut-off threshold (S4 Table). The same result was obtained for EGR1, which was found deactivated at 36 hpi but below the cut-off threshold. The activation state of NFKB transcription factor was not predicted, and for SRF, the prediction was the opposite compared to the value obtained from RNA data. However, SRF has been observed activated in mouse embryo fibroblasts infected by Ad5, like the NRF2 transcription factor [78]. In agreement with our observations here, that study noted the activation of other transcription factors (IRF7, REL or PPARG).

Differences in changes at the protein and mRNA levels during other viral infections have been described in previous reports. A combined transcriptomic and proteomic study of human hepatocellular carcinoma cells infected by Hepatitis C virus demonstrated that only 15 genes/proteins were common in transcriptomic (RNA-Seq) and proteomic data, and only 6 were common when proteomic data was compared with data from expression microarrays [81]. In addition, only one of the most relevant canonical pathways was found common when RNA and protein data were compared. Moreover, in a recent publication where the expression dynamics of transcripts, proteins and phosphoproteins upon VSV infection of macrophages was studied, surprising differences between the three levels were demonstrated [17].

### Phosphopeptide analysis

This study enabled us to identify 305 phosphorylated sites without performing any phosphopeptide enrichment (S2 Table). After the application of the same filtering criterions as for the protein regulation, several phosphorylated sites could be quantified at the different time points (Table 1). Among them, only a few were significantly altered, and their ratios can be found in S5 Table. These low numbers, as well as the lack of values for all time points, indicate that enrichment methodologies are needed in future studies to obtain a better view of the infection progression at the phosphoproteome level. Despite these limitations, some interesting results were found, such as the quantification of Ser21 residue in the triosephosphate isomerase (TPI), a protein involved in the *Glycolysis* pathway. This phosphorylated site had a positive ratio between 6 and 24 hpi, and it has been suggested as a substrate of the CDK2 kinase protein during etoposide-induced apoptosis in HeLa cells [82]. Another phosphorylated site found in higher concentration between 12 and 36 hpi was the Ser2152 of the filamin A (FLNA). The phosphorylation of this residue by the PAK1 protein has been pointed out as essential for the PAK1 induced cytoskeletal reorganization [83], which suggests that the activity of PAK1 is induced. Unfortunately, PAK1 was not detected in the present study. Moreover, Ser82 of the HSPB1 protein was more abundant at 6 and 24 hpi, which is one of the three phosphorylations needed for the main functions of the protein [84]. Moreover, some proteins were reported with multiple phosphorylation sites, such as Ser6 and Ser37 in CAV1 protein, or Ser39 and Ser56 in VIM protein. In the case of CAV1, both phosphorylated sites had positive ratios at the late time points, but in the case of VIM1, the ratios of Ser39 and Ser 59 were positive and negative, respectively, at 36 hpi. These results indicate that further in depth phosphoproteomics studies are necessary to clarify the whole picture of the Ad2 infection.

## Conclusions

Virus infections are a large problem in the world and we need mechanistic insights to understand how we can combat them. By using a time-resolved proteomics approach based on SILAC and mass spectrometry, we have successfully studied the early and late phases of the infection of IMR-90 cells by Ad2. The altered abundances of 2169 proteins were possible to track between 6 and 36 hpi. These findings point out the *De novo purine and pyrimidine biosynthesis*, the *Glycolysis* and the *Cytoskeletal regulation by Rho GTPase pathways* as pathways activated early during the infection, while the inactivation of the *Integrin signalling* pathway starts between 6 and 12 hpi. The predicted activation of several transcription factors such as MYC can explain the induction of the expression of glycolytic target genes, as well as the increased nucleotide biosynthesis needed for optimal adenovirus replication. This is also supported by the alteration of some phosphorylated sites related with the glycolysis or the cytoskeletal reorganization. The reported processes are important for a deeper understanding of the battle between the adenovirus and the host cell and the results clearly show that proteomic data is essential and cannot be predicted from RNA data for revealing the mechanisms.

## Supporting information

S1 Table Raw data for protein identification and quantification. (XLSX)

S2 Table Raw data for phophosite identification and quantification. (XLSX)

S3 Table **A)** In grey, expression values of proteins uniquely deregulated at 6 hours post Adenovirus type 2 infection (in log2 scale). **B)** In grey, expression values of proteins uniquely deregulated at 12 hours post Adenovirus type 2 infection (in log2 scale). **C)** In grey, expression values of proteins uniquely deregulated at 6 and 12 hours post Adenovirus type 2 infection (in log2 scale). **D)** Expression values of the differentially expressed proteins at 6, 12, 24 and 36 hours post Adenovirus type 2 infection (in log2 scale).
(PDF)

S4 Table Full list of transcription factors predicted by the upstream regulator analysis of IMR-90 cells after adenovirus type 2 infection. (XLSX)

S5 Table Differentially expressed phosphosites found in IMR-90 cells post adenovirus type 2 infection (in log2 scale). (PDF)

S1 Fig Dynamic of protein regulation in the present (black) and in a previous work [30] (gray) for (A) HSPA1A, (B) MRE11A, (C) ITGA3, (D) POLDIP3, (E) RAD50. (TIF)

S2 Fig Box-plot graph and hierarchical clustering at 6, 12, 24 and 36 hours of the proteins involved in: (A) the *Glycolysis pathway*, (B) the *FGF signalling pathway*, and (C) the *EGF receptor signalling pathway*. (TIF)

S3 Fig Log2 fold change correlation between the protein and RNA levels of those molecules used for the transcription regulator prediction: (A) MYC, (B) E2F1, (C) CREB1, (D) NFE2L2, (E) RB1, (F) SRF. (TIF)

## References

[1] Arany Z, Newsome D, Oldread E, Livingston DM, Eckner R. A family of transcriptional adaptor proteins targeted by the E1A oncoprotein. Nature. 1995;374: 81–84. 10.1038/374081a0 7870178

[2] Bagchi S, Raychaudhuri P, Nevins JR. Adenovirus E1A proteins can dissociate heteromeric complexes involving the E2F transcription factor: a novel mechanism for E1A trans-activation. Cell. 1990;62: 659–669. 2143697 10.1016/0092-8674(90)90112-r

[3] Flint J, Shenk T. Viral transactivating proteins. Annu Rev Genet. 1997;31: 177–212. 10.1146/annurev.genet.31.1.177 9442894

[4] Querido E, Blanchette P, Yan Q, Kamura T, Morrison M, Boivin D, et al Degradation of p53 by adenovirus E4orf6 and E1B55K proteins occurs via a novel mechanism involving a Cullin-containing complex. Gene Dev. 2001;15: 3104–3117. 10.1101/gad.926401 11731475 PMC312842

[5] Yew PR, Berk AJ. Inhibition of p53 transactivation required for transformation by adenovirus early 1B protein. Nature. 1992;357: 82–85. 10.1038/357082a0 1533443

[6] Wold WS, Tollefson AE, Hermiston TW. E3 transcription unit of adenovirus. Curr Top Microbiol. 1995;199: 237–274. 10.1007/978-3-642-79496-4_13 7555057

[7] Boyer J, Rohleder K, Ketner G. Adenovirus E4 34k and E4 11k inhibit double strand break repair and are physically associated with the cellular DNA-dependent protein kinase. Virology. 1999;263: 307–312. 10.1006/viro.1999.9866 10544104

[8] Zhao H, Granberg F, Pettersson U. How adenovirus strives to control cellular gene expression. Virology. 2007;363: 357–375. 10.1016/j.virol.2007.02.013 17367835

[9] Zhao H, Dahlö M, Isaksson A, Syvänen AC, Pettersson U. The transcriptome of the adenovirus infected cell. Virology. 2012;424: 115–128. 10.1016/j.virol.2011.12.006 22236370

[10] Zhao H, Chen M, Tellgren-Roth C, Pettersson U. Fluctuating expression of microRNAs in adenovirus infected cells. Virology. 2015;478: 99–111. 10.1016/j.virol.2015.01.033 25744056

[11] Zhao H, Chen M, Lind SB, Pettersson U. Distinct temporal changes in host cell lncRNA expression during the course of an adenovirus infection. Virology. 2016;492: 242–250. 10.1016/j.virol.2016.02.017 27003248 PMC7111612

[12] Yates JR, Ruse CI, Nakorchevsky A. Proteomics by Mass Spectrometry: Approaches, Advances, and Applications. Annu Rev Biomed Eng. 2009;11: 49–79. 10.1146/annurev-bioeng-061008-124934 19400705

[13] Lau E, Cao Q, Ng DC, Bleakley BJ, Dincer TU, Bot BM, et al A large dataset of protein dynamics in the mammalian heart proteome. Sci Data. 2016;3: 160015 10.1038/sdata.2016.15 26977904 PMC4792174

[14] Tan H, Yang K, Li Y, Shaw TI, Wang Y, Blanco DB, et al Integrative Proteomics and Phosphoproteomics Profiling Reveals Dynamic Signaling Networks and Bioenergetics Pathways Underlying T Cell Activation. Immunity. 2017;46: 488–503. 10.1016/j.immuni.2017.02.010 28285833 PMC5466820

[15] Valdés A, García-Cañas V, Artemenko KA, Simó C, Bergquist J, Cifuentes A. Nano–liquid chromatography–orbitrap ms–based quantitative proteomics reveals differences between the mechanisms of action of carnosic acid and carnosol in colon cancer cells. Mol Cell Proteomics. 2017;16: 8–22. 10.1074/mcp.M116.061481 27834734 PMC5217784

[16] Kulej K, Avgousti DC, Sidoli S, Herrmann C, Della Fera AN, Kim ET, et al Time-resolved Global and Chromatin Proteomics during Herpes Simplex Virus Type 1 [HSV-1] Infection. Mol Cell Proteomics. 2017;16: S92–S107. 10.1074/mcp.M116.065987 28179408 PMC5393384

[17] Kandasamy RK, Vladimer GI, Snijder B, Müller AC, Rebsamen M, Bigenzahn JW, et al A time-resolved molecular map of the macrophage response to VSV infection. Syst Biol Applications. 2016;2: 16027. 10.1038/npjsba.2016.27 PMC5516859 28725479

[18] Liu N, Song W, Wang P, Lee K, Chan W, Chen H, et al Proteomics analysis of differential expression of cellular proteins in response to avian H9N2 virus infection in human cells. Proteomics. 2008;8: 1851–1858. 10.1002/pmic.200700757 18398875

[19] McBride AA. The Promise of Proteomics in the Study of Oncogenic Viruses. Mol Cell Proteomics. 2017;16: S65–S74. 10.1074/mcp.O116.065201 28104704 PMC5393395

[20] Chahrour O, Cobice D, Malone J. Stable isotope labelling methods in mass spectrometry–based quantitative proteomics. J Pharm Biomed Anal. 2015;113: 2–20. 10.1016/j.jpba.2015.04.013 25956803

[21] Ong SE, Blagoev B, Kratchmarova I, Kristensen DB, Steen H, Pandey A, et al Stable isotope labeling by amino acids in cell culture, SILAC, as a simple and accurate approach to expression proteomics. Mol Cell Proteomics. 2002;1: 376–386. 12118079 10.1074/mcp.m200025-mcp200

[22] Gruhler A, Olsen JV, Mohammed S, Mortensen P, Færgeman NJ, Mann M, et al Quantitative Phosphoproteomics Applied to the Yeast Pheromone Signaling Pathway. Mol Cell Proteomics. 2005;4: 310–327. 10.1074/mcp.M400219-MCP200 15665377

[23] Park SK, Liao L, Kim JY, Yates JR. A Computational Approach to Correct Arginine–to–Proline Conversion in Quantitative Proteomics. Nat Methods. 2009;6: 184–185. 10.1038/nmeth0309-184 19247291 PMC2946183

[24] Park SS, Wu WW, Zhou Y, Shen RF, Martin B, Maudsley S. Effective correction of experimental errors in quantitative proteomics using stable isotope labeling by amino acids in cell culture [SILAC]. J Proteomics. 2012;75: 3720–3732. 10.1016/j.jprot.2012.04.035 22575385 PMC3394155

[25] Krämer A, Green J, Pollard J Jr, Tugendreich S. Causal analysis approaches in Ingenuity Pathway Analysis. Bioinformatics. 2014;30: 523–530. 10.1093/bioinformatics/btt703 24336805 PMC3928520

[26] Thomas PD, Campbell MJ, Kejariwal A, Mi H, Karlak B, Daverman R, et al PANTHER: a library of protein families and subfamilies indexed by function. Genome Res. 2003;13: 2129–2141. 10.1101/gr.772403 12952881 PMC403709

[27] Bosshard F, Armand F, Hamelin R, Kohn T. Mechanisms of Human Adenovirus Inactivation by Sunlight and UVC Light as Examined by Quantitative PCR and Quantitative Proteomics. Appl Environ Microbiol. 2013;79: 1325–1332. 10.1128/AEM.03457-12 23241978 PMC3568621

[28] Lam YW, Evans VC, Heesom KJ, Lamond AI, Matthews DA. Proteomics analysis of the nucleolus in adenovirus–infected cells. Mol Cell Proteomics. 2010;9: 117–130. 10.1074/mcp.M900338-MCP200 19812395 PMC2808258

[29] Källsten M, Gromova A, Zhao H, Valdés A, Konzer A, Pettersson U, et al Temporal characterization of the non–structural Adenovirus type 2 proteome and phosphoproteome using high–resolving mass spectrometry. Virology. 2017;511: 240–248. 10.1016/j.virol.2017.08.032 28915437

[30] Evans VC, Barker G, Heesom KJ, Fan J, Bessant C, Matthews DA. De novo derivation of proteomes from transcriptomes for transcript and protein identification. Nat Methods. 2012;9: 1207–1211. 10.1038/nmeth.2227 23142869 PMC3581816

[31] Zhao H, Konzer A, Mi J, Chen M, Pettersson U, Lind SB. Posttranscriptional Regulation in Adenovirus Infected Cells. J Proteome Res. 2017;16: 872–888. 10.1021/acs.jproteome.6b00834 27959563

[32] Abreu RS, Penalva LO, Marcotte EM, Vogel C. Global signatures of protein and mRNA expression levels. Mol Biosyst. 2009;5: 1512–1526. 10.1039/b908315d 20023718 PMC4089977

[33] Vogel C, Marcotte EM. Insights into the regulation of protein abundance from proteomic and transcriptomic analyses. Nat Rev Gen. 2012;13: 227–232. 10.1038/nrg3185 PMC3654667 22411467

[34] Liu Y, Beyer A, Aebersold R. On the Dependency of Cellular Protein Levels on mRNA Abundance. Cell. 2016;165: 535–550. 10.1016/j.cell.2016.03.014 27104977

[35] Shevchenko A, Chernushevich I, Wilm M, Mann M. De Novo peptide sequencing by nanoelectrospray tandem mass spectrometry using triple quadrupole and quadrupole/time–of–flight instruments. Methods Mol Biol. 2000;146: 1–16. 10.1385/1-59259-045-4:1 10948493

[36] Cox J, Mann M. MaxQuant enables high peptide identification rates, individualized p.p.b.–range mass accuracies and proteome–wide protein quantification. Nat Biotechnol. 2008;26: 1367–1372. 10.1038/nbt.1511 19029910

[37] Cox J, Neuhauser N, Michalski A, Scheltema RA, Olsen JV, Mann M. Andromeda: a peptide search engine integrated into the MaxQuant environment. J Proteome Res. 2011;10: 1794–1805. 10.1021/pr101065j 21254760

[38] Vizcaíno JA, Deutsch EW, Wang R, Csordas A, Reisinger F, Ríos D, et al ProteomeXchange provides globally coordinated proteomics data submission and dissemination. Nat Biotechnol. 2014;32: 223–226. 10.1038/nbt.2839 24727771 PMC3986813

[39] Granberg F, Svensson C, Pettersson U, Zhao H. Modulation of host cell gene expression during onset of the late phase of an adenovirus infection is focused on growth inhibition and cell architecture. Virology. 2005;343: 236–245. 10.1016/j.virol.2005.08.023 16169035

[40] Zhao H, Granberg F, Elfineh L, Pettersson U, Svensson C. Strategic attack on host cell gene expression during adenovirus infection. J Virol. 2003;77: 11006–11015. 10.1128/JVI.77.20.11006-11015.2003 14512549 PMC224976

[41] Boyd JM, Malstrom S, Subramanian T, Venkatesh LK, Schaeper U, Elangovan B, et al Adenovirus E1B 19 kDa and Bcl–2 proteins interact with a common set of cellular proteins. Cell. 1994;79: 341–351. 7954800 10.1016/0092-8674(94)90202-x

[42] Chen CC, Lau LF. Functions and Mechanisms of Action of CCN Matricellular Proteins. Int J Biochem Cell Biol. 2009;41: 771–783. 10.1016/j.biocel.2008.07.025 18775791 PMC2668982

[43] Jandova J, Beyer TE, Meuillet EJ, Watts GS. The matrix protein CCN1/CYR61 is required for αVβ5–mediated cancer cell migration. Cell Biochem Funct. 2012;30: 687–695. 10.1002/cbf.2853 22692860 PMC3468716

[44] Kireeva ML, Lam SC, Lau LF. Adhesion of human umbilical vein endothelial cells to the immediate–early gene product Cyr61 is mediated through integrin alphavbeta3. J Biol Chem. 1998;273: 3090–3096. 9446626 10.1074/jbc.273.5.3090

[45] Jun JI, Lau LF. The matricellular protein CCN1 induces fibroblast senescence and restricts fibrosis in cutaneous wound healing. Nat Cell Biol. 2010;12: 676–685. 10.1038/ncb2070 20526329 PMC2919364

[46] Wickham TJ, Mathias P, Cheresh DA, Nemerow GR. Integrins alpha v beta 3 and alpha v beta 5 promote adenovirus internalization but not virus attachment. Cell. 1993;73: 309–319. 8477447 10.1016/0092-8674(93)90231-e

[47] Nemerow GR, Stewart PL. Role of alpha(v) integrins in adenovirus cell entry and gene delivery. Microbiol Mol Biol Rev. 1999;63: 725–734. 10477314 10.1128/mmbr.63.3.725-734.1999 PMC103752

[48] Lyle C, McCormick F. Integrin αvβ5 is a primary receptor for adenovirus in CAR-negative cells. Virol J. 2010;7: 148 10.1186/1743-422X-7-148 20615244 PMC2909962

[49] Wu BJ, Hurst HC, Jones NC, Morimoto RI. The E1A 13S product of adenovirus 5 activates transcription of the cellular human HSP70 gene. Mol Cell Biol. 1986;6: 2994–2999. 3491295 10.1128/mcb.6.8.2994-2999.1986 PMC367871

[50] Shi-Wen X, Leask A, Abraham D. Regulation and function of connective tissue growth factor/CCN2 in tissue repair, scarring and fibrosis. Cytokine Growth F R. 2008;19: 133–144. 10.1016/j.cytogfr.2008.01.002 18358427

[51] Sharma V, Ichikawa M, Freeze HH. Mannose metabolism: more than meets the eye. Biochem Biophys Res Commun. 2014;453: 220–228. 10.1016/j.bbrc.2014.06.021 24931670 PMC4252654

[52] Troutman TD, Bazan JF, Pasare C. Toll–like receptors, signaling adapters and regulation of the pro–inflammatory response by PI3K. Cell Cycle. 2012;11: 3559–3567. 10.4161/cc.21572 22895011 PMC3478307

[53] Zhu J, Huang X, Yang Y. Innate immune response to adenoviral vectors is mediated by both Toll–like receptor–dependent and–independent pathways. J Virol. 2007;81: 3170–3180. 10.1128/JVI.02192-06 17229689 PMC1866082

[54] Xia Z, Sun L, Chen X, Pineda G, Jiang X, Adhikari A, et al Direct Activation of Protein Kinases by Unanchored Polyubiquitin Chains. Nature. 2009;461: 114–119. 10.1038/nature08247 19675569 PMC2747300

[55] Kawasaki T, Kawai T. Toll–like receptor signaling pathways. Front Immunol. 2014;5: 461 10.3389/fimmu.2014.00461 25309543 PMC4174766

[56] Heldin CH. Targeting the PDGF signaling pathway in tumor treatment. Cell Commun Signal. 2013;11: 97 10.1186/1478-811X-11-97 24359404 PMC3878225

[57] Saito Y, Haendeler J, Hojo Y, Yamamoto K, Berk BC. Receptor heterodimerization: essential mechanism for platelet–derived growth factor–induced epidermal growth factor receptor transactivation. Mol Cell Biol. 2001;11: 6387–6394. 10.1128/MCB.21.19.6387-6394.2001 PMC99786 11533228

[58] Sundberg C, Rubin K. Stimulation of β1 integrins on fibroblasts induces PDGF independent tyrosine phosphorylation of PDGF β–receptors. J Cell Biol. 1996;11: 741–752. 10.1083/jcb.132.4.741 PMC2199872 8647902

[59] Li E, Stupack D, Bokoch GM, Nemerow GR. Adenovirus Endocytosis Requires Actin Cytoskeleton Reorganization Mediated by Rho Family GTPases. J Virol. 1998;72: 8806–8812. 9765425 10.1128/jvi.72.11.8806-8812.1998 PMC110297

[60] Nobes CD, Hawkins P, Stephens L, Hallm A. Activation of the small GTP–binding proteins Rho and Rac by growth factor receptors. J Cell Science. 1995;108: 225–233. 7738099 10.1242/jcs.108.1.225

[61] Manser E, Leung T, Salihuddin H, Zhao Z, Lim L. A brain serine/threonine protein kinase activated by Cdc42 and Rac1. Nature. 1994;367: 40–46. 10.1038/367040a0 8107774

[62] Firat-Karalar EN, Welch MD. New mechanisms and functions of actin nucleation. Curr Opin Cell Biol. 2011;23: 4–13. 10.1016/j.ceb.2010.10.007 21093244 PMC3073586

[63] Tong X, Zhao F, Thompson CB. The molecular determinants of de novo nucleotide biosynthesis in cancer cells. Curr Opin Genet Dev. 2009;19: 32–37. 10.1016/j.gde.2009.01.002 19201187 PMC2707261

[64] de Koning TJ, Snell K, Duran M, Berger R, Poll–The BT, Surtees R. L–serine in disease and development. Biochem J. 2003;371: 653–661. 10.1042/BJ20021785 12534373 PMC1223326

[65] Lane AN, Fan TWM. Regulation of mammalian nucleotide metabolism and biosynthesis. Nucleic Acids Res. 2015;43: 2466–2485. 10.1093/nar/gkv047 25628363 PMC4344498

[66] Luo Y, Mok TS, Lin X, Zhang W, Cui Y, Guo J, et al SWATH-based proteomics identified carbonic anhydrase 2 as a potential diagnosis biomarker for nasopharyngeal carcinoma. Sci Rep 2017;7: 41191 10.1038/srep41191 28117408 PMC5259699

[67] Shen S, Guo J, Luo Y, Zhang W, Cui Y, Wang Q, et al Functional proteomics revealed IL-1β amplifies TNF downstream protein signals in human synoviocytes in a TNF-independent manner. Biochem Biophys Res Commun. 2014;450: 538–544. 10.1016/j.bbrc.2014.06.008 24928389

[68] Koussounadis A, Langdon SP, Um IH, Harrison DJ, Smith VA. Relationship between differentially expressed mRNA and mRNA-protein correlations in a xenograft model system. Sci Rep. 2015;5: 10775 10.1038/srep10775 26053859 PMC4459080

[69] Greenbaum D, Colangelo C, Williams K, Gerstein M. Comparing protein abundance and mRNA expression levels on a genomic scale. Genome Biol. 2003;4: 117 10.1186/gb-2003-4-9-117 12952525 PMC193646

[70] Löhr K, Hartmann O, Schäfer H, Dobbelstein M. Mutual interference of adenovirus infection and myc expression. J Virol. 2003;77: 7936–7944. 10.1128/JVI.77.14.7936-7944.2003 12829833 PMC161938

[71] Tworkowski KA, Chakraborty AA, Samuelson AV, Seger YR, Narita M, Hannon GJ, et al Adenovirus E1A targets p400 to induce the cellular oncoprotein Myc. Proc Natl Acad Sci USA. 2008;105: 6103–6108. 10.1073/pnas.0802095105 18413597 PMC2329713

[72] Terry LJ, Vastag L, Rabinowitz JD, Shenk T. Human kinome profiling identifies a requirement for AMP–activated protein kinase during human cytomegalovirus infection. Proc Natl Acad Sci USA. 2012;109: 3071–3076. 10.1073/pnas.1200494109 22315427 PMC3286917

[73] Thai M, Graham NA, Braas D, Nehil M, Komisopoulou E, Kurdistani SK, et al Adenovirus E4ORF1–induced MYC activation promotes host cell anabolic glucose metabolism and virus replication. Cell Metab. 2014;19: 694–701. 10.1016/j.cmet.2014.03.009 24703700 PMC4294542

[74] Zamanian M, La Thangue NB. Adenovirus E1a prevents the retinoblastoma gene product from repressing the activity of a cellular transcription factor. EMBO J. 1992;11: 2603–2610. 1385776 10.1002/j.1460-2075.1992.tb05325.x PMC556735

[75] He B. Viruses, endoplasmic reticulum stress, and interferon responses. Cell Death Differ. 2006;13: 393–403. 10.1038/sj.cdd.4401833 16397582

[76] Thompson JA, Brown JC. Role of Coatomer Protein I in Virus Replication. J Virol Antivir Res. 2012;1: 1–12. 10.4172/2324-8955.1000102 PMC3879076 24392458

[77] Han J, Sabbatini P, Perez D, Rao L, Modha D, White E. The E1B 19K protein blocks apoptosis by interacting with and inhibiting the p53–inducible and death–promoting Bax protein. Genes Dev. 1996;10: 461–477. 8600029 10.1101/gad.10.4.461

[78] Hartman ZC, Black EP, Amalfitano A. Adenoviral infection induces a multi-faceted innate cellular immune response that is mediated by the toll-like receptor pathway in A549 cells. Virology. 2007;358: 357–372. 10.1016/j.virol.2006.08.041 17027060

[79] Cetin S, Ford HR, Sysko LR, Agarwal C, Wang J, Neal MD, et al Endotoxin inhibits intestinal epithelial restitution through activation of Rho-GTPase and increased focal adhesions. J Biol Chem. 2004;279: 24592–24600. 10.1074/jbc.M313620200 15169791

[80] Zeisel MB, Druet VA, Sibilia J, Klein JP, Quesniaux V, Wachsmann D. Cross talk between MyD88 and focal adhesion kinase pathways. J Immunol. 2005;174: 7393–7397. 15905587 10.4049/jimmunol.174.11.7393

[81] Woodhouse SD, Narayan R, Latham S, Lee S, Antrobus R, Gangadharan B, et al Transcriptome sequencing, microarray, and proteomic analyses reveal cellular and metabolic impact of hepatitis C virus infection in vitro. Hepatology. 2010;52: 443–453. 10.1002/hep.23733 20683944 PMC3427885

[82] Lee WH, Choi JS, Byun MR, Koo KT, Shin S, Lee SK, et al Functional inactivation of triosephosphate isomerase through phosphorylation during etoposide-induced apoptosis in HeLa cells: potential role of Cdk2. Toxicology. 2010;278: 224–228. 10.1016/j.tox.2010.02.005 20149834

[83] Vadlamudi RK, Li F, Adam L, Nguyen D, Ohta Y, Stossel TP, et al Filamin is essential in actin cytoskeletal assembly mediated by p21-activated kinase 1. Nat Cell Biol. 2002;4: 681–690. 10.1038/ncb838 12198493

[84] Kostenko S, Moens U. Heat shock protein 27 phosphorylation: kinases, phosphatases, functions and pathology. Cell Mol Life Sci. 2009;66: 3289–3307. 10.1007/s00018-009-0086-3 19593530 PMC11115724

