# Supplementary material for: Time-resolved proteomics of adenovirus infected cells
Source: PLoS One. 2018 Sep 25;13(9):e0204522. doi: 10.1371/journal.pone.0204522 (PMC6155545; doi:10.1371/journal.pone.0204522)
Supplement: S3 Table — A) In grey, expression values of proteins uniquely deregulated at 6 hours post Adenovirus type 2 infection (in log2 scale). B) In grey, expression values of proteins uniquely deregulated at 12 hours post Adenovirus type 2 infection (in log2 scale). C) In grey, expression values of proteins uniquely deregulated at 6 and 12 hours post Adenovirus type 2 infection (in log2 scale). D) Expression values of the differentially expressed proteins at 6, 12, 24 and 36 hours post Adenovirus type 2 infection (in log2 scale). (PDF) [file pone.0204522.s003.pdf]

S3A Table. In grey, expression values of proteins uniquely deregulated at 6 hours post Adenovirus type 2 infection (in log2 scale).

Protein name	Uniprot	Symbol	6 hpi			12 hpi			24 hpi			36 hpi		
			Ad2(L)/ Mock(H)	Mock(L)/ Ad2(H)	Average	Ad2(L)/ Mock(H)	Mock(L)/ Ad2(H)	Average	Ad2(L)/ Mock(H)	Mock(L)/ Ad2(H)	Average	Ad2(L)/ Mock(H)	Mock(L)/ Ad2(H)	Average
Desmocollin-1	Q9HB00	DSC1	4.32	0.68	2.50	--	--	--	--	--	--	--	-2.62	--
UPF0587 protein C1orf123	Q9NWV4	C1orf123	1.23	1.58	1.41	1.54	--	--	1.50	--	--	1.47	--	--
G-protein-signaling modulator 1	A0A0A0MSK	GPSM1	1.83	0.62	1.22	0.64	--	--	--	--	--	--	--	--
Peptidyl-prolyl cis-trans isomerase	A0A024R3V	PPIL3	1.23	1.03	1.13	1.36	-3.31	-0.97	1.12	--	--	--	--	--
Thyroid receptor-interacting protein 6	Q15654	TRIP6	0.82	1.36	1.09	0.80	--	--	--	--	--	1.35	--	--
Peptidyl-prolyl cis-trans isomerase NIMA-interacting 1	Q13526	PIN1	0.74	1.27	1.01	1.05	--	--	1.06	0.10	0.58	0.55	0.85	0.70
Treacle protein	J3KQ96	TCOF1	0.94	1.06	1.00	0.05	--	--	--	1.91	--	--	--	--
Cytosolic Fe-S cluster assembly factor NUBP2	H3BNF0	NUBP2	0.89	1.08	0.98	1.09	--	--	0.86	--	--	--	--	--
N-acetylserotonin O-methyltransferase-like protein	O95671	ASMTL	0.98	0.98	0.98	0.38	1.11	0.74	0.30	--	--	1.39	--	--
Alpha-crystallin B chain	E9PR44	CRYAB	0.93	0.99	0.96	0.94	--	--	0.76	0.53	0.64	0.40	-0.04	0.18
Protein NDRG1	B3KU62	NDRG1	0.66	1.23	0.95	1.21	0.54	0.87	0.50	0.38	0.44	-0.08	0.00	-0.04
Copper chaperone for superoxide dismutase	J3KNF4	CCS	1.07	0.81	0.94	1.39	--	--	--	--	--	1.34	--	--
Nucleoredoxin	Q6DKJ4	NXN	1.22	0.61	0.92	1.50	0.57	1.04	--	1.21	--	--	--	--
MMS19 nucleotide excision repair protein homolog	Q96T76	MMS19	0.78	1.05	0.91	0.67	0.57	0.62	0.28	--	--	-0.07	0.23	0.08
Peflin	Q9UBV8	PEF1	0.65	1.18	0.91	1.08	--	--	1.12	--	--	1.22	--	--
Egl nine homolog 1	R4SCQ0	EGLN1	0.74	1.08	0.91	--	--	--	--	--	--	--	--	--
Arf-GAP with coiled-coil, ANK repeat and PH domain-containing protein 2	A0A087X1H	ACAP2	0.90	0.89	0.90	1.32	0.46	0.89	1.01	0.51	0.76	0.44	1.03	0.73
Dihydropteridine reductase	A0A140VKA	QDPR	0.82	0.97	0.89	1.07	--	--	1.63	--	--	--	0.86	--
Basic leucine zipper and W2 domain-containing protein 2	B5MCE7	BZW2	0.97	0.70	0.83	0.87	-0.35	0.26	0.36	0.91	0.64	0.35	0.79	0.57
BCL2/adenovirus E1B 19 kDa protein-interacting protein 2	H7C096	BNIP2	0.76	0.89	0.82	1.09	--	--	--	--	--	--	--	--
Interferon-induced protein with tetratricopeptide repeats 5	Q13325	IFIT5	0.70	0.92	0.81	1.04	0.42	0.73	--	--	--	--	1.27	--
Signal transducer and activator of transcription 2	R9QGC0	STAT2	0.90	0.65	0.78	1.14	--	--	--	0.46	--	--	--	--
WASH complex subunit FAM21A	E7ESD2	FAM21A	0.79	0.76	0.78	0.78	--	--	0.18	1.92	1.05	2.96	--	--
Kinesin-like protein	V9HW29	KIF5B	0.80	0.72	0.76	0.76	0.53	0.64	0.55	0.55	0.55	0.48	0.26	0.37
ADP-ribosylation factor-like protein 2	Q53YD8	ARL2	0.68	0.83	0.75	0.60	0.27	0.44	0.36	1.11	0.73	0.30	0.41	0.35
COP9 signalosome complex subunit 5	A0A024R7W	COPS5	0.68	0.80	0.74	0.64	0.55	0.59	0.46	0.73	0.59	0.40	0.20	0.30
Coatomer subunit zeta-2	Q9P299	COPZ2	0.75	0.69	0.72	1.11	0.49	0.80	0.76	0.47	0.62	0.93	0.19	0.56
ATP-citrate synthase	A0A024R1T9	ACLY	0.66	0.77	0.71	0.77	0.52	0.65	0.54	0.49	0.51	0.70	0.42	0.56
Mitotic spindle-associated MMXD complex subunit MIP18	H3BNV7	FAM96B	0.70	0.70	0.70	0.68	0.51	0.59	--	--	--	--	--	--
Coiled-coil domain-containing protein 93	F8W9X7	CCDC93	0.72	0.67	0.70	0.58	0.66	0.62	0.51	0.68	0.60	--	--	--
UBX domain-containing protein 1	E9PJ81	UBXN1	0.65	0.72	0.68	0.35	0.93	0.64	-1.07	--	--	--	--	--
DCC-interacting protein 13-beta	B3KMB9	APPL2	0.65	0.70	0.68	0.76	0.51	0.63	0.40	0.12	0.26	--	--	--
Twinfilin-2	Q6IBS0	TWF2	0.69	0.65	0.67	1.05	0.58	0.81	0.80	0.57	0.69	0.62	0.51	0.56
Calponin	B4DDF4	CNN2	0.63	0.69	0.66	0.35	0.61	0.48	0.41	0.25	0.33	0.53	-0.26	0.14
Beta-centractin	P42025	ACTR1B	0.72	0.59	0.65	0.67	0.21	0.44	0.53	0.46	0.49	--	--	--
Protein kinase C and casein kinase substrate in neurons protein 2	Q6FIA3	PACSIN2	0.69	0.62	0.65	0.75	0.55	0.65	0.26	0.60	0.43	0.09	0.39	0.24
Conserved oligomeric Golgi complex subunit 7	A0A0S2Z652	COG7	0.62	0.65	0.63	--	0.57	--	-0.35	0.56	0.10	0.63	0.26	0.45
Dynactin subunit 3	O75935	DCTN3	0.63	0.59	0.61	0.44	0.19	0.31	0.40	0.28	0.34	0.04	-0.08	-0.02
Coatomer subunit alpha	P53621	COPA	0.60	0.62	0.61	0.77	0.55	0.66	0.43	0.53	0.48	0.65	0.37	0.51
Protein CYR61	B4DI61	CYR61	-1.20	-1.35	-1.28	--	-1.53	--	--	--	--	--	--	--

S3B Table. In grey, expression values of proteins uniquely deregulated at 12 hours post Adenovirus type 2 infection (in log2 scale).

Protein name	Uniprot	Symbol	6 hpi			12 hpi			24 hpi			36 hpi		
			Ad2(L)/ Mock(H)	Mock(L)/ Ad2(H)	Average	Ad2(L)/ Mock(H)	Mock(L)/ Ad2(H)	Average	Ad2(L)/ Mock(H)	Mock(L)/ Ad2(H)	Average	Ad2(L)/ Mock(H)	Mock(L)/ Ad2(H)	Average
14 kDa phosphohistidine phosphatase	V9HWC4	PHPT1	--	1.58	--	**2.06**	**1.83**	**1.95**	1.79	--	--	2.05	--	--
Putative nascent polypeptide-associated complex subunit alpha-like	Q9BZK3	NACAP1	--	--	--	**2.29**	**0.61**	**1.45**	--	-0.80	--	--	--	--
Histamine N-methyltransferase	P50135	HNMT	--	1.26	--	**1.71**	**1.12**	**1.41**	--	--	--	--	--	--
Tubulin alpha chain-like 3	A6NHL2	TUBAL3	--	--	--	**1.28**	**1.41**	**1.35**	--	-1.53	--	--	--	--
Argininosuccinate synthase	A0A0S2Z3D8	ASS1	--	1.71	--	**1.57**	**1.09**	**1.33**	--	--	--	--	--	--
Delta-aminolevulinic acid dehydratase	B7Z3I9	ALAD	1.07	--	--	**1.50**	**1.08**	**1.29**	--	1.23	--	--	0.57	--
Plasminogen activator inhibitor 2	B2R7Y0	SERPINB2	--	--	--	**1.10**	**1.33**	**1.22**	--	0.54	--	--	--	--
Nicotinate phosphoribosyltransferase	Q6XQN6	NAPRT	--	--	--	**1.60**	**0.79**	**1.19**	--	0.86	--	--	--	--
Glucosamine-6-phosphate isomerase	A0A024R9X5	GNPDA2	-0.89	-0.05	-0.47	**1.18**	**1.16**	**1.17**	0.90	--	--	0.96	--	--
Anamorsin	H3BV90	CIAPIN1	--	--	--	**1.35**	**1.00**	**1.17**	--	--	--	--	0.25	--
LIM and cysteine-rich domains protein 1	Q9NZU5	LMCD1	0.27	1.12	0.70	**0.90**	**1.28**	**1.09**	-0.16	0.79	0.31	-1.06	0.86	-0.10
Alanyl-tRNA editing protein Aarsd1	C9J5N1	AARSD1	--	--	--	**1.03**	**1.10**	**1.06**	--	--	--	--	--	--
COMM domain-containing protein 6	Q7Z4G1	COMMD6	0.42	--	--	**0.92**	**1.01**	**0.97**	--	--	--	--	--	--
Endophilin-B2	A0A024R896	SH3GLB2	0.49	0.94	0.72	**0.74**	**1.19**	**0.96**	--	0.59	--	-0.96	--	--
Echinoderm microtubule-associated protein-like 2	C9JRL6	EML2	0.55	--	--	**0.96**	**0.96**	**0.96**	--	--	--	--	--	--
Transcription factor jun-B	Q5U079	JUNB	0.38	0.31	0.35	**1.07**	**0.80**	**0.94**	--	--	--	--	--	--
Osteoclast-stimulating factor 1	A8K646	OSTF1	0.85	--	--	**0.98**	**0.90**	**0.94**	--	--	--	0.15	--	--
UDP-glucose 4-epimerase	Q14376	GALE	--	--	--	**1.21**	**0.66**	**0.94**	0.81	--	--	--	--	--
Eukaryotic peptide chain release factor GTP-binding subunit ERF3B	Q8IYD1	GSPT2	--	0.88	--	**1.14**	**0.70**	**0.92**	--	--	--	1.84	--	--
Dual specificity mitogen-activated protein kinase kinase 3	C7DUW4	MAP2K3	0.55	1.08	0.81	**0.80**	**1.02**	**0.91**	0.49	0.45	0.47	0.41	0.19	0.30
Protein CutA	C9IZG4	CUTA	0.63	--	--	**0.96**	**0.86**	**0.91**	1.04	--	--	1.32	--	--
14-3-3 protein sigma	P31947	SFN	0.54	0.97	0.76	**0.84**	**0.90**	**0.87**	0.52	0.24	0.38	0.43	0.21	0.32
Basic leucine zipper and W2 domain-containing protein 1	A0A024R3Z6	BZW1	0.35	1.06	0.70	**0.84**	**0.87**	**0.85**	0.51	1.16	0.84	0.45	0.86	0.66
Protein diaphanous homolog 1	A0A1E1ERW3	DIAPH1	0.28	--	--	**0.71**	**0.98**	**0.85**	0.24	--	--	0.77	--	--
Proteasome assembly chaperone 1	B2RD51	PSMG1	--	1.07	--	**0.79**	**0.85**	**0.82**	1.26	--	--	1.47	--	--
Protein DDI1 homolog 2	Q5TDH0	DDI2	0.67	--	--	**0.80**	**0.84**	**0.82**	--	--	--	--	--	--
Protein-tyrosine-phosphatase	B4DJ12	PTPN23	0.53	0.38	0.45	**0.82**	**0.81**	**0.81**	--	--	--	1.86	--	--
STE20/SPS1-related proline-alanine-rich protein kinase	X5D7P8	STK39	--	1.00	--	**0.80**	**0.82**	**0.81**	--	--	--	--	--	--
Prefoldin subunit 4	E9PQY2	PFDN4	0.71	--	--	**0.77**	**0.84**	**0.80**	--	--	--	--	--	--
Phosphoribosyl pyrophosphate synthase-associated protein 1	A0A024R8M4	PRPSAP1	0.48	0.84	0.66	**0.85**	**0.76**	**0.80**	--	-0.92	--	--	--	--
Kinesin-like protein	B0AZS5	KIF2A	--	-0.06	--	**0.74**	**0.83**	**0.78**	1.90	0.41	1.15	1.06	0.02	0.54
Actin-related protein 2/3 complex subunit 5	O15511	ARPC5	0.52	0.80	0.66	**0.64**	**0.92**	**0.78**	0.52	0.67	0.60	0.56	0.33	0.44
Annexin	Q5T0G8	ANXA11	0.45	0.91	0.68	**0.70**	**0.85**	**0.78**	0.41	0.75	0.58	0.14	0.14	0.14
N-alpha-acetyltransferase 20	P61599	NAA20	1.18	--	--	**0.96**	**0.59**	**0.78**	--	--	--	0.56	--	--
Signal transducer and activator of transcription 3	P40763	STAT3	0.62	0.57	0.60	**0.84**	**0.69**	**0.77**	0.43	0.71	0.57	0.26	-0.06	0.10
Actin-related protein 2/3 complex subunit 2	Q53R19	ARPC2	0.54	0.93	0.74	**0.68**	**0.82**	**0.75**	0.30	0.23	0.27	0.24	0.08	0.16
Heme-binding protein 1	A0A024RAS8	HEBP1	0.47	1.06	0.76	**0.69**	**0.79**	**0.74**	0.51	0.81	0.66	0.69	0.10	0.39
Actin-related protein 2	P61160	ACTR2	0.58	0.88	0.73	**0.78**	**0.69**	**0.73**	0.58	0.64	0.61	0.60	0.30	0.45
Calcium/calmodulin-dependent protein kinase type II subunit delta	D6R938	CAMK2D	0.49	0.79	0.64	**0.67**	**0.80**	**0.73**	0.07	0.54	0.31	0.45	0.24	0.35
Tropomodulin-1	P28289	TMOD1	--	--	--	**0.74**	**0.72**	**0.73**	--	0.72	--	--	--	--
Coatomer subunit beta	P35606	COPB2	0.53	0.67	0.60	**0.77**	**0.68**	**0.72**	0.42	0.62	0.52	0.42	0.41	0.41
cAMP-dependent protein kinase type II-beta regulatory subunit	B3KY43	PRKAR2B	-0.59	0.56	-0.02	**0.75**	**0.68**	**0.72**	0.60	0.45	0.52	0.62	0.08	0.35
4-trimethylaminobutyraldehyde dehydrogenase	P49189	ALDH9A1	0.48	0.66	0.57	**0.80**	**0.63**	**0.72**	0.37	0.64	0.51	0.30	0.14	0.22
Actin-related protein 2/3 complex subunit 1A	V9HVZ6	ARPC1A	0.51	0.89	0.70	**0.69**	**0.73**	**0.71**	0.41	0.68	0.54	0.54	0.38	0.46
Plastin-3	Q53GY0	PLS3	0.58	0.85	0.72	**0.74**	**0.64**	**0.69**	0.58	0.44	0.51	0.45	0.26	0.35
Serine/threonine-protein kinase 24	B4DR80	STK24	0.49	0.53	0.51	**0.71**	**0.64**	**0.68**	0.25	0.80	0.52	0.49	0.20	0.35
Tumor susceptibility gene 101 protein	F5H442	TSG101	0.54	0.55	0.55	**0.63**	**0.73**	**0.68**	0.18	0.67	0.42	0.28	-0.11	0.08
Eukaryotic translation initiation factor 5	Q6IBU0	EIF5	0.48	0.59	0.54	**0.73**	**0.63**	**0.68**	0.45	0.73	0.59	0.54	0.41	0.48
TBC1 domain family member 15	Q8TC07	TBC1D15	--	--	--	**0.68**	**0.67**	**0.68**	--	1.11	--	--	0.80	--
Poly(ADP-ribose) glycohydrolase ARH3	Q9NX46	ADPRHL2	0.24	0.47	0.35	**0.75**	**0.60**	**0.67**	1.00	0.32	0.66	0.37	0.61	0.49
Actin-related protein 2/3 complex subunit 3	Q2LE71	ARPC3	0.56	0.89	0.72	**0.63**	**0.72**	**0.67**	0.35	0.50	0.42	0.36	0.08	0.22

Coatomer subunit zeta-1	Q53FU3	COPZ1	0.52	0.72	0.62	**0.66**	**0.65**	**0.65**	0.45	0.63	0.54	0.40	0.21	0.30
Actin-related protein 2/3 complex subunit 4	F8WCF6	ARPC4	0.58	0.89	0.74	**0.65**	**0.59**	**0.62**	0.36	0.34	0.35	0.36	-0.03	0.16
Coatomer subunit gamma-2	A0A140VK12	COPG2	0.65	0.54	0.59	**0.62**	**0.59**	**0.60**	--	0.58	--	0.34	0.66	0.50
Transcriptional repressor p66-alpha	B4E0Q6	GATAD2A	-0.60	-0.37	-0.49	**-0.60**	**-1.42**	**-1.01**	0.03	-0.97	-0.47	0.58	-0.81	-0.12
DnaJ homolog subfamily C member 1	Q96KC8	DNAJC1	0.33	-0.03	0.15	**-0.60**	**-1.47**	**-1.03**	--	--	--	--	--	--
Urokinase-type plasminogen activator	Q5PY49	PLAU	-0.05	0.57	0.26	**-1.39**	**-0.89**	**-1.14**	-1.22	--	--	--	--	--

S3C Table. In grey, expression values of proteins uniquely deregulated at 6 and 12 hours post Adenovirus type 2 infection (in log2 scale).

Protein name	Uniprot	Symbol	6 hpi			12 hpi			24 hpi			36 hpi		
			Ad2(L)/ Mock(H)	Mock(L)/ Ad2(H)	Average	Ad2(L)/ Mock(H)	Mock(L)/ Ad2(H)	Average	Ad2(L)/ Mock(H)	Mock(L)/ Ad2(H)	Average	Ad2(L)/ Mock(H)	Mock(L)/ Ad2(H)	Average
Mth938 domain-containing protein	E9PNP3	AAMDC	1.08	2.05	1.56	2.07	2.12	2.10	2.13	--	--	2.17	0.44	1.30
Tissue factor pathway inhibitor 2	Q8NE89	TFPI2	1.34	0.88	1.11	2.20	1.51	1.85	1.71	--	--	--	--	--
	B2R6L0		1.12	1.38	1.25	1.56	1.49	1.53	1.53	-0.06	0.74	--	--	--
Alcohol dehydrogenase 1B	F5HB16	ADH1B	1.12	1.21	1.16	1.91	0.89	1.40	--	--	--	--	0.34	--
Trans-3-hydroxy-L-proline dehydratase	Q96EM0	L3HYPDH	1.18	0.84	1.01	1.21	1.41	1.31	--	--	--	--	--	--
Protein-glutamate O-methyltransferase	Q9H993	ARMT1	1.26	1.24	1.25	1.59	0.99	1.29	--	0.99	--	--	0.50	--
Protein phosphatase 1 regulatory subunit 14B	Q96C90	PPP1R14B	1.30	0.99	1.14	1.35	1.19	1.27	--	--	--	--	0.57	--
NADP-dependent malic enzyme	P48163	ME1	1.21	1.15	1.18	1.37	1.09	1.23	1.11	0.54	0.82	1.27	0.13	0.70
Glutamate--cysteine ligase regulatory subunit	P48507	GCLM	0.97	1.20	1.08	1.39	0.92	1.16	1.31	0.31	0.81	1.36	0.29	0.82
Migration and invasion enhancer 1	Q9BRT3	MIEN1	0.83	1.09	0.96	1.20	1.10	1.15	--	--	--	--	--	--
ADP-ribosylation factor GTPase-activating protein 3	A0A024R4U0	ARFGAP3	1.16	0.86	1.01	1.40	0.86	1.13	0.36	--	--	--	--	--
Proline synthase co-transcribed bacterial homolog protein	D3DSW3	PROSC	0.74	1.00	0.87	0.96	1.20	1.08	--	0.90	--	--	0.93	--
Synaptic vesicle membrane protein VAT-1 homolog-like	A8K288	VAT1L	0.90	1.21	1.06	0.99	1.14	1.07	0.29	1.34	0.81	0.90	-0.07	0.42
cGMP-dependent protein kinase 1	Q13976	PRKG1	1.21	0.95	1.08	1.20	0.84	1.02	1.02	0.04	0.53	1.29	-0.06	0.62
Serine/threonine-protein kinase PAK 2	Q13177	PAK2	0.94	1.21	1.08	1.11	0.93	1.02	0.36	0.81	0.58	0.79	0.37	0.58
Protein PBDC1	Q9BVG4	PBDC1	0.72	0.88	0.80	1.15	0.87	1.01	1.17	--	--	0.74	--	--
UTP--glucose-1-phosphate uridylyltransferase	Q16851	UGP2	0.77	1.17	0.97	1.02	1.00	1.01	0.42	0.40	0.41	0.47	0.16	0.31
AMP deaminase 2	H0Y360	AMPD2	0.76	1.05	0.91	1.16	0.82	0.99	0.57	0.62	0.59	0.45	0.37	0.41
Chloride intracellular channel protein	Q6FIC5	CLIC4	0.82	1.13	0.98	1.01	0.89	0.95	0.88	0.58	0.73	0.53	0.26	0.39
Proteasome assembly chaperone 3	A0A024R806	PSMG3	0.91	0.69	0.80	1.07	0.83	0.95	--	1.53	--	--	--	--
Transgelin	Q5U0D2	TAGLN	0.73	1.40	1.06	0.64	1.25	0.95	0.57	0.91	0.74	0.48	0.56	0.52
N-terminal Xaa-Pro-Lys N-methyltransferase 1	A0A024R8E4	NTMT1	0.61	1.32	0.97	0.87	1.02	0.94	0.16	1.32	0.74	0.23	--	--
Perilipin-3	O60664	PLIN3	0.67	1.09	0.88	0.93	0.88	0.91	1.19	0.55	0.87	0.66	0.14	0.40
N(G),N(G)-dimethylarginine dimethylaminohydrolase 2	V9HW53	DDAH2	0.77	0.84	0.81	1.12	0.68	0.90	0.79	0.50	0.64	0.86	0.11	0.49
Methylthioribose-1-phosphate isomerase	Q9BV20	MRI1	0.70	1.10	0.90	1.17	0.61	0.89	--	1.65	--	1.87	--	--
Echinoderm microtubule-associated protein-like 1	F8W717	EML1	0.79	0.90	0.85	0.83	0.91	0.87	--	1.47	--	--	--	--
WD repeat-containing protein 1	Q53GN4	WDR1	0.83	1.07	0.95	0.86	0.87	0.87	0.55	0.51	0.53	0.57	0.25	0.41
Serine/threonine-protein kinase Nek9	A0A024R6D1	NEK9	1.21	0.82	1.02	1.08	0.61	0.85	--	0.97	--	--	--	--
Adenylyl cyclase-associated protein	D3DPU2	CAP1	0.71	1.13	0.92	0.77	0.90	0.83	0.23	0.33	0.28	0.19	0.28	0.23
Actin-related protein 3	A0A024RAI1	ACTR3	0.68	0.90	0.79	0.84	0.77	0.80	0.51	0.57	0.54	0.67	0.06	0.36
Dipeptidyl peptidase 9	Q86TI2	DPP9	0.67	0.98	0.83	0.93	0.68	0.80	0.54	0.72	0.63	0.71	0.45	0.58
Sorting nexin-6	A8K885	SNX6	0.70	0.84	0.77	0.92	0.66	0.79	0.40	0.53	0.47	0.19	0.08	0.14
Hsc70-interacting protein	B4E0U6	ST13	0.64	0.90	0.77	0.96	0.59	0.78	0.51	0.59	0.55	0.30	0.35	0.32
Coatomer subunit epsilon	Q53HJ6	COPE	0.71	0.68	0.69	0.90	0.65	0.77	0.34	0.36	0.35	0.51	0.13	0.32
Actin-related protein 2/3 complex subunit 1B	A4D275	ARPC1B	0.66	0.91	0.78	0.85	0.69	0.77	0.57	0.77	0.67	0.55	0.26	0.41
Kinesin light chain 1	Q7RTQ8	KNS2	0.66	0.93	0.79	0.68	0.83	0.76	0.58	0.67	0.63	0.51	0.26	0.38
Coatomer subunit beta	P53618	COPB1	0.67	0.62	0.64	0.83	0.67	0.75	0.60	0.58	0.59	0.91	0.49	0.70
Inhibitor of nuclear factor kappa-B kinase subunit beta	B4DP95	IKBKB	0.77	0.75	0.76	0.81	0.68	0.74	0.45	--	--	0.79	0.49	0.64
Adenylyl cyclase-associated protein 2	P40123	CAP2	0.73	1.07	0.90	0.64	0.82	0.73	0.36	0.39	0.37	0.53	--	--
Translational activator GCN1	E1NZA1	GCN1L1	0.71	0.67	0.69	0.74	0.72	0.73	0.46	0.52	0.49	1.01	0.32	0.66
Dynein light chain Tctex-type 3	A6NGJ0	DYNLT3	0.61	0.92	0.76	0.62	0.83	0.72	0.66	0.57	0.62	0.45	--	--
Heat shock protein beta-1	V9HW43	HSPB1	0.79	1.02	0.90	0.72	0.71	0.71	0.35	0.29	0.32	0.01	0.04	0.02
Glycogen synthase kinase-3 beta	Q6FI27	GSK3B	0.79	0.65	0.72	0.83	0.59	0.71	--	0.20	--	--	--	--
Moesin	V9HWC0	MSN	0.64	0.79	0.71	0.82	0.60	0.71	0.68	0.50	0.59	0.29	0.37	0.33
Niban-like protein 1	A0A024R872	C9orf88	0.63	0.97	0.80	0.65	0.74	0.70	0.29	0.45	0.37	0.48	0.29	0.39
Microtubule-associated protein 1A	E9PGC8	MAP1A	0.75	0.60	0.68	0.62	0.70	0.66	0.55	-0.19	0.18	-0.22	-0.65	-0.44
Translation initiation factor eIF-2B subunit alpha	Q14232	EIF2B1	0.74	0.81	0.77	0.63	0.67	0.65	0.58	0.74	0.66	0.72	0.58	0.65
Collagen alpha-1(III) chain	P02461	COL3A1	-0.86	-1.72	-1.29	-0.71	-1.66	-1.18	-1.25	-0.42	-0.83	-0.51	-1.31	-0.91

S3D Table. Ratios of the differentially altered proteins at all time points post Adenovirus type 2 infection (in log2 scale).

Protein name	Uniprot	Symbol	6 hpi			12 hpi			24 hpi			36 hpi		
			Ad2(L)/ Mock(H)	Mock(L)/ Ad2(H)	Average	Ad2(L)/ Mock(H)	Mock(L)/ Ad2(H)	Average	Ad2(L)/ Mock(H)	Mock(L)/ Ad2(H)	Average	Ad2(L)/ Mock(H)	Mock(L)/ Ad2(H)	Average
Desmocollin-1	Q9HB00	DSC1	4.32	0.68	**2.50**	--	--	--	--	--	--	--	--	--
UPF0587 protein C1orf123	Q9NWV4	C1orf123	1.23	1.58	**1.41**	--	--	--	--	--	--	--	--	--
G-protein-signaling modulator 1	A0A0A0MSK4	GPSM1	1.83	0.62	**1.22**	--	--	--	--	--	--	--	--	--
Peptidyl-prolyl cis-trans isomerase	A0A024R3V4	PPIL3	1.23	1.03	**1.13**	--	--	--	--	--	--	--	--	--
Thyroid receptor-interacting protein 6	Q15654	TRIP6	0.82	1.36	**1.09**	--	--	--	--	--	--	--	--	--
Peptidyl-prolyl cis-trans isomerase NIMA-interacting 1	Q13526	PIN1	0.74	1.27	**1.01**	--	--	--	--	--	--	--	--	--
Treacle protein	J3KQ96	TCOF1	0.94	1.06	**1.00**	--	--	--	--	--	--	--	--	--
Cytosolic Fe-S cluster assembly factor NUBP2	H3BNF0	NUBP2	0.89	1.08	**0.98**	--	--	--	--	--	--	--	--	--
N-acetylserotonin O-methyltransferase-like protein	O95671	ASMTL	0.98	0.98	**0.98**	--	--	--	--	--	--	--	--	--
Alpha-crystallin B chain	E9PR44	CRYAB	0.93	0.99	**0.96**	--	--	--	--	--	--	--	--	--
Protein NDRG1	B3KU62	TRG14	0.66	1.23	**0.95**	--	--	--	--	--	--	--	--	--
Copper chaperone for superoxide dismutase	J3KNF4	CCS	1.07	0.81	**0.94**	--	--	--	--	--	--	--	--	--
Nucleoredoxin	Q6DKJ4	NXN	1.22	0.61	**0.92**	--	--	--	--	--	--	--	--	--
MMS19 nucleotide excision repair protein homolog	Q96T76	MMS19	0.78	1.05	**0.91**	--	--	--	--	--	--	--	--	--
Peflin	Q9UBV8	PEF1	0.65	1.18	**0.91**	--	--	--	--	--	--	--	--	--
Egl nine homolog 1	R4SCQ0	EGLN1	0.74	1.08	**0.91**	--	--	--	--	--	--	--	--	--
Arf-GAP with coiled-coil, ANK repeat and PH domain-containing protein 2	A0A087X1H5	ACAP2	0.90	0.89	**0.90**	--	--	--	--	--	--	--	--	--
Dihydropteridine reductase	A0A140VKA9	QDPR	0.82	0.97	**0.89**	--	--	--	--	--	--	--	--	--
Basic leucine zipper and W2 domain-containing protein 2	B5MCE7	BZW2	0.97	0.70	**0.83**	--	--	--	--	--	--	--	--	--
BCL2/adenovirus E1B 19 kDa protein-interacting protein 2	H7C096	BNIP2	0.76	0.89	**0.82**	--	--	--	--	--	--	--	--	--
Interferon-induced protein with tetratricopeptide repeats 5	Q13325	IFIT5	0.70	0.92	**0.81**	--	--	--	--	--	--	--	--	--
Signal transducer and activator of transcription	R9QGC0	STAT2	0.90	0.65	**0.78**	--	--	--	--	--	--	--	--	--
WASH complex subunit FAM21C	E7ESD2	FAM21A	0.79	0.76	**0.78**	--	--	--	--	--	--	--	--	--
Kinesin-like protein	V9HW29	KIF5B	0.80	0.72	**0.76**	--	--	--	--	--	--	--	--	--
ADP-ribosylation factor-like protein 2	Q53YD8	hCG_2337	0.68	0.83	**0.75**	--	--	--	--	--	--	--	--	--
COP9 signalosome complex subunit 5	A0A024R7W9	COPS5	0.68	0.80	**0.74**	--	--	--	--	--	--	--	--	--
Coatomer subunit zeta-2	Q9P299	COPZ2	0.75	0.69	**0.72**	--	--	--	--	--	--	--	--	--
ATP-citrate synthase	A0A024R1T9	ACLY	0.66	0.77	**0.71**	--	--	--	--	--	--	--	--	--
Mitotic spindle-associated MMXD complex subunit MIP18	H3BNV7	FAM96B	0.70	0.70	**0.70**	--	--	--	--	--	--	--	--	--
Coiled-coil domain-containing protein 93	F8W9X7	CCDC93	0.72	0.67	**0.70**	--	--	--	--	--	--	--	--	--
UBX domain-containing protein 1	E9PJ81	UBXN1	0.65	0.72	**0.68**	--	--	--	--	--	--	--	--	--
DCC-interacting protein 13-beta	B3KMB9	APPL2	0.65	0.70	**0.68**	--	--	--	--	--	--	--	--	--
Twinfilin-2	Q6IBS0	TWF2	0.69	0.65	**0.67**	--	--	--	--	--	--	--	--	--
Calponin	B4DDF4	CNN2	0.63	0.69	**0.66**	--	--	--	--	--	--	--	--	--
Beta-centractin	P42025	ACTR1B	0.72	0.59	**0.65**	--	--	--	--	--	--	--	--	--
Protein kinase C and casein kinase substrate in neurons protein 2	Q6FIA3	PACSIN2	0.69	0.62	**0.65**	--	--	--	--	--	--	--	--	--
Conserved oligomeric Golgi complex subunit 7	A0A0S2Z652	COG7	0.62	0.65	**0.63**	--	--	--	--	--	--	--	--	--
Dynactin subunit 3	O75935	DCTN3	0.63	0.59	**0.61**	--	--	--	--	--	--	--	--	--
Coatomer subunit alpha	P53621	COPA	0.60	0.62	**0.61**	--	--	--	--	--	--	--	--	--
Protein CYR61	B4DI61	CYR61	-1.20	-1.35	**-1.28**	--	--	--	--	--	--	--	--	--
14 kDa phosphohistidine phosphatase	V9HWC4	PHPT1	--	--	--	2.06	1.83	**1.95**	--	--	--	--	--	--
Putative nascent polypeptide-associated complex subunit alpha-like	Q9BZK3	NACAP1	--	--	--	2.29	0.61	**1.45**	--	--	--	--	--	--
Histamine N-methyltransferase	P50135	HNMT	--	--	--	1.71	1.12	**1.41**	--	--	--	--	--	--
Tubulin alpha chain-like 3	A6NHL2	TUBAL3	--	--	--	1.28	1.41	**1.35**	--	--	--	--	--	--
Argininosuccinate synthase	A0A0S2Z3D8	ASS1	--	--	--	1.57	1.09	**1.33**	--	--	--	--	--	--
Delta-aminolevulinic acid dehydratase	B7Z3I9	ALAD	--	--	--	1.50	1.08	**1.29**	--	--	--	--	--	--
Plasminogen activator inhibitor 2	B2R7Y0	SERPINB2	--	--	--	1.10	1.33	**1.22**	--	--	--	--	--	--
Nicotinate phosphoribosyltransferase	Q6XQN6	NAPRT	--	--	--	1.60	0.79	**1.19**	--	--	--	--	--	--

S3D Table. *Cont.*

Protein name	Uniprot	Symbol	6 hpi			12 hpi			24 hpi			36 hpi		
			Ad2(L)/ Mock(H)	Mock(L)/ Ad2(H)	Average	Ad2(L)/ Mock(H)	Mock(L)/ Ad2(H)	Average	Ad2(L)/ Mock(H)	Mock(L)/ Ad2(H)	Average	Ad2(L)/ Mock(H)	Mock(L)/ Ad2(H)	Average
Glucosamine-6-phosphate isomerase	A0A024R9X5	GNPDA2	--	--	--	1.18	1.16	**1.17**	--	--	--	--	--	--
Anamorsin	H3BV90	CIAPIN1	--	--	--	1.35	1.00	**1.17**	--	--	--	--	--	--
LIM and cysteine-rich domains protein 1	Q9NZU5	LMCD1	--	--	--	0.90	1.28	**1.09**	--	--	--	--	--	--
Alanyl-tRNA editing protein Aarsd1	C9J5N1	AARSD1	--	--	--	1.03	1.10	**1.06**	--	--	--	--	--	--
COMM domain-containing protein 6	Q7Z4G1	COMMD6	--	--	--	0.92	1.01	**0.97**	--	--	--	--	--	--
Endophilin-B2	A0A024R896	SH3GLB2	--	--	--	0.74	1.19	**0.96**	--	--	--	--	--	--
Echinoderm microtubule-associated protein-like 2	C9JRL6	EML2	--	--	--	0.96	0.96	**0.96**	--	--	--	--	--	--
Transcription factor jun-B	Q5U079	JUNB	--	--	--	1.07	0.80	**0.94**	--	--	--	--	--	--
Osteoclast-stimulating factor 1	A8K646	OSTF1	--	--	--	0.98	0.90	**0.94**	--	--	--	--	--	--
UDP-glucose 4-epimerase	Q14376	GALE	--	--	--	1.21	0.66	**0.94**	--	--	--	--	--	--
Eukaryotic peptide chain release factor GTP-binding subunit ERF3B	Q8IYD1	GSPT2	--	--	--	1.14	0.70	**0.92**	--	--	--	--	--	--
Dual specificity mitogen-activated protein kinase kinase 3	C7DUW4	MAP2K3	--	--	--	0.80	1.02	**0.91**	--	--	--	--	--	--
Protein CutA	C9IZG4	CUTA	--	--	--	0.96	0.86	**0.91**	--	--	--	--	--	--
14-3-3 protein sigma	P31947	SFN	--	--	--	0.84	0.90	**0.87**	--	--	--	--	--	--
Basic leucine zipper and W2 domain-containing protein 1	A0A024R3Z6	BZW1	--	--	--	0.84	0.87	**0.85**	--	--	--	--	--	--
Protein diaphanous homolog 1	A0A1E1ERW3	DIAPH1	--	--	--	0.71	0.98	**0.85**	--	--	--	--	--	--
Proteasome assembly chaperone 1	B2RD51	PSMG1	--	--	--	0.79	0.85	**0.82**	--	--	--	--	--	--
Protein DDI1 homolog 2	Q5TDH0	DDI2	--	--	--	0.80	0.84	**0.82**	--	--	--	--	--	--
Protein-tyrosine-phosphatase	B4DJ12	PTPN23	--	--	--	0.82	0.81	**0.81**	--	--	--	--	--	--
STE20/SPS1-related proline-alanine-rich protein kinase	X5D7P8	STK39	--	--	--	0.80	0.82	**0.81**	--	--	--	--	--	--
Prefoldin subunit 4	E9PQY2	PFDN4	--	--	--	0.77	0.84	**0.80**	--	--	--	--	--	--
Phosphoribosyl pyrophosphate synthase-associated protein 1	A0A024R8M4	PRPSAP1	--	--	--	0.85	0.76	**0.80**	--	--	--	--	--	--
Kinesin-like protein	B0AZS5	KIF2A	--	--	--	0.74	0.83	**0.78**	--	--	--	--	--	--
Actin-related protein 2/3 complex subunit 5	O15511	ARPC5	--	--	--	0.64	0.92	**0.78**	--	--	--	--	--	--
Annexin	Q5T0G8	ANXA11	--	--	--	0.70	0.85	**0.78**	--	--	--	--	--	--
N-alpha-acetyltransferase 20	P61599	NAA20	--	--	--	0.96	0.59	**0.78**	--	--	--	--	--	--
Signal transducer and activator of transcription 3	P40763	STAT3	--	--	--	0.84	0.69	**0.77**	--	--	--	--	--	--
Actin-related protein 2/3 complex subunit 2	Q53R19	ARPC2	--	--	--	0.68	0.82	**0.75**	--	--	--	--	--	--
Heme-binding protein 1	A0A024RAS8	HEBP1	--	--	--	0.69	0.79	**0.74**	--	--	--	--	--	--
Actin-related protein 2	P61160	ACTR2	--	--	--	0.78	0.69	**0.73**	--	--	--	--	--	--
Calcium/calmodulin-dependent protein kinase type II subunit delta	D6R938	CAMK2D	--	--	--	0.67	0.80	**0.73**	--	--	--	--	--	--
Tropomodulin-1	P28289	TMOD1	--	--	--	0.74	0.72	**0.73**	--	--	--	--	--	--
Coatomer subunit beta	P35606	COPB2	--	--	--	0.77	0.68	**0.72**	--	--	--	--	--	--
cAMP-dependent protein kinase type II-beta regulatory subunit	B3KY43	PRKAR2B	--	--	--	0.75	0.68	**0.72**	--	--	--	--	--	--
4-trimethylaminobutyraldehyde dehydrogenase	P49189	ALDH9A1	--	--	--	0.80	0.63	**0.72**	--	--	--	--	--	--
Actin-related protein 2/3 complex subunit 1A	V9HVZ6	ARPC1A	--	--	--	0.69	0.73	**0.71**	--	--	--	--	--	--
Plastin-3	Q53GY0	PLS3	--	--	--	0.74	0.64	**0.69**	--	--	--	--	--	--
Serine/threonine-protein kinase 24	B4DR80	STK24	--	--	--	0.71	0.64	**0.68**	--	--	--	--	--	--
Tumor susceptibility gene 101 protein	F5H442	TSG101	--	--	--	0.63	0.73	**0.68**	--	--	--	--	--	--
Eukaryotic translation initiation factor 5	Q6IBU0	EIF5	--	--	--	0.73	0.63	**0.68**	--	--	--	--	--	--
TBC1 domain family member 15	Q8TC07	TBC1D15	--	--	--	0.68	0.67	**0.68**	--	--	--	--	--	--
Poly(ADP-ribose) glycohydrolase ARH3	Q9NX46	ADPRHL2	--	--	--	0.75	0.60	**0.67**	--	--	--	--	--	--
Actin-related protein 2/3 complex subunit 3	Q2LE71	ARPC3	--	--	--	0.63	0.72	**0.67**	--	--	--	--	--	--
Coatomer subunit zeta-1	Q53FU3	COPZ1	--	--	--	0.66	0.65	**0.65**	--	--	--	--	--	--
Actin-related protein 2/3 complex subunit 4	F8WCF6	ARPC4	--	--	--	0.65	0.59	**0.62**	--	--	--	--	--	--
Coatomer subunit gamma-2	A0A140VK12	COPG2	--	--	--	0.62	0.59	**0.60**	--	--	--	--	--	--
Transcriptional repressor p66-alpha	B4E0Q6	GATAD2A	--	--	--	-0.60	-1.42	**-1.01**	--	--	--	--	--	--
DnaJ homolog subfamily C member 1	Q96KC8	DNAJC1	--	--	--	-0.60	-1.47	**-1.03**	--	--	--	--	--	--

***S3D Table**. Cont.*

Protein name	Uniprot	Symbol	6 hpi			12 hpi			24 hpi			36 hpi		
			Ad2(L)/ Mock(H)	Mock(L)/ Ad2(H)	Average	Ad2(L)/ Mock(H)	Mock(L)/ Ad2(H)	Average	Ad2(L)/ Mock(H)	Mock(L)/ Ad2(H)	Average	Ad2(L)/ Mock(H)	Mock(L)/ Ad2(H)	Average
Urokinase-type plasminogen activator	Q5PY49	PLAU	--	--	--	-1.39	-0.89	**-1.14**	--	--	--	--	--	--
Transcription factor BTF3	D6RDG3	BTF3	--	--	--	--	--	--	3.32	3.53	**3.42**	--	--	--
Probable RNA-binding protein EIF1AD	E9PQD0	EIF1AD	--	--	--	--	--	--	2.89	2.91	**2.90**	--	--	--
PCNA-associated factor	H0YMA4	KIAA0101	--	--	--	--	--	--	2.29	2.36	**2.33**	--	--	--
CLIP-associating protein 1	F8WA11	CLASP1	--	--	--	--	--	--	1.97	2.00	**1.98**	--	--	--
Protein PRRC2C	E7EPN9	PRRC2C	--	--	--	--	--	--	2.12	1.81	**1.96**	--	--	--
E3 ubiquitin-protein ligase TRIM4	H7C0Q6	TRIM4	--	--	--	--	--	--	0.83	2.99	**1.91**	--	--	--
La-related protein 4	X6RLN4	LARP4	--	--	--	--	--	--	1.86	1.89	**1.87**	--	--	--
Glycogenin-1	Q8N5Y3	GYG1	--	--	--	--	--	--	1.71	1.97	**1.84**	--	--	--
Translation machinery-associated protein 7	A0A024R306	CCDC72	--	--	--	--	--	--	1.65	1.92	**1.78**	--	--	--
Ataxin-2-like protein	Q8WWM7	ATXN2L	--	--	--	--	--	--	1.23	2.30	**1.76**	--	--	--
Zinc finger CCCH-type antiviral protein 1	A8K9U6	ZC3HAV1	--	--	--	--	--	--	1.84	1.64	**1.74**	--	--	--
Spartin	A8K6Q9	SPG20	--	--	--	--	--	--	1.55	1.89	**1.72**	--	--	--
Caspase	G3V169	CASP1	--	--	--	--	--	--	1.55	1.65	**1.60**	--	--	--
Ubiquitin-conjugating enzyme E2 D1	A0A024QZJ2	UBE2D1	--	--	--	--	--	--	1.70	1.47	**1.58**	--	--	--
Protein Hikeshi	Q53FT3	C11orf73	--	--	--	--	--	--	1.58	1.54	**1.56**	--	--	--
DnaJ homolog subfamily C member 9	A0A024QZN2	DNAJC9	--	--	--	--	--	--	1.41	1.53	**1.47**	--	--	--
Eukaryotic translation initiation factor 4 gamma 2	P78344	EIF4G2	--	--	--	--	--	--	1.91	1.00	**1.45**	--	--	--
Calpastatin	E7ES10	CAST	--	--	--	--	--	--	1.22	1.52	**1.37**	--	--	--
Protein FRG1	E9PRR7	FRG1	--	--	--	--	--	--	1.48	1.24	**1.36**	--	--	--
TBC1 domain family member 13	A0A024R8C8	TBC1D13	--	--	--	--	--	--	1.68	0.97	**1.32**	--	--	--
Tetratricopeptide repeat protein 1	Q53HD9	TTC1	--	--	--	--	--	--	1.46	1.15	**1.31**	--	--	--
U4/U6.U5 tri-snRNP-associated protein 1	O43290	SART1	--	--	--	--	--	--	1.18	1.42	**1.30**	--	--	--
Transcription elongation factor SPT5	B4E0Q4	SUPT5H	--	--	--	--	--	--	0.87	1.73	**1.30**	--	--	--
Putative RNA-binding protein Luc7-like 1	Q1W6G4	LUC7L	--	--	--	--	--	--	1.39	1.13	**1.26**	--	--	--
Charged multivesicular body protein 1b	B2RA72	CHMP1B	--	--	--	--	--	--	1.41	1.04	**1.22**	--	--	--
General transcription factor IIF subunit 1	Q6IBK5	GTF2F1	--	--	--	--	--	--	1.31	1.13	**1.22**	--	--	--
Pumilio homolog 1	H0YEH2	PUM1	--	--	--	--	--	--	1.49	0.95	**1.22**	--	--	--
WW domain-binding protein 11	B4DMD3	WBP11	--	--	--	--	--	--	1.02	1.37	**1.20**	--	--	--
La-related protein 4B	Q92615	LARP4B	--	--	--	--	--	--	0.99	1.40	**1.20**	--	--	--
NEDD8-activating enzyme E1 regulatory subunit	Q13564	NAE1	--	--	--	--	--	--	1.14	1.24	**1.19**	--	--	--
5-nucleotidase domain-containing protein 1	A8K2Z3	NT5DC1	--	--	--	--	--	--	0.86	1.51	**1.19**	--	--	--
Myeloid differentiation primary response protein MyD88	A0A0A0MS70	MYD88	--	--	--	--	--	--	1.18	1.17	**1.18**	--	--	--
RAC-alpha serine/threonine-protein kinase	B3KVH4	AKT1	--	--	--	--	--	--	1.07	1.23	**1.15**	--	--	--
Perilipin	Q6FHZ7	ADFP	--	--	--	--	--	--	1.70	0.60	**1.15**	--	--	--
Ataxin-2	H0YH87	ATXN2	--	--	--	--	--	--	1.00	1.26	**1.13**	--	--	--
Acyl-protein thioesterase 2	A0A140VJC9	LYPLA2	--	--	--	--	--	--	1.22	1.02	**1.12**	--	--	--
Ubiquitin-like protein 4A	P11441	UBL4A	--	--	--	--	--	--	1.27	0.93	**1.10**	--	--	--
Ubiquilin-2	B4DZF1	UBQLN2	--	--	--	--	--	--	1.17	1.00	**1.08**	--	--	--
S-phase kinase-associated protein 1	P63208	SKP1	--	--	--	--	--	--	0.88	1.29	**1.08**	--	--	--
Glutamine-dependent NAD(+) synthetase	A0A0B4J216	NADSYN1	--	--	--	--	--	--	1.25	0.89	**1.07**	--	--	--
Protein-methionine sulfoxide oxidase MICAL1	Q8TDZ2	MICAL1	--	--	--	--	--	--	1.25	0.90	**1.07**	--	--	--
N-alpha-acetyltransferase 25, NatB auxiliary subunit	A8K8X0	NAA25	--	--	--	--	--	--	0.99	1.11	**1.05**	--	--	--
CD2-associated protein	Q9Y5K6	CD2AP	--	--	--	--	--	--	1.35	0.74	**1.05**	--	--	--
Acyl-protein thioesterase 1	A0A087X1K9	LYPLA1	--	--	--	--	--	--	1.37	0.71	**1.04**	--	--	--
Telomeric repeat-binding factor 2-interacting protein 1	Q9NYB0	TERF2IP	--	--	--	--	--	--	1.10	0.97	**1.03**	--	--	--
DCN1-like protein	B4DM76	DCUN1D1	--	--	--	--	--	--	1.17	0.86	**1.02**	--	--	--
MAP7 domain-containing protein 1	D3DPS3	RPRC1	--	--	--	--	--	--	1.00	0.99	**1.00**	--	--	--

***S3D Table**. Cont.*

Protein name	Uniprot	Symbol	6 hpi			12 hpi			24 hpi			36 hpi		
			Ad2(L)/ Mock(H)	Mock(L)/ Ad2(H)	Average	Ad2(L)/ Mock(H)	Mock(L)/ Ad2(H)	Average	Ad2(L)/ Mock(H)	Mock(L)/ Ad2(H)	Average	Ad2(L)/ Mock(H)	Mock(L)/ Ad2(H)	Average
Cilia- and flagella-associated protein 20	Q9Y6A4	CFAP20	--	--	--	--	--	--	0.84	1.13	**0.99**	--	--	--
Nucleolar protein 10	Q9BSC4	NOL10	--	--	--	--	--	--	0.96	1.00	**0.98**	--	--	--
Proteasome inhibitor PI31 subunit	Q5QPM7	PSMF1	--	--	--	--	--	--	1.17	0.79	**0.98**	--	--	--
Paxillin	F5GZ78	PXN	--	--	--	--	--	--	0.91	0.99	**0.95**	--	--	--
COP9 signalosome complex subunit 3	Q9UNS2	COPS3	--	--	--	--	--	--	0.97	0.90	**0.94**	--	--	--
Eukaryotic translation initiation factor 3 subunit J	A0A024R5S5	EIF3S1	--	--	--	--	--	--	1.03	0.84	**0.93**	--	--	--
Nitric oxide synthase-interacting protein	A8K670	NOSIP	--	--	--	--	--	--	0.95	0.90	**0.92**	--	--	--
Serine/arginine-rich splicing factor 11	Q05BU6	SFRS11	--	--	--	--	--	--	0.89	0.96	**0.92**	--	--	--
Methionine aminopeptidase 2	A0A140VJE3	METAP2	--	--	--	--	--	--	1.08	0.76	**0.92**	--	--	--
Cdc42-interacting protein 4	W4VSQ9	TRIP10	--	--	--	--	--	--	0.97	0.86	**0.92**	--	--	--
Eukaryotic initiation factor 4A-II	Q14240	EIF4A2	--	--	--	--	--	--	1.02	0.82	**0.92**	--	--	--
Replication factor C subunit 2	Q75MT5	RFC2	--	--	--	--	--	--	1.15	0.68	**0.91**	--	--	--
Lupus La protein	B5BUB5	SSB	--	--	--	--	--	--	0.74	1.08	**0.91**	--	--	--
Crk-like protein	P46109	CRKL	--	--	--	--	--	--	0.76	1.03	**0.89**	--	--	--
Probable ATP-dependent RNA helicase DDX56	H7C3E9	DDX56	--	--	--	--	--	--	0.78	0.99	**0.89**	--	--	--
Uncharacterized protein FLJ45252	A0A096LP25	AAK1	--	--	--	--	--	--	0.67	1.09	**0.88**	--	--	--
Heat shock-related 70 kDa protein 2	A0A024R6B5	HSPA2	--	--	--	--	--	--	0.79	0.93	**0.86**	--	--	--
Mitochondrial antiviral-signaling protein	Q7Z434	MAVS	--	--	--	--	--	--	0.81	0.90	**0.86**	--	--	--
	B2RE29	NUDT4	--	--	--	--	--	--	0.82	0.89	**0.86**	--	--	--
Radixin	B0YJ88	RDX	--	--	--	--	--	--	0.78	0.92	**0.85**	--	--	--
Pre-mRNA-splicing factor ISY1 homolog	Q9ULR0	ISY1	--	--	--	--	--	--	0.60	1.10	**0.85**	--	--	--
Zinc finger CCCH domain-containing protein 18	E7ERS3	ZC3H18	--	--	--	--	--	--	1.01	0.69	**0.85**	--	--	--
Ran GTPase-activating protein 1	A0A024R1U0	RANGAP1	--	--	--	--	--	--	0.78	0.89	**0.84**	--	--	--
Protein LYRIC	A0A024R9D2	MTDH	--	--	--	--	--	--	0.61	1.05	**0.83**	--	--	--
Protein transport protein Sec24A	B4E205	SEC24A	--	--	--	--	--	--	0.67	0.97	**0.82**	--	--	--
Stathmin	E5RGX5	STMN2	--	--	--	--	--	--	0.86	0.77	**0.82**	--	--	--
Probable aminopeptidase NPEPL1	H0UI76	NPEPL1	--	--	--	--	--	--	0.83	0.80	**0.81**	--	--	--
Protein DEK	P35659	DEK	--	--	--	--	--	--	0.84	0.79	**0.81**	--	--	--
PDZ and LIM domain protein 3	A0A087WYF8	PDLIM3	--	--	--	--	--	--	0.61	1.02	**0.81**	--	--	--
Exportin-7	A8K607	XPO7	--	--	--	--	--	--	0.84	0.79	**0.81**	--	--	--
5-AMP-activated protein kinase subunit gamma-1	F8VYY9	PRKAG1	--	--	--	--	--	--	0.86	0.74	**0.80**	--	--	--
Transcription elongation factor A protein 1	P23193	TCEA1	--	--	--	--	--	--	0.69	0.89	**0.79**	--	--	--
KIF1-binding protein	A0A1B0GUA3	KIF1BP	--	--	--	--	--	--	0.78	0.79	**0.78**	--	--	--
Ubiquitin-like domain-containing CTD phosphatase 1	Q8WVY7	UBLCP1	--	--	--	--	--	--	0.84	0.71	**0.78**	--	--	--
Cysteine-rich protein 2	Q53FN1	CRIP2	--	--	--	--	--	--	0.94	0.61	**0.77**	--	--	--
Putative ATP-dependent RNA helicase DHX30	H7BXY3	DHX30	--	--	--	--	--	--	0.65	0.89	**0.77**	--	--	--
Eukaryotic translation initiation factor 1A, X-chromosomal	P47813	EIF1AX	--	--	--	--	--	--	0.59	0.93	**0.76**	--	--	--
Actin-related protein 2/3 complex subunit 5	B3KPC7	ARPC5L	--	--	--	--	--	--	0.69	0.82	**0.75**	--	--	--
COP9 signalosome complex subunit 8	E9PGT6	COPS8	--	--	--	--	--	--	0.77	0.72	**0.75**	--	--	--
Pleiotropic regulator 1	O43660	PLRG1	--	--	--	--	--	--	0.72	0.78	**0.75**	--	--	--
40S ribosomal protein S23	A8K517	RPS23	--	--	--	--	--	--	0.65	0.84	**0.75**	--	--	--
Mortality factor 4-like protein 1	H0YLJ3	MORF4L1	--	--	--	--	--	--	0.80	0.66	**0.73**	--	--	--
Regulator of G-protein signaling 10	O43665	RGS10	--	--	--	--	--	--	0.82	0.64	**0.73**	--	--	--
Ubiquilin-1	B3KNI2	UBQLN1	--	--	--	--	--	--	0.67	0.79	**0.73**	--	--	--
Serine/threonine-protein phosphatase	Q8WZ56	PPP2CB	--	--	--	--	--	--	0.82	0.63	**0.73**	--	--	--
40S ribosomal protein SA	C9J9K3	RPSA	--	--	--	--	--	--	0.65	0.80	**0.72**	--	--	--
Ubiquitin-40S ribosomal protein S27a	Q5RKT7	RPS27A	--	--	--	--	--	--	0.67	0.76	**0.72**	--	--	--
60S ribosomal protein L4	Q59GY2	RPL4	--	--	--	--	--	--	0.66	0.77	**0.72**	--	--	--

S3D Table. *Cont.*

Protein name	Uniprot	Symbol	6 hpi			12 hpi			24 hpi			36 hpi		
			Ad2(L)/ Mock(H)	Mock(L)/ Ad2(H)	Average	Ad2(L)/ Mock(H)	Mock(L)/ Ad2(H)	Average	Ad2(L)/ Mock(H)	Mock(L)/ Ad2(H)	Average	Ad2(L)/ Mock(H)	Mock(L)/ Ad2(H)	Average
WD repeat-containing protein 82	A0A024R333	TMEM113	--	--	--	--	--	--	0.71	0.72	**0.71**	--	--	--
COP9 signalosome complex subunit 4	B3KM48	COPS4	--	--	--	--	--	--	0.65	0.77	**0.71**	--	--	--
Poly(rC)-binding protein 1	Q53SS8	PCBP1	--	--	--	--	--	--	0.67	0.74	**0.71**	--	--	--
BRISC and BRCA1-A complex member 1	M0R0I0	BABAM1	--	--	--	--	--	--	0.69	0.72	**0.71**	--	--	--
Peptidyl-prolyl cis-trans isomerase	A0A024RCX8	PPIL1	--	--	--	--	--	--	0.68	0.73	**0.70**	--	--	--
Transaldolase	A0A140VK56	TALDO1	--	--	--	--	--	--	0.72	0.67	**0.70**	--	--	--
AP-3 complex subunit mu-1	B4DRN6	AP3M1	--	--	--	--	--	--	0.66	0.73	**0.70**	--	--	--
Eukaryotic translation initiation factor 3 subunit F	B4DMT5	EIF3F	--	--	--	--	--	--	0.63	0.76	**0.70**	--	--	--
Ribonuclease UK114	A0A024R9H2	HRSP12	--	--	--	--	--	--	0.78	0.59	**0.69**	--	--	--
Twinfilin-1	Q12792	TWF1	--	--	--	--	--	--	0.75	0.62	**0.68**	--	--	--
SEC23-interacting protein	B4DWG1	SEC23IP	--	--	--	--	--	--	0.66	0.71	**0.68**	--	--	--
Ubiquitin fusion degradation protein 1 homolog	Q541A5	ufd1	--	--	--	--	--	--	0.67	0.69	**0.68**	--	--	--
NudC domain-containing protein 1	Q96RS6	NUDCD1	--	--	--	--	--	--	0.76	0.60	**0.68**	--	--	--
Hepatocyte growth factor-regulated tyrosine kinase substrate	B4E1E2	HGS	--	--	--	--	--	--	0.75	0.60	**0.67**	--	--	--
Transketolase	V9HWD9	TKT	--	--	--	--	--	--	0.74	0.61	**0.67**	--	--	--
Parathymosin	A0A158RFU3	PTMS	--	--	--	--	--	--	0.69	0.63	**0.66**	--	--	--
Methionine adenosyltransferase 2 subunit beta	A0A140VJP2	MAT2B	--	--	--	--	--	--	0.62	0.69	**0.65**	--	--	--
60 kDa SS-A/Ro ribonucleoprotein	A0A024R983	TROVE2	--	--	--	--	--	--	0.66	0.64	**0.65**	--	--	--
Nucleolar protein 11	Q9H8H0	NOL11	--	--	--	--	--	--	0.70	0.59	**0.64**	--	--	--
YTH domain-containing protein 1	J3QR07	YTHDC1	--	--	--	--	--	--	0.63	0.66	**0.64**	--	--	--
Ubiquitin carboxyl-terminal hydrolase 10	Q14694	USP10	--	--	--	--	--	--	0.60	0.67	**0.63**	--	--	--
PCI domain-containing protein 2	A0A024RDZ9	PCID2	--	--	--	--	--	--	0.60	0.64	**0.62**	--	--	--
Peroxiredoxin-5, mitochondrial	V9HW35	PRDX5	--	--	--	--	--	--	0.61	0.59	**0.60**	--	--	--
Syntaxin-binding protein 3	O00186	STXBP3	--	--	--	--	--	--	-0.74	-0.63	**-0.68**	--	--	--
Ras-related protein Rap-1A	A8KAH9	RAP1A	--	--	--	--	--	--	-0.64	-0.74	**-0.69**	--	--	--
Tripartite motif-containing protein 3	B7Z5Y8	TRIM3	--	--	--	--	--	--	-0.74	-0.69	**-0.72**	--	--	--
EGF-containing fibulin-like extracellular matrix protein 2	E9PRU1	EFEMP2	--	--	--	--	--	--	-0.60	-0.89	**-0.75**	--	--	--
Selenoprotein N	H9KV50	SEPN1	--	--	--	--	--	--	-0.74	-0.76	**-0.75**	--	--	--
Integrin beta	L7UUZ7	ITGB3	--	--	--	--	--	--	-0.65	-0.91	**-0.78**	--	--	--
PTB domain-containing engulfment adapter protein 1	B3KN52	GULP1	--	--	--	--	--	--	-0.80	-0.76	**-0.78**	--	--	--
Kin of IRRE-like protein 1	Q96J84	KIRREL	--	--	--	--	--	--	-0.95	-0.62	**-0.78**	--	--	--
Protein-tyrosine-phosphatase	Q86WJ2	PTPRK	--	--	--	--	--	--	-1.06	-0.59	**-0.83**	--	--	--
Leucyl-cystinyl aminopeptidase	B2RAK1	LNPEP	--	--	--	--	--	--	-1.07	-0.59	**-0.83**	--	--	--
Phospholipid scramblase 3	D3DTP7	PLSCR3	--	--	--	--	--	--	-0.94	-0.87	**-0.90**	--	--	--
Glycosyltransferase 8 domain-containing protein 1	A0A024R313	GLT8D1	--	--	--	--	--	--	-1.07	-0.87	**-0.97**	--	--	--
Desmoplakin	P15924	DSP	--	--	--	--	--	--	-0.62	-1.32	**-0.97**	--	--	--
Tetraspanin	B4DPP0	CD9	--	--	--	--	--	--	-1.16	-0.79	**-0.98**	--	--	--
	K7ENT6	TPM4	--	--	--	--	--	--	-1.29	-0.74	**-1.02**	--	--	--
Interleukin-6 receptor subunit beta	A0A0A0N0L5	IL6ST	--	--	--	--	--	--	-1.74	-0.71	**-1.22**	--	--	--
Thyrotropin-releasing hormone-degrading ectoenzyme	Q9UKU6	TRHDE	--	--	--	--	--	--	-1.62	-1.17	**-1.40**	--	--	--
Growth arrest-specific protein 6	B4DZY7	GAS6	--	--	--	--	--	--	-1.52	-1.41	**-1.47**	--	--	--
Antigen peptide transporter 2	Q5JNW1	TAP2	--	--	--	--	--	--	-1.58	-1.71	**-1.64**	--	--	--
Histone H1.2	P16403	HIST1H1C	--	--	--	--	--	--	-1.46	-1.89	**-1.67**	--	--	--
Bifunctional UDP-N-acetylglucosamine 2-epimerase/N-	Q6QNY7	GNE	--	--	--	--	--	--	--	--	--	1.94	2.74	**2.34**
Protein SON	P18583	SON	--	--	--	--	--	--	--	--	--	2.17	2.12	**2.14**
Angio-associated migratory cell protein	C9JG97	AAMP	--	--	--	--	--	--	--	--	--	1.74	1.93	**1.83**
Sjoegren syndrome/scleroderma autoantigen 1	H0YEB6	SSSCA1	--	--	--	--	--	--	--	--	--	1.89	1.40	**1.65**
Ubiquitin-conjugating enzyme E2 variant 2	A0M8W4	UBE2V2	--	--	--	--	--	--	--	--	--	2.04	1.22	**1.63**

***S3D Table**. Cont.*

Protein name	Uniprot	Symbol	6 hpi			12 hpi			24 hpi			36 hpi		
			Ad2(L)/ Mock(H)	Mock(L)/ Ad2(H)	Average	Ad2(L)/ Mock(H)	Mock(L)/ Ad2(H)	Average	Ad2(L)/ Mock(H)	Mock(L)/ Ad2(H)	Average	Ad2(L)/ Mock(H)	Mock(L)/ Ad2(H)	Average
Eukaryotic translation initiation factor 4 gamma 3	A0A0U1RQK7	EIF4G3	--	--	--	--	--	--	--	--	--	2.11	0.91	**1.51**
PITH domain-containing protein 1	Q9GZP4	PITHD1	--	--	--	--	--	--	--	--	--	2.28	0.70	**1.49**
SWI/SNF complex subunit SMARCC1	Q58EY4	SMARCC1	--	--	--	--	--	--	--	--	--	1.38	1.52	**1.45**
Probable 28S rRNA (cytosine(4447)-C(5))-methyltransferase	P46087	NOP2	--	--	--	--	--	--	--	--	--	1.66	1.06	**1.36**
Non-specific protein-tyrosine kinase	Q53EL3	CSK	--	--	--	--	--	--	--	--	--	1.68	0.98	**1.33**
E3 ubiquitin-protein ligase HECTD1	A0A087X2H1	HECTD1	--	--	--	--	--	--	--	--	--	1.79	0.81	**1.30**
Deoxycytidine kinase	F5CTF3	DCK	--	--	--	--	--	--	--	--	--	1.89	0.61	**1.25**
Enhancer of mRNA-decapping protein 4	Q6P2E9	EDC4	--	--	--	--	--	--	--	--	--	1.70	0.77	**1.24**
Bleomycin hydrolase	Q13867	BLMH	--	--	--	--	--	--	--	--	--	1.84	0.59	**1.21**
Replication factor C subunit 5	Q6LES9	RFC5	--	--	--	--	--	--	--	--	--	1.18	1.24	**1.21**
Deoxyuridine 5-triphosphate nucleotidohydrolase, mitochondrial	P33316	DUT	--	--	--	--	--	--	--	--	--	1.44	0.98	**1.21**
Eukaryotic translation initiation factor 4 gamma 1	E7EX73	EIF4G1	--	--	--	--	--	--	--	--	--	1.47	0.92	**1.19**
Phosphoglucomutase-2	Q96G03	PGM2	--	--	--	--	--	--	--	--	--	1.38	0.95	**1.16**
DNA mismatch repair protein Msh6	P52701	MSH6	--	--	--	--	--	--	--	--	--	1.06	1.17	**1.11**
40S ribosomal protein S30	E9PR30	FAU	--	--	--	--	--	--	--	--	--	1.04	1.09	**1.07**
	B4E106		--	--	--	--	--	--	--	--	--	1.22	0.83	**1.02**
Myosin-11	A0A024QZJ6	MYH11	--	--	--	--	--	--	--	--	--	1.03	1.01	**1.02**
MARCKS-related protein	P49006	MARCKSL1	--	--	--	--	--	--	--	--	--	1.02	0.98	**1.00**
ATP-dependent RNA helicase DDX39A	O00148	DDX39A	--	--	--	--	--	--	--	--	--	0.71	1.24	**0.97**
Striatin-3	A0AV58	STRN3	--	--	--	--	--	--	--	--	--	0.69	1.24	**0.96**
Profilin-2	C9J712	PFN2	--	--	--	--	--	--	--	--	--	0.90	1.01	**0.96**
40S ribosomal protein S11	P62280	RPS11	--	--	--	--	--	--	--	--	--	0.86	1.05	**0.95**
Pseudouridylate synthase 7 homolog	B3KY42	PUS7	--	--	--	--	--	--	--	--	--	1.15	0.71	**0.93**
Replication factor C subunit 3	B4DKE6	RFC3	--	--	--	--	--	--	--	--	--	0.92	0.94	**0.93**
Cell growth-regulating nucleolar protein	Q9NX58	LYAR	--	--	--	--	--	--	--	--	--	0.73	1.13	**0.93**
Proteasome-associated protein ECM29 homolog	Q5VYK3	ECM29	--	--	--	--	--	--	--	--	--	1.10	0.75	**0.92**
Tight junction protein ZO-2	A0A024R233	TJP2	--	--	--	--	--	--	--	--	--	1.05	0.76	**0.90**
60S ribosomal protein L27a	Q6NZ52	RPL27A	--	--	--	--	--	--	--	--	--	0.75	1.02	**0.88**
Scaffold attachment factor B1	B7Z2Z1	SAFB	--	--	--	--	--	--	--	--	--	1.05	0.70	**0.88**
Serine/arginine-rich splicing factor 6	Q59GY3	SRSF6	--	--	--	--	--	--	--	--	--	0.75	1.00	**0.87**
RNA-binding protein 28	A0A024R753	RBM28	--	--	--	--	--	--	--	--	--	1.08	0.65	**0.87**
Pre-mRNA-splicing factor ATP-dependent RNA helicase DHX15	O43143	DHX15	--	--	--	--	--	--	--	--	--	1.12	0.60	**0.86**
Charged multivesicular body protein 1a	B4DXN3	CHMP1A	--	--	--	--	--	--	--	--	--	0.81	0.91	**0.86**
CCAAT/enhancer-binding protein zeta	Q03701	CEBPZ	--	--	--	--	--	--	--	--	--	0.64	1.02	**0.83**
40S ribosomal protein S10	P46783	RPS10	--	--	--	--	--	--	--	--	--	0.82	0.79	**0.80**
Cellular nucleic acid-binding protein	A0A0S2Z4Q3	CNBP	--	--	--	--	--	--	--	--	--	0.71	0.89	**0.80**
Serine/arginine-rich splicing factor 10	Q5JRI1	SRSF10	--	--	--	--	--	--	--	--	--	0.88	0.72	**0.80**
Myb-binding protein 1A	Q9BQG0	MYBBP1A	--	--	--	--	--	--	--	--	--	0.64	0.94	**0.79**
DnaJ homolog subfamily A member 2	A0A024R6S1	DNAJA2	--	--	--	--	--	--	--	--	--	0.93	0.64	**0.78**
40S ribosomal protein S16	P62249	RPS16	--	--	--	--	--	--	--	--	--	0.92	0.64	**0.78**
Elongation factor 1-gamma	Q53YD7	EEF1G	--	--	--	--	--	--	--	--	--	0.78	0.77	**0.78**
MKI67 FHA domain-interacting nucleolar phosphoprotein	Q9BYG3	NIFK	--	--	--	--	--	--	--	--	--	0.82	0.73	**0.77**
40S ribosomal protein S20	P60866	RPS20	--	--	--	--	--	--	--	--	--	0.94	0.61	**0.77**
Protein RRP5 homolog	Q14690	PDCD11	--	--	--	--	--	--	--	--	--	0.66	0.88	**0.77**
Pre-mRNA-splicing regulator WTAP	Q15007	WTAP	--	--	--	--	--	--	--	--	--	0.80	0.74	**0.77**
60S ribosomal protein L22	Q7Z4W8	RPL22	--	--	--	--	--	--	--	--	--	0.92	0.60	**0.76**
T-complex protein 1 subunit beta	V9HW96	CCT2	--	--	--	--	--	--	--	--	--	0.73	0.78	**0.76**
Nucleoporin NUP188 homolog	Q5SRE5	NUP188	--	--	--	--	--	--	--	--	--	0.81	0.70	**0.76**

S3D Table. *Cont.*

Protein name	Uniprot	Symbol	6 hpi			12 hpi			24 hpi			36 hpi		
			Ad2(L)/ Mock(H)	Mock(L)/ Ad2(H)	Average	Ad2(L)/ Mock(H)	Mock(L)/ Ad2(H)	Average	Ad2(L)/ Mock(H)	Mock(L)/ Ad2(H)	Average	Ad2(L)/ Mock(H)	Mock(L)/ Ad2(H)	Average
Transcription factor A, mitochondrial	Q6LES8	TFAM	--	--	--	--	--	--	--	--	--	0.69	0.82	**0.76**
Calcium homeostasis endoplasmic reticulum protein	J3QK89	CHERP	--	--	--	--	--	--	--	--	--	0.74	0.76	**0.75**
Y-box-binding protein 3	A0A024RAQ1	CSDA	--	--	--	--	--	--	--	--	--	0.85	0.65	**0.75**
Uncharacterized protein C11orf98	A0A0B4J220	C11orf98	--	--	--	--	--	--	--	--	--	0.63	0.85	**0.74**
Splicing factor 3A subunit 2	Q05DF2	SF3A2	--	--	--	--	--	--	--	--	--	0.87	0.61	**0.74**
60S ribosome subunit biogenesis protein NIP7 homolog	Q9Y221	NIP7	--	--	--	--	--	--	--	--	--	0.85	0.61	**0.73**
Eukaryotic translation initiation factor 3 subunit M	Q7L2H7	EIF3M	--	--	--	--	--	--	--	--	--	0.59	0.86	**0.72**
40S ribosomal protein S12	P25398	RPS12	--	--	--	--	--	--	--	--	--	0.77	0.68	**0.72**
Thyroid hormone receptor-associated protein 3	Q9Y2W1	THRAP3	--	--	--	--	--	--	--	--	--	0.71	0.72	**0.72**
U2 snRNP-associated SURP motif-containing protein	O15042	U2SURP	--	--	--	--	--	--	--	--	--	0.80	0.63	**0.71**
WD40 repeat-containing protein SMU1	A0MNN4	SMU1	--	--	--	--	--	--	--	--	--	0.74	0.67	**0.71**
Cold shock domain-containing protein E1	A0A024R0E2	CSDE1	--	--	--	--	--	--	--	--	--	0.66	0.74	**0.70**
40S ribosomal protein S15a	B2R4W8	RPS15A	--	--	--	--	--	--	--	--	--	0.75	0.64	**0.70**
Protein mago nashi homolog	P61326	MAGOH	--	--	--	--	--	--	--	--	--	0.74	0.65	**0.70**
Elongation factor 1-beta	A0A024R3W7	EEF1B2	--	--	--	--	--	--	--	--	--	0.69	0.69	**0.69**
Transformer-2 protein homolog beta	Q8N1H4	TRA2B	--	--	--	--	--	--	--	--	--	0.64	0.74	**0.69**
Polyadenylate-binding protein	A0A024R9C1	PABPC1	--	--	--	--	--	--	--	--	--	0.61	0.76	**0.69**
Nucleolar complex protein 2 homolog	B3KNC3	NOC2L	--	--	--	--	--	--	--	--	--	0.73	0.63	**0.68**
40S ribosomal protein S29	P62273	RPS29	--	--	--	--	--	--	--	--	--	0.69	0.67	**0.68**
Far upstream element-binding protein 1	B4E0X8	FUBP1	--	--	--	--	--	--	--	--	--	0.65	0.70	**0.68**
T-complex protein 1 subunit delta	A8K3C3	CCT4	--	--	--	--	--	--	--	--	--	0.74	0.62	**0.68**
Eukaryotic translation initiation factor 5B	A0A087WUT6	EIF5B	--	--	--	--	--	--	--	--	--	0.69	0.66	**0.67**
Cytoskeleton-associated protein 5	Q14008	CKAP5	--	--	--	--	--	--	--	--	--	0.60	0.71	**0.66**
Poly(U)-binding-splicing factor PUF60	A0A0J9YYL3	PUF60	--	--	--	--	--	--	--	--	--	0.69	0.60	**0.65**
Nuclear pore complex protein Nup155	B4DLT2	NUP155	--	--	--	--	--	--	--	--	--	0.69	0.60	**0.64**
T-complex protein 1 subunit gamma	B3KX11	CCT3	--	--	--	--	--	--	--	--	--	0.68	0.59	**0.63**
Protein transport protein Sec24D	B3KM89	SEC24D	--	--	--	--	--	--	--	--	--	0.60	0.59	**0.60**
Lysosome membrane protein 2	A0A024RDG6	SCARB2	--	--	--	--	--	--	--	--	--	-0.62	-0.67	**-0.64**
Tropomyosin alpha-4 chain	V9HW56	TPM4	--	--	--	--	--	--	--	--	--	-0.64	-0.66	**-0.65**
Aspartyl/asparaginyl beta-hydroxylase	Q12797	ASPH	--	--	--	--	--	--	--	--	--	-0.59	-0.72	**-0.65**
CD44 antigen	B4DN59	CD44	--	--	--	--	--	--	--	--	--	-0.70	-0.62	**-0.66**
Axin interactor, dorsalization-associated protein	B7Z7D5	AIDA	--	--	--	--	--	--	--	--	--	-0.65	-0.69	**-0.67**
Spectrin beta chain, non-erythrocytic 1	B2ZZ89	SPTBN1	--	--	--	--	--	--	--	--	--	-0.73	-0.62	**-0.68**
Spectrin alpha chain, non-erythrocytic 1	A0A024R889	SPTAN1	--	--	--	--	--	--	--	--	--	-0.70	-0.66	**-0.68**
Dolichyl-diphosphooligosaccharide--protein glycosyltransferase subunit	A0A024R3J7	STT3A	--	--	--	--	--	--	--	--	--	-0.72	-0.65	**-0.68**
Laminin subunit alpha-5	O15230	LAMA5	--	--	--	--	--	--	--	--	--	-0.59	-0.78	**-0.68**
	B3KNG6	CALU	--	--	--	--	--	--	--	--	--	-0.70	-0.67	**-0.68**
Collagen alpha-1(XIV) chain	Q05707	COL14A1	--	--	--	--	--	--	--	--	--	-0.67	-0.74	**-0.70**
ER membrane protein complex subunit 3	Q9P0I2	EMC3	--	--	--	--	--	--	--	--	--	-0.79	-0.62	**-0.70**
Polypeptide N-acetylgalactosaminyltransferase	A0A024RC48	GALNT1	--	--	--	--	--	--	--	--	--	-0.72	-0.69	**-0.71**
Neutral cholesterol ester hydrolase 1	A0A0R4J2G3	NCEH1	--	--	--	--	--	--	--	--	--	-0.60	-0.81	**-0.71**
Leucine zipper protein 1	Q86V48	LUZP1	--	--	--	--	--	--	--	--	--	-0.62	-0.80	**-0.71**
Catenin alpha-1	P35221	CTNNA1	--	--	--	--	--	--	--	--	--	-0.62	-0.80	**-0.71**
Deoxyribonuclease-2-alpha	C0LQF2	DNASE2	--	--	--	--	--	--	--	--	--	-0.68	-0.75	**-0.72**
Histone H2A type 2-C	Q16777	HIST2H2A	--	--	--	--	--	--	--	--	--	-0.63	-0.82	**-0.72**
Beta-mannosidase	A8K6D3	MANBA	--	--	--	--	--	--	--	--	--	-0.66	-0.78	**-0.72**
Histone H4	B2R4R0	HIST1H4H	--	--	--	--	--	--	--	--	--	-0.62	-0.82	**-0.72**
Epididymal secretory protein E1	J3KMY5	NPC2	--	--	--	--	--	--	--	--	--	-0.82	-0.63	**-0.73**

***S3D Table**. Cont.*

Protein name	Uniprot	Symbol	6 hpi			12 hpi			24 hpi			36 hpi		
			Ad2(L)/ Mock(H)	Mock(L)/ Ad2(H)	Average	Ad2(L)/ Mock(H)	Mock(L)/ Ad2(H)	Average	Ad2(L)/ Mock(H)	Mock(L)/ Ad2(H)	Average	Ad2(L)/ Mock(H)	Mock(L)/ Ad2(H)	Average
Polypeptide N-acetylgalactosaminyltransferase 5	Q7Z7M9	GALNT5	--	--	--	--	--	--	--	--	--	-0.67	-0.79	**-0.73**
Histone H2B type 2-E	Q16778	HIST2H2BE	--	--	--	--	--	--	--	--	--	-0.71	-0.76	**-0.74**
Synaptosomal-associated protein	A8K287	SNAP23	--	--	--	--	--	--	--	--	--	-0.68	-0.80	**-0.74**
Solute carrier family 35 member F6	Q8N357	SLC35F6	--	--	--	--	--	--	--	--	--	-0.76	-0.73	**-0.74**
Methionine-R-sulfoxide reductase B3	F5H7C4	MSRB3	--	--	--	--	--	--	--	--	--	-0.92	-0.60	**-0.76**
Vitamin K-dependent gamma-carboxylase	A7YA96	GGCX	--	--	--	--	--	--	--	--	--	-0.79	-0.74	**-0.77**
Golgin subfamily A member 2	A0A0C4DGS5	GOLGA2	--	--	--	--	--	--	--	--	--	-0.81	-0.73	**-0.77**
Voltage-dependent calcium channel subunit alpha-2/delta-1	P54289	CACNA2D1	--	--	--	--	--	--	--	--	--	-0.71	-0.84	**-0.78**
Protein tyrosine phosphatase type IVA 2	Q12974	PTP4A2	--	--	--	--	--	--	--	--	--	-0.70	-0.86	**-0.78**
	B3KM58	DKFZp686	--	--	--	--	--	--	--	--	--	-0.81	-0.76	**-0.79**
Guanine nucleotide-binding protein G(q) subunit alpha	A0A024R240	GNAQ	--	--	--	--	--	--	--	--	--	-0.59	-0.98	**-0.79**
Adenosine 3-phospho 5-phosphosulfate transporter 1	Q8TB61	SLC35B2	--	--	--	--	--	--	--	--	--	-0.86	-0.73	**-0.80**
Ras-related protein Rap-2b	Q5JQ44	RAP2B	--	--	--	--	--	--	--	--	--	-0.65	-0.95	**-0.80**
Basement membrane-specific heparan sulfate proteoglycan core protein	A0A024RAB6	HSPG2	--	--	--	--	--	--	--	--	--	-0.96	-0.65	**-0.81**
Ras-related protein Ral-A	P11233	RALA	--	--	--	--	--	--	--	--	--	-0.73	-0.89	**-0.81**
EMILIN-1	A0A0C4DFX3	EMILIN1	--	--	--	--	--	--	--	--	--	-0.86	-0.77	**-0.82**
	Q6LAF9		--	--	--	--	--	--	--	--	--	-0.80	-0.84	**-0.82**
Mitofusin-1	Q4AEJ3	MFN1	--	--	--	--	--	--	--	--	--	-1.02	-0.64	**-0.83**
Lipase maturation factor 2	Q9BU23	LMF2	--	--	--	--	--	--	--	--	--	-0.91	-0.78	**-0.84**
Ribosome-binding protein 1	A7BI36	RRBP1	--	--	--	--	--	--	--	--	--	-0.83	-0.86	**-0.85**
Extracellular matrix protein 1	A0A140VJI7	ECM1	--	--	--	--	--	--	--	--	--	-1.04	-0.66	**-0.85**
CKLF-like MARVEL transmembrane domain-containing protein 6	Q9NX76	CMTM6	--	--	--	--	--	--	--	--	--	-0.96	-0.74	**-0.85**
Mesoderm-specific transcript homolog protein	C9JWU9	MEST	--	--	--	--	--	--	--	--	--	-0.86	-0.85	**-0.85**
NFU1 iron-sulfur cluster scaffold homolog, mitochondrial	Q9UMS0	NFU1	--	--	--	--	--	--	--	--	--	-0.63	-1.08	**-0.86**
GTPase KRas	A0A024RAV5	KRAS	--	--	--	--	--	--	--	--	--	-0.83	-0.88	**-0.86**
Syntaxin-12	Q6LEU0	STX12	--	--	--	--	--	--	--	--	--	-0.83	-0.92	**-0.88**
Syntaxin-4	Q12846	STX4	--	--	--	--	--	--	--	--	--	-0.68	-1.08	**-0.88**
Syntaxin-7	O15400	STX7	--	--	--	--	--	--	--	--	--	-0.85	-0.91	**-0.88**
Probable glutathione peroxidase 8	Q8TED1	GPX8	--	--	--	--	--	--	--	--	--	-0.84	-0.93	**-0.88**
Lysyl oxidase homolog 2	Q9Y4K0	LOXL2	--	--	--	--	--	--	--	--	--	-1.16	-0.64	**-0.90**
Phospholipase D3	A0A024R0Q4	PLD3	--	--	--	--	--	--	--	--	--	-0.92	-0.93	**-0.93**
Myeloid-associated differentiation marker	B4DG42	MYADM	--	--	--	--	--	--	--	--	--	-0.87	-0.99	**-0.93**
Protein phosphatase 1 regulatory subunit	B2RAH5	PPP1R12A	--	--	--	--	--	--	--	--	--	-0.60	-1.28	**-0.94**
Probable global transcription activator SNF2L1	Q86UA8	SMARCA1	--	--	--	--	--	--	--	--	--	-0.68	-1.21	**-0.94**
Prolyl endopeptidase FAP	A0A172Q3A0	FAP	--	--	--	--	--	--	--	--	--	-1.21	-0.69	**-0.95**
45 kDa calcium-binding protein	A0A024R084	SDF4	--	--	--	--	--	--	--	--	--	-0.95	-0.96	**-0.95**
Disintegrin and metalloproteinase domain-containing protein 9	Q13443	ADAM9	--	--	--	--	--	--	--	--	--	-0.88	-1.03	**-0.95**
	Q96CZ9	CDH11	--	--	--	--	--	--	--	--	--	-1.21	-0.70	**-0.95**
Glutaminase kidney isoform, mitochondrial	A8K132	GLS	--	--	--	--	--	--	--	--	--	-1.17	-0.76	**-0.96**
	H3BN98		--	--	--	--	--	--	--	--	--	-0.86	-1.07	**-0.97**
Sterol O-acyltransferase 1	P35610	SOAT1	--	--	--	--	--	--	--	--	--	-0.65	-1.29	**-0.97**
Prolow-density lipoprotein receptor-related protein 1	Q07954	LRP1	--	--	--	--	--	--	--	--	--	-1.17	-0.80	**-0.98**
Golgi apparatus protein 1	Q92896	GLG1	--	--	--	--	--	--	--	--	--	-1.06	-0.91	**-0.99**
Trans-Golgi network integral membrane protein 2	J3KQ45	TGOLN2	--	--	--	--	--	--	--	--	--	-0.98	-1.03	**-1.00**
Tetraspanin	E9PJK1	CD81	--	--	--	--	--	--	--	--	--	-1.10	-0.90	**-1.00**
Alpha-1,6-mannosylglycoprotein 6-beta-N-acetylglucosaminyltransferase	Q09328	MGAT5	--	--	--	--	--	--	--	--	--	-1.40	-0.63	**-1.01**
Latent-transforming growth factor beta-binding protein 1	Q14766	LTBP1	--	--	--	--	--	--	--	--	--	-1.11	-0.93	**-1.02**
Caveolin	Q2TNI1	CAV1	--	--	--	--	--	--	--	--	--	-1.07	-1.02	**-1.05**

***S3D Table**. Cont.*

Protein name	Uniprot	Symbol	6 hpi			12 hpi			24 hpi			36 hpi		
			Ad2(L)/ Mock(H)	Mock(L)/ Ad2(H)	Average	Ad2(L)/ Mock(H)	Mock(L)/ Ad2(H)	Average	Ad2(L)/ Mock(H)	Mock(L)/ Ad2(H)	Average	Ad2(L)/ Mock(H)	Mock(L)/ Ad2(H)	Average
Endoplasmic reticulum mannosyl-oligosaccharide 1,2-alpha-mannosidase	Q9UKM7	MAN1B1	--	--	--	--	--	--	--	--	--	-1.18	-0.92	**-1.05**
Selenoprotein S	Q6GYA4	SELS	--	--	--	--	--	--	--	--	--	-1.16	-0.95	**-1.05**
Rab11 family-interacting protein 5	Q2Z1P3	GAF1	--	--	--	--	--	--	--	--	--	-1.16	-0.95	**-1.06**
Peroxidasin homolog	Q92626	PXDN	--	--	--	--	--	--	--	--	--	-1.17	-0.97	**-1.07**
Protein kinase C delta-binding protein	Q969G5	PRKCDBP	--	--	--	--	--	--	--	--	--	-1.52	-0.63	**-1.08**
Syndecan	E7ESK6	SDC2	--	--	--	--	--	--	--	--	--	-0.98	-1.18	**-1.08**
	A1A5C4	RRBP1	--	--	--	--	--	--	--	--	--	-1.39	-0.77	**-1.08**
5-nucleotidase	Q6NZX3	NT5E	--	--	--	--	--	--	--	--	--	-1.14	-1.02	**-1.08**
Sphingomyelin phosphodiesterase	E9LUE6	SMPD1	--	--	--	--	--	--	--	--	--	-1.45	-0.77	**-1.11**
Histone H1.5	P16401	HIST1H1B	--	--	--	--	--	--	--	--	--	-1.53	-0.70	**-1.12**
Nucleobindin-1	Q53GX6	NUCB1	--	--	--	--	--	--	--	--	--	-1.25	-1.04	**-1.14**
	Q5T6U8	HMGA1	--	--	--	--	--	--	--	--	--	-0.86	-1.43	**-1.15**
Follistatin-related protein 1	Q12841	FSTL1	--	--	--	--	--	--	--	--	--	-1.15	-1.18	**-1.17**
Nicastrin	Q92542	NCSTN	--	--	--	--	--	--	--	--	--	-1.31	-1.03	**-1.17**
Tetraspanin	F8VNT9	CD63	--	--	--	--	--	--	--	--	--	-1.54	-0.80	**-1.17**
Nidogen-2	Q14112	NID2	--	--	--	--	--	--	--	--	--	-1.37	-1.00	**-1.19**
Tumor protein p53-inducible protein 11	E9PN66	TP53I11	--	--	--	--	--	--	--	--	--	-1.77	-0.65	**-1.21**
Endothelin-converting enzyme 1	B4DKB2	ECE1	--	--	--	--	--	--	--	--	--	-1.48	-0.99	**-1.24**
SUN domain-containing protein 2	A0A024R1P7	UNC84B	--	--	--	--	--	--	--	--	--	-1.16	-1.32	**-1.24**
Coiled-coil domain-containing protein 50	Q8IVM0	CCDC50	--	--	--	--	--	--	--	--	--	-1.19	-1.30	**-1.24**
DNA repair protein RAD50	Q92878	RAD50	--	--	--	--	--	--	--	--	--	-1.25	-1.34	**-1.30**
Lactadherin	B3KTQ2	MFGE8	--	--	--	--	--	--	--	--	--	-1.90	-0.71	**-1.31**
TATA-binding protein-associated factor 2N	Q92804	TAF15	--	--	--	--	--	--	--	--	--	-1.32	-1.30	**-1.31**
Syntenin-1	A0A024R7Z5	SDCBP	--	--	--	--	--	--	--	--	--	-1.79	-0.89	**-1.34**
Laminin subunit alpha-4	Q5D044	LAMA4	--	--	--	--	--	--	--	--	--	-0.85	-1.93	**-1.39**
Metalloproteinase inhibitor 1	Q5H9A7	TIMP1	--	--	--	--	--	--	--	--	--	-1.59	-1.34	**-1.47**
A disintegrin and metalloproteinase with thrombospondin motifs 1	B2RB33	ADAMTS1	--	--	--	--	--	--	--	--	--	-1.95	-1.26	**-1.61**
Disintegrin and metalloproteinase domain-containing protein 10	A0A024R5U5	ADAM10	--	--	--	--	--	--	--	--	--	-1.52	-1.70	**-1.61**
Methyl-CpG-binding protein 2	A0A140VKC4	MECP2	--	--	--	--	--	--	--	--	--	-1.67	-1.57	**-1.62**
Insulin-like growth factor-binding protein 7	Q16270	IGFBP7	--	--	--	--	--	--	--	--	--	-2.12	-1.56	**-1.84**
	D3DUZ3	IFI16	--	--	--	--	--	--	--	--	--	-2.61	-1.40	**-2.01**
SPARC	D3DQH8	SPARC	--	--	--	--	--	--	--	--	--	-2.37	-1.79	**-2.08**
Glypican-1	P35052	GPC1	--	--	--	--	--	--	--	--	--	-1.93	-2.55	**-2.24**
Pentraxin-related protein PTX3	P26022	PTX3	--	--	--	--	--	--	--	--	--	-2.63	-2.60	**-2.62**
Transcriptional enhancer factor TEF-1	H0YE88	TEAD1	--	--	--	--	--	--	--	--	--	-3.32	-2.07	**-2.70**
Tyrosine-protein kinase receptor	M1VE83	R34	--	--	--	--	--	--	--	--	--	-2.95	-2.90	**-2.93**
Mth938 domain-containing protein	E9PNP3	AAMDC	1.08	2.05	1.56	2.07	2.12	**2.10**	--	--	--	--	--	--
	B2R6L0		1.12	1.38	1.25	1.56	1.49	**1.53**	--	--	--	--	--	--
Protein-glutamate O-methyltransferase	Q9H993	ARMT1	1.26	1.24	1.25	1.59	0.99	**1.29**	--	--	--	--	--	--
Haloacid dehalogenase-like hydrolase domain-containing protein 2	K7ER15	HDHD2	1.03	1.35	1.19	--	--	--	--	--	--	1.46	0.70	**1.08**
NADP-dependent malic enzyme	P48163	ME1	1.21	1.15	1.18	1.37	1.09	**1.23**	--	--	--	--	--	--
Flavin reductase (NADPH)	V9HWI1	BLVRB	0.96	1.39	1.18	--	--	--	--	--	--	1.02	0.82	**0.92**
Alcohol dehydrogenase 1B	F5HB16	ADH1B	1.12	1.21	1.16	1.91	0.89	**1.40**	--	--	--	--	--	--
Protein phosphatase 1 regulatory subunit 14B	Q96C90	PPP1R14B	1.30	0.99	1.14	1.35	1.19	**1.27**	--	--	--	--	--	--
Tissue factor pathway inhibitor 2	Q8NE89	TFPI2	1.34	0.88	1.11	2.20	1.51	**1.85**	--	--	--	--	--	--
Glutamate--cysteine ligase regulatory subunit	P48507	GCLM	0.97	1.20	1.08	1.39	0.92	**1.16**	--	--	--	--	--	--
cGMP-dependent protein kinase 1	Q13976	PRKG1	1.21	0.95	1.08	1.20	0.84	**1.02**	--	--	--	--	--	--
Serine/threonine-protein kinase PAK 2	Q13177	PAK2	0.94	1.21	1.08	1.11	0.93	**1.02**	--	--	--	--	--	--

S3D Table. *Cont.*

Protein name	Uniprot	Symbol	6 hpi			12 hpi			24 hpi			36 hpi		
			Ad2(L)/ Mock(H)	Mock(L)/ Ad2(H)	Average	Ad2(L)/ Mock(H)	Mock(L)/ Ad2(H)	Average	Ad2(L)/ Mock(H)	Mock(L)/ Ad2(H)	Average	Ad2(L)/ Mock(H)	Mock(L)/ Ad2(H)	Average
Transgelin	Q5U0D2	TAGLN	0.73	1.40	**1.06**	0.64	1.25	**0.95**	--	--	--	--	--	--
Inosine triphosphate pyrophosphatase	A0A0S2Z3W7	ITPA	0.86	1.26	**1.06**	--	--	--	1.05	1.29	**1.17**	--	--	--
Synaptic vesicle membrane protein VAT-1 homolog-like	A8K288	VAT1L	0.90	1.21	**1.06**	0.99	1.14	**1.07**	--	--	--	--	--	--
Neurochondrin	Q9UBB6	NCDN	0.86	1.25	**1.06**	--	--	--	1.41	1.42	**1.41**	--	--	--
Serine/threonine-protein kinase Nek9	A0A024R6D1	NEK9	1.21	0.82	**1.02**	1.08	0.61	**0.85**	--	--	--	--	--	--
Trans-3-hydroxy-L-proline dehydratase	Q96EM0	L3HYPDH	1.18	0.84	**1.01**	1.21	1.41	**1.31**	--	--	--	--	--	--
ADP-ribosylation factor GTPase-activating protein 3	A0A024R4U0	ARFGAP3	1.16	0.86	**1.01**	1.40	0.86	**1.13**	--	--	--	--	--	--
C-Jun-amino-terminal kinase-interacting protein 4	A0A087X2D8	SPAG9	1.17	0.83	**1.00**	--	--	--	1.09	0.94	**1.02**	--	--	--
Chloride intracellular channel protein	Q6FIC5	CLIC4	0.82	1.13	**0.98**	1.01	0.89	**0.95**	--	--	--	--	--	--
UTP--glucose-1-phosphate uridylyltransferase	Q16851	UGP2	0.77	1.17	**0.97**	1.02	1.00	**1.01**	--	--	--	--	--	--
N-terminal Xaa-Pro-Lys N-methyltransferase 1	A0A024R8E4	NTMT1	0.61	1.32	**0.97**	0.87	1.02	**0.94**	--	--	--	--	--	--
Migration and invasion enhancer 1	Q9BRT3	MIEN1	0.83	1.09	**0.96**	1.20	1.10	**1.15**	--	--	--	--	--	--
von Willebrand factor A domain-containing protein 5A	A8K6N3	VWA5A	1.18	0.74	**0.96**	--	--	--	2.39	1.19	**1.79**	--	--	--
WD repeat-containing protein 1	Q53GN4	WDR1	0.83	1.07	**0.95**	0.86	0.87	**0.87**	--	--	--	--	--	--
Adenylyl cyclase-associated protein	D3DPU2	CAP1	0.71	1.13	**0.92**	0.77	0.90	**0.83**	--	--	--	--	--	--
Uridine 5-monophosphate synthase	B5LY71	UMPS	0.86	0.96	**0.91**	--	--	--	1.68	1.73	**1.71**	--	--	--
AMP deaminase 2	H0Y360	AMPD2	0.76	1.05	**0.91**	1.16	0.82	**0.99**	--	--	--	--	--	--
Heat shock protein beta-1	V9HW43	HSPB1	0.79	1.02	**0.90**	0.72	0.71	**0.71**	--	--	--	--	--	--
Methylthioribose-1-phosphate isomerase	Q9BV20	MRI1	0.70	1.10	**0.90**	1.17	0.61	**0.89**	--	--	--	--	--	--
Adenylyl cyclase-associated protein 2	P40123	CAP2	0.73	1.07	**0.90**	0.64	0.82	**0.73**	--	--	--	--	--	--
Perilipin-3	O60664	PLIN3	0.67	1.09	**0.88**	0.93	0.88	**0.91**	--	--	--	--	--	--
Proline synthase co-transcribed bacterial homolog protein	D3DSW3	PROSC	0.74	1.00	**0.87**	0.96	1.20	**1.08**	--	--	--	--	--	--
Echinoderm microtubule-associated protein-like 1	F8W717	EML1	0.79	0.90	**0.85**	0.83	0.91	**0.87**	--	--	--	--	--	--
Dipeptidyl peptidase 9	Q86TI2	DPP9	0.67	0.98	**0.83**	0.93	0.68	**0.80**	--	--	--	--	--	--
N(G),N(G)-dimethylarginine dimethylaminohydrolase 2	V9HW53	DDAH2	0.77	0.84	**0.81**	1.12	0.68	**0.90**	--	--	--	--	--	--
Protein PBDC1	Q9BVG4	PBDC1	0.72	0.88	**0.80**	1.15	0.87	**1.01**	--	--	--	--	--	--
Proteasome assembly chaperone 3	A0A024R806	PSMG3	0.91	0.69	**0.80**	1.07	0.83	**0.95**	--	--	--	--	--	--
Niban-like protein 1	A0A024R872	FAM129B	0.63	0.97	**0.80**	0.65	0.74	**0.70**	--	--	--	--	--	--
Kinesin light chain 1	Q7RTQ8	KNS2	0.66	0.93	**0.79**	0.68	0.83	**0.76**	--	--	--	--	--	--
Actin-related protein 3	A0A024RAI1	ACTR3	0.68	0.90	**0.79**	0.84	0.77	**0.80**	--	--	--	--	--	--
Actin-related protein 2/3 complex subunit 1B	A4D275	ARPC1B	0.66	0.91	**0.78**	0.85	0.69	**0.77**	--	--	--	--	--	--
Hsc70-interacting protein	B4E0U6	ST13	0.64	0.90	**0.77**	0.96	0.59	**0.78**	--	--	--	--	--	--
Translation initiation factor eIF-2B subunit alpha	Q14232	EIF2B1	0.74	0.81	**0.77**	0.63	0.67	**0.65**	--	--	--	--	--	--
Sorting nexin-6	A8K885	SNX6	0.70	0.84	**0.77**	0.92	0.66	**0.79**	--	--	--	--	--	--
Dynein light chain Tctex-type 3	A6NGJ0	DYNLT3	0.61	0.92	**0.76**	0.62	0.83	**0.72**	--	--	--	--	--	--
Inhibitor of nuclear factor kappa-B kinase subunit beta	B4DP95	IKBKB	0.77	0.75	**0.76**	0.81	0.68	**0.74**	--	--	--	--	--	--
Glycogen phosphorylase, liver form	P06737	PYGL	0.64	0.80	**0.72**	--	--	--	0.87	0.65	**0.76**	--	--	--
Glycogen synthase kinase-3 beta	Q6FI27	GSK3B	0.79	0.65	**0.72**	0.83	0.59	**0.71**	--	--	--	--	--	--
Moesin	V9HWC0	MSN	0.64	0.79	**0.71**	0.82	0.60	**0.71**	--	--	--	--	--	--
Cyclin-dependent-like kinase 5	A0A090N7W4	CDK5	0.80	0.59	**0.70**	--	--	--	0.83	0.97	**0.90**	--	--	--
Coatomer subunit epsilon	Q53HJ6	COPE	0.71	0.68	**0.69**	0.90	0.65	**0.77**	--	--	--	--	--	--
Translational activator GCN1	E1NZA1	GCN1L1	0.71	0.67	**0.69**	0.74	0.72	**0.73**	--	--	--	--	--	--
Translation initiation factor eIF-2B subunit epsilon	Q13144	EIF2B5	0.63	0.74	**0.68**	--	--	--	0.86	0.71	**0.78**	--	--	--
Microtubule-associated protein 1A	E9PGC8	MAP1A	0.75	0.60	**0.68**	0.62	0.70	**0.66**	--	--	--	--	--	--
SPATS2-like protein	A0A024R3V0	DNAPTP6	0.63	0.69	**0.66**	--	--	--	--	--	--	-1.94	-1.88	**-1.91**
Coatomer subunit beta	P53618	COPB1	0.67	0.62	**0.64**	0.83	0.67	**0.75**	--	--	--	--	--	--
UV excision repair protein RAD23 homolog B	B4DEA3	RAD23B	0.63	0.63	**0.63**	--	--	--	0.74	0.85	**0.79**	--	--	--
Collagen alpha-1(III) chain	P02461	COL3A1	-0.86	-1.72	**-1.29**	-0.71	-1.66	**-1.18**	--	--	--	--	--	--

***S3D Table*. Cont.**

Protein name	Uniprot	Symbol	6 hpi			12 hpi			24 hpi			36 hpi		
			Ad2(L)/ Mock(H)	Mock(L)/ Ad2(H)	Average	Ad2(L)/ Mock(H)	Mock(L)/ Ad2(H)	Average	Ad2(L)/ Mock(H)	Mock(L)/ Ad2(H)	Average	Ad2(L)/ Mock(H)	Mock(L)/ Ad2(H)	Average
Aldose 1-epimerase	Q96C23	GALM	--	--	--	1.33	1.74	**1.54**	1.49	0.81	**1.15**	--	--	--
Glucosamine 6-phosphate N-acetyltransferase	A0A024R649	GNPNAT1	--	--	--	1.60	1.11	**1.36**	1.37	1.10	**1.23**	--	--	--
	C7DJS2	GSTP1	--	--	--	1.31	1.27	**1.29**	0.74	1.18	**0.96**	--	--	--
Protein MEMO1	Q9Y316	MEMO1	--	--	--	1.47	1.10	**1.28**	--	--	--	0.98	0.72	**0.85**
Ubiquitin-like modifier-activating enzyme 5	Q9GZZ9	UBA5	--	--	--	1.42	1.05	**1.23**	1.36	1.24	**1.30**	--	--	--
Carbonyl reductase [NADPH] 3	V9HW40	CBR3	--	--	--	1.28	1.14	**1.21**	0.71	0.88	**0.79**	--	--	--
Zinc finger CCCH-type antiviral protein 1-like	Q96H79	ZC3HAV1L	--	--	--	1.08	1.31	**1.19**	0.88	0.62	**0.75**	--	--	--
Mitogen-activated protein kinase	A0A024RD15	MAPK14	--	--	--	1.38	0.92	**1.15**	1.15	1.25	**1.20**	--	--	--
Pyruvate kinase	A0A024R5Z9	PKM2	--	--	--	1.13	1.16	**1.14**	1.52	1.29	**1.40**	--	--	--
Ubiquitin-like-conjugating enzyme ATG3	Q9NT62	ATG3	--	--	--	1.10	1.17	**1.14**	--	--	--	2.01	1.13	**1.57**
Endothelial differentiation-related factor 1	O60869	EDF1	--	--	--	0.90	1.33	**1.11**	1.30	1.99	**1.65**	--	--	--
Deoxycytidylate deaminase	P32321	DCTD	--	--	--	0.99	1.23	**1.11**	1.13	1.03	**1.08**	--	--	--
ATP-binding cassette sub-family F member 3	B4DRU9	ABCF3	--	--	--	1.24	0.95	**1.10**	1.10	1.33	**1.21**	--	--	--
STE20-like serine/threonine-protein kinase	Q9H2G2	SLK	--	--	--	1.14	0.93	**1.03**	--	--	--	1.40	0.83	**1.12**
Glycogen synthase kinase-3 alpha	Q68D16	GSK3A	--	--	--	0.86	1.13	**0.99**	1.61	1.56	**1.59**	--	--	--
BRISC complex subunit Abro1	Q15018	FAM175B	--	--	--	1.01	0.97	**0.99**	1.53	0.62	**1.08**	--	--	--
Small ubiquitin-related modifier	A0A024R8S3	SUMO2	--	--	--	0.87	1.09	**0.98**	--	--	--	0.93	0.89	**0.91**
Eukaryotic translation initiation factor 1	Q6IAV3	SUI1	--	--	--	0.69	1.24	**0.97**	0.90	1.26	**1.08**	--	--	--
Tyrosine-protein phosphatase non-receptor type 12	Q05209	PTPN12	--	--	--	0.77	1.12	**0.95**	0.62	1.16	**0.89**	--	--	--
Protein transport protein Sec16A	F1T0I1	SEC16A	--	--	--	1.01	0.88	**0.95**	0.61	0.85	**0.73**	--	--	--
UPF0160 protein MYG1, mitochondrial	F8VR84	MST024	--	--	--	0.99	0.90	**0.94**	0.88	0.68	**0.78**	--	--	--
Nuclear factor NF-kappa-B p100 subunit	Q00653	NFKB2	--	--	--	0.97	0.89	**0.93**	1.09	0.98	**1.04**	--	--	--
Kinesin light chain 2	Q9H0B6	KLC2	--	--	--	1.01	0.80	**0.90**	--	--	--	0.85	0.95	**0.90**
Intraflagellar transport protein 27 homolog	F5GZ09	IFT27	--	--	--	0.85	0.93	**0.89**	1.42	1.45	**1.44**	--	--	--
26S proteasome non-ATPase regulatory subunit 5	A8K4T6	PSMD5	--	--	--	1.01	0.77	**0.89**	1.09	0.84	**0.97**	--	--	--
Signal transducer and activator of transcription	Q9BQD2	STAT6	--	--	--	0.91	0.87	**0.89**	0.65	0.93	**0.79**	--	--	--
Ubiquitin-conjugating enzyme E2 Z	B4DL66	UBE2Z	--	--	--	0.71	1.04	**0.88**	0.76	0.77	**0.76**	--	--	--
UDP-N-acetylhexosamine pyrophosphorylase	Q16222	UAP1	--	--	--	0.69	1.04	**0.87**	0.64	0.65	**0.64**	--	--	--
Nuclear receptor-binding protein	B4DW31	NRBP1	--	--	--	0.71	1.00	**0.85**	0.85	1.19	**1.02**	--	--	--
Serine/threonine-protein phosphatase	A0A024R861	PPP6C	--	--	--	0.98	0.71	**0.85**	0.98	1.15	**1.06**	--	--	--
Glutathione peroxidase	K7ERP4	GPX4	--	--	--	0.92	0.75	**0.83**	0.99	0.91	**0.95**	--	--	--
LIM and SH3 domain protein 1	A0A024R1S8	LASP1	--	--	--	0.84	0.81	**0.82**	0.94	0.86	**0.90**	--	--	--
CB1 cannabinoid receptor-interacting protein 1	B8ZZB8	CNRIP1	--	--	--	0.90	0.74	**0.82**	0.98	0.80	**0.89**	--	--	--
Translation initiation factor eIF-2B subunit gamma	Q9NR50	EIF2B3	--	--	--	0.75	0.89	**0.82**	0.63	0.60	**0.61**	--	--	--
Filamin-binding LIM protein 1	Q8WUP2	FBLIM1	--	--	--	0.63	0.98	**0.81**	0.64	0.68	**0.66**	--	--	--
Caspase-4	P49662	CASP4	--	--	--	0.61	0.97	**0.79**	1.00	0.77	**0.89**	--	--	--
Glyoxylate reductase/hydroxypyruvate reductase	Q9UBQ7	GRHPR	--	--	--	0.87	0.71	**0.79**	1.04	0.87	**0.95**	--	--	--
Large proline-rich protein BAG6	A0A024RCR6	BAT3	--	--	--	0.74	0.83	**0.78**	1.25	1.03	**1.14**	--	--	--
Charged multivesicular body protein 4b	Q9H444	CHMP4B	--	--	--	0.86	0.66	**0.76**	1.00	0.92	**0.96**	--	--	--
BAG family molecular chaperone regulator 3	Q53GY1	BAG3	--	--	--	0.82	0.68	**0.75**	1.65	1.60	**1.62**	--	--	--
RNA 3-terminal phosphate cyclase	A8K8K1	RTCA	--	--	--	0.84	0.66	**0.75**	1.05	0.93	**0.99**	--	--	--
Tetratricopeptide repeat protein 9C	Q8N5M4	TTC9C	--	--	--	0.64	0.85	**0.75**	0.97	1.34	**1.16**	--	--	--
Striatin-4	F5GYK2	STRN4	--	--	--	0.72	0.77	**0.74**	0.96	0.98	**0.97**	--	--	--
Synaptobrevin homolog YKT6	Q9UES0	YKT6	--	--	--	0.64	0.83	**0.74**	0.71	0.74	**0.73**	--	--	--
5-AMP-activated protein kinase catalytic subunit alpha-1	Q13131	PRKAA1	--	--	--	0.66	0.81	**0.73**	--	--	--	1.34	0.86	**1.10**
COP9 signalosome complex subunit 1	C9JFE4	GPS1	--	--	--	0.76	0.66	**0.71**	0.72	0.68	**0.70**	--	--	--
Translin	E9PGT1	TSN	--	--	--	0.81	0.59	**0.70**	0.75	0.87	**0.81**	--	--	--
Inosine-5-monophosphate dehydrogenase	A0A024R725	IMPDH1	--	--	--	0.66	0.74	**0.70**	1.62	0.65	**1.14**	--	--	--

S3D Table. *Cont.*

Protein name	Uniprot	Symbol	6 hpi			12 hpi			24 hpi			36 hpi		
			Ad2(L)/ Mock(H)	Mock(L)/ Ad2(H)	Average	Ad2(L)/ Mock(H)	Mock(L)/ Ad2(H)	Average	Ad2(L)/ Mock(H)	Mock(L)/ Ad2(H)	Average	Ad2(L)/ Mock(H)	Mock(L)/ Ad2(H)	Average
Kynurenine--oxoglutarate transaminase 3	B4DW13	CCBL2	--	--	--	0.80	0.59	**0.70**	0.84	0.69	**0.77**	--	--	--
Methylosome protein 50	A0A024R0H7	WDR77	--	--	--	0.69	0.70	**0.69**	0.75	0.88	**0.82**	--	--	--
Programmed cell death protein 6	O75340	PDCD6	--	--	--	0.62	0.70	**0.66**	0.68	0.89	**0.79**	--	--	--
Serine/threonine-protein phosphatase 2B catalytic subunit alpha isoform	A0A0S2Z4B5	PPP3CA	--	--	--	0.71	0.59	**0.65**	0.65	0.73	**0.69**	--	--	--
CAD protein	F8VPD4	CAD	--	--	--	0.59	0.70	**0.64**	--	--	--	0.67	1.14	**0.90**
Zyxin	Q15942	ZYX	--	--	--	0.60	0.68	**0.64**	1.02	0.64	**0.83**	--	--	--
Pyridoxal-dependent decarboxylase domain-containing protein 1	Q6P996	PDXDC1	--	--	--	0.67	0.61	**0.64**	0.85	0.75	**0.80**	--	--	--
D-tyrosyl-tRNA(Tyr) deacylase	Q496C9	DTD1	--	--	--	0.59	0.66	**0.62**	0.74	0.79	**0.77**	--	--	--
Lymphoid-restricted membrane protein	G3V3C2	LRMP	--	--	--	-0.67	-0.88	**-0.78**	--	--	--	-1.45	-0.76	**-1.10**
Tissue-type plasminogen activator	B4DRD3	PLAT	--	--	--	-0.93	-0.63	**-0.78**	-1.02	-1.67	**-1.34**	--	--	--
Versican core protein	A0A024RAL1	CSPG2	--	--	--	-0.68	-0.90	**-0.79**	--	--	--	-1.75	-1.57	**-1.66**
Collagen alpha-2(V) chain	P05997	COL5A2	--	--	--	-1.09	-1.02	**-1.05**	--	--	--	-2.75	-2.16	**-2.45**
Leucine-rich repeat-containing protein 17	Q8N6Y2	LRRC17	--	--	--	-1.75	-0.68	**-1.21**	-1.98	-1.53	**-1.76**	--	--	--
Thymidylate synthase	Q53Y97	TYMS	--	--	--	--	--	--	3.18	2.37	**2.77**	3.02	2.85	**2.93**
Nucleolar and coiled-body phosphoprotein 1	B2RAU8	NOLC1	--	--	--	--	--	--	1.56	3.40	**2.48**	1.36	2.29	**1.83**
Ubiquitin-conjugating enzyme E2 T	A0A024R9A9	UBE2T	--	--	--	--	--	--	2.56	2.23	**2.40**	2.47	1.78	**2.12**
Amidophosphoribosyltransferase	Q53H22	PPAT	--	--	--	--	--	--	2.51	2.06	**2.29**	2.21	1.79	**2.00**
DNA replication licensing factor MCM7	A0A0S2Z4A5	MCM7	--	--	--	--	--	--	2.15	2.31	**2.23**	2.34	2.30	**2.32**
Ribonucleoside-diphosphate reductase	B4DNN4	RRM1	--	--	--	--	--	--	2.06	2.33	**2.20**	2.70	2.45	**2.58**
E3 ubiquitin-protein ligase UHRF1	A0A087WVR3	UHRF1	--	--	--	--	--	--	2.02	2.27	**2.14**	1.31	1.44	**1.38**
4-hydroxyphenylpyruvate dioxygenase-like protein	Q96IR7	HPDL	--	--	--	--	--	--	1.55	2.67	**2.11**	1.85	3.75	**2.80**
Uridine kinase	A0A024R912	UCK2	--	--	--	--	--	--	1.91	2.28	**2.10**	1.68	2.00	**1.84**
DNA helicase	Q53HJ4	MCM3	--	--	--	--	--	--	1.97	2.18	**2.08**	2.48	2.46	**2.47**
Nuclear fragile X mental retardation-interacting protein 2	A1L3A7	NUFIP2	--	--	--	--	--	--	1.84	2.11	**1.97**	1.58	1.51	**1.54**
DNA replication licensing factor MCM4	B4DLA6	MCM4	--	--	--	--	--	--	1.92	1.94	**1.93**	1.98	2.08	**2.03**
Heat shock 70 kDa protein 14	Q0VDF9	HSPA14	--	--	--	--	--	--	1.93	1.83	**1.88**	2.24	0.96	**1.60**
DNA replication licensing factor MCM6	Q14566	MCM6	--	--	--	--	--	--	1.61	2.14	**1.87**	2.00	2.24	**2.12**
Glutathione S-transferase theta-2B	Q6ICJ4	GSTT2	--	--	--	--	--	--	1.91	1.73	**1.82**	1.22	0.95	**1.09**
Acyl-CoA-binding protein	P07108	DBI	--	--	--	--	--	--	1.86	1.78	**1.82**	1.57	1.35	**1.46**
Mini-chromosome maintenance complex-binding protein	A0A0S2Z5P5	MCMBP	--	--	--	--	--	--	1.59	2.04	**1.82**	2.14	1.26	**1.70**
BTB/POZ domain-containing protein KCTD12	B3KY04	KCTD12	--	--	--	--	--	--	1.80	1.78	**1.79**	1.86	1.05	**1.46**
DNA helicase	B3KXZ4	MCM2	--	--	--	--	--	--	1.91	1.66	**1.79**	2.15	1.96	**2.06**
Clustered mitochondria protein homolog	K7EIG1	CLUH	--	--	--	--	--	--	1.55	1.85	**1.70**	1.76	1.61	**1.69**
DNA helicase	Q53FG5	MCM5	--	--	--	--	--	--	1.47	1.88	**1.68**	1.83	2.29	**2.06**
Heat shock 70 kDa protein 4L	E9PDE8	HSPA4L	--	--	--	--	--	--	1.37	1.87	**1.62**	2.10	2.48	**2.29**
Hypoxanthine-guanine phosphoribosyltransferase	Q6LET3	HPRT1	--	--	--	--	--	--	1.55	1.66	**1.60**	1.88	1.55	**1.71**
Glucose 1,6-bisphosphate synthase	Q6PCE3	PGM2L1	--	--	--	--	--	--	2.18	0.99	**1.59**	2.16	0.83	**1.50**
Cytoglobin	Q8WWM9	CYGB	--	--	--	--	--	--	1.51	1.60	**1.56**	1.08	0.94	**1.01**
Zinc finger CCCH domain-containing protein 11A	B4DLG2	ZC3H11A	--	--	--	--	--	--	1.71	1.34	**1.53**	1.63	1.72	**1.68**
3(2),5-bisphosphate nucleotidase 1	V9HWF9	BPNT1	--	--	--	--	--	--	1.61	1.40	**1.50**	1.38	0.91	**1.14**
Adenylate kinase isoenzyme 6	A0A087WVD7	TAF9	--	--	--	--	--	--	1.40	1.60	**1.50**	1.30	1.43	**1.36**
Cyclin-dependent kinase 2	A0A024RB10	CDK2	--	--	--	--	--	--	1.42	1.59	**1.50**	1.59	1.58	**1.58**
Bystin	Q13895	BYSL	--	--	--	--	--	--	1.35	1.63	**1.49**	1.76	1.14	**1.45**
Nucleolar GTP-binding protein 2	Q5T0F3	GNL2	--	--	--	--	--	--	1.41	1.55	**1.48**	1.77	1.80	**1.78**
Prothymosin alpha	B8ZZQ6	PTMA	--	--	--	--	--	--	1.57	1.37	**1.47**	1.68	1.07	**1.37**
Bifunctional coenzyme A synthase	Q13057	COASY	--	--	--	--	--	--	1.57	1.36	**1.47**	1.32	0.63	**0.98**
RNA-binding protein 39	B4DRA0	RBM39	--	--	--	--	--	--	1.40	1.52	**1.46**	2.11	1.72	**1.91**
Proliferating cell nuclear antigen	Q6FHF5	PCNA	--	--	--	--	--	--	1.41	1.49	**1.45**	1.57	1.59	**1.58**

***S3D Table**. Cont.*

Protein name	Uniprot	Symbol	6 hpi			12 hpi			24 hpi			36 hpi		
			Ad2(L)/ Mock(H)	Mock(L)/ Ad2(H)	Average	Ad2(L)/ Mock(H)	Mock(L)/ Ad2(H)	Average	Ad2(L)/ Mock(H)	Mock(L)/ Ad2(H)	Average	Ad2(L)/ Mock(H)	Mock(L)/ Ad2(H)	Average
Multifunctional methyltransferase subunit TRM112-like protein	A0A024R565	HSPC152	--	--	--	--	--	--	1.41	1.45	**1.43**	1.50	1.19	**1.35**
Flap endonuclease 1	Q6FHX6	FEN1	--	--	--	--	--	--	1.29	1.56	**1.43**	1.45	1.39	**1.42**
Eukaryotic translation initiation factor 4B	B4DEP6	EIF4B	--	--	--	--	--	--	1.41	1.44	**1.43**	1.18	2.15	**1.66**
Deoxynucleotidyltransferase terminal-interacting protein 2	Q5QJE6	DNTTIP2	--	--	--	--	--	--	1.54	1.28	**1.41**	0.90	1.34	**1.12**
Opioid growth factor receptor	Q6PK21	OGFR	--	--	--	--	--	--	1.66	1.16	**1.41**	1.38	0.75	**1.07**
Alpha-internexin	Q16352	INA	--	--	--	--	--	--	2.23	0.59	**1.41**	1.37	0.92	**1.15**
Nucleolar RNA helicase 2	Q9NR30	DDX21	--	--	--	--	--	--	1.39	1.40	**1.40**	1.89	1.93	**1.91**
Replication factor C subunit 4	P35249	RFC4	--	--	--	--	--	--	1.38	1.36	**1.37**	1.60	1.02	**1.31**
DNA-directed RNA polymerases I, II, and III subunit RPABC3	C9JLU1	POLR2H	--	--	--	--	--	--	1.32	1.42	**1.37**	1.44	1.21	**1.32**
YTH domain-containing family protein 2	B5BU99	YTHDF2	--	--	--	--	--	--	1.70	0.95	**1.33**	0.82	1.09	**0.95**
Ubiquitin-like protein ISG15	P05161	ISG15	--	--	--	--	--	--	1.14	1.49	**1.31**	1.61	1.37	**1.49**
Heat shock protein 105 kDa	A0A024RDQ0	HSPH1	--	--	--	--	--	--	1.39	1.20	**1.29**	1.17	1.10	**1.13**
YTH domain-containing family protein 3	A0A087X0Q1	YTHDF3	--	--	--	--	--	--	1.15	1.40	**1.27**	2.29	1.62	**1.95**
Phosphomevalonate kinase	Q6FGV9	PMVK	--	--	--	--	--	--	1.22	1.33	**1.27**	1.04	0.73	**0.88**
Probable ATP-dependent RNA helicase DDX20	Q8TDR3	DDX20	--	--	--	--	--	--	1.21	1.34	**1.27**	2.21	1.30	**1.76**
Glutamate-rich WD repeat-containing protein 1	Q8TCJ8	GRWD1	--	--	--	--	--	--	1.41	1.11	**1.26**	1.71	1.38	**1.55**
Histone acetyltransferase type B catalytic subunit	O14929	HAT1	--	--	--	--	--	--	1.22	1.26	**1.24**	1.29	1.44	**1.36**
DnaJ homolog subfamily C member 2	Q99543	DNAJC2	--	--	--	--	--	--	1.02	1.43	**1.22**	2.09	1.53	**1.81**
BRCA2 and CDKN1A-interacting protein	B4E318	BCCIP	--	--	--	--	--	--	0.95	1.49	**1.22**	1.09	1.47	**1.28**
GMP synthase [glutamine-hydrolyzing]	B4DUT7	GMPS	--	--	--	--	--	--	1.15	1.27	**1.21**	1.46	1.21	**1.34**
DnaJ homolog subfamily A member 1	P31689	DNAJA1	--	--	--	--	--	--	1.07	1.35	**1.21**	1.38	1.15	**1.27**
Polyadenylate-binding protein	Q6IQ30	PABPC4	--	--	--	--	--	--	1.14	1.24	**1.19**	0.62	0.91	**0.77**
Pinin	A8K964	PNN	--	--	--	--	--	--	1.00	1.38	**1.19**	1.25	1.36	**1.31**
General transcription factor IIF subunit 2	A0A024RDU9	GTF2F2	--	--	--	--	--	--	0.98	1.40	**1.19**	2.11	1.14	**1.63**
Ran-binding protein 3	B7Z7F3	RANBP3	--	--	--	--	--	--	1.34	1.03	**1.19**	1.85	1.56	**1.70**
Protein phosphatase 1 regulatory subunit 7	A0A140VK83	PPP1R7	--	--	--	--	--	--	1.15	1.22	**1.19**	0.94	0.69	**0.81**
RNA polymerase II subunit A C-terminal domain phosphatase SSU72	Q9NP77	SSU72	--	--	--	--	--	--	0.94	1.42	**1.18**	0.83	1.08	**0.95**
Luc7-like protein 3	Q86Y74	CROP	--	--	--	--	--	--	1.27	1.07	**1.17**	1.31	1.19	**1.25**
DnaJ homolog subfamily C member 7	Q59EH7	DNAJC7	--	--	--	--	--	--	1.09	1.25	**1.17**	1.36	1.03	**1.20**
Hsp70-binding protein 1	Q9NZL4	HSPBP1	--	--	--	--	--	--	1.10	1.22	**1.16**	1.30	0.85	**1.08**
DnaJ homolog subfamily B member 1	Q6FI51	DNAJB1	--	--	--	--	--	--	1.21	1.08	**1.15**	0.94	0.77	**0.85**
Nuclear autoantigenic sperm protein	P49321	NASP	--	--	--	--	--	--	1.17	1.11	**1.14**	1.19	1.15	**1.17**
Protein phosphatase 1G	Q6IAU5	PPM1G	--	--	--	--	--	--	1.01	1.25	**1.13**	1.24	1.23	**1.24**
Mitochondrial ribonuclease P protein 1	Q7L0Y3	TRMT10C	--	--	--	--	--	--	0.87	1.39	**1.13**	1.06	0.97	**1.02**
Nuclear RNA export factor 1	Q9UBU9	NXF1	--	--	--	--	--	--	0.99	1.25	**1.12**	1.25	0.87	**1.06**
Cell division cycle 5-like protein	B4DSH1	CDC5L	--	--	--	--	--	--	0.94	1.26	**1.10**	1.26	1.01	**1.13**
Guanine nucleotide-binding protein-like 3	A0A024R2Z6	GNL3	--	--	--	--	--	--	0.91	1.27	**1.09**	1.08	1.24	**1.16**
Ribonucleoside-diphosphate reductase subunit M2	D6W4Z6	RRM2	--	--	--	--	--	--	0.91	1.27	**1.09**	2.01	2.53	**2.27**
Arfaptin-2	B4DUZ3	ARFIP2	--	--	--	--	--	--	1.02	1.14	**1.08**	1.03	0.73	**0.88**
Melanoma-associated antigen D2	Q5H909	MAGED2	--	--	--	--	--	--	0.95	1.21	**1.08**	1.11	1.20	**1.15**
DNA-directed RNA polymerases I and III subunit RPAC1	E7EQB9	POLR1C	--	--	--	--	--	--	0.96	1.17	**1.07**	0.85	0.69	**0.77**
Regulation of nuclear pre-mRNA domain-containing protein 1B	Q9NQG5	RPRD1B	--	--	--	--	--	--	1.12	1.00	**1.06**	1.32	1.30	**1.31**
Protein FAM50A	Q14320	FAM50A	--	--	--	--	--	--	1.25	0.86	**1.06**	2.31	1.20	**1.75**
Splicing factor 1	A0A024R588	SF1	--	--	--	--	--	--	1.24	0.86	**1.05**	1.44	0.82	**1.13**
Ubiquitin-associated protein 2-like	Q14157	UBAP2L	--	--	--	--	--	--	1.03	1.08	**1.05**	0.97	1.68	**1.32**
Ribosomal L1 domain-containing protein 1	J3QSV6	RSL1D1	--	--	--	--	--	--	1.00	1.10	**1.05**	1.50	0.95	**1.22**
Heat shock cognate 71 kDa protein	V9HW22	HSPA8	--	--	--	--	--	--	0.94	1.15	**1.04**	1.16	1.31	**1.23**
ATP-binding cassette sub-family F member 2	A0A090N7X1	ABCF2	--	--	--	--	--	--	0.85	1.22	**1.04**	1.27	1.10	**1.18**

***S3D Table**. Cont.*

Protein name	Uniprot	Symbol	6 hpi			12 hpi			24 hpi			36 hpi		
			Ad2(L)/ Mock(H)	Mock(L)/ Ad2(H)	Average	Ad2(L)/ Mock(H)	Mock(L)/ Ad2(H)	Average	Ad2(L)/ Mock(H)	Mock(L)/ Ad2(H)	Average	Ad2(L)/ Mock(H)	Mock(L)/ Ad2(H)	Average
	A8K719	CBFB	--	--	--	--	--	--	1.06	1.01	**1.03**	1.16	0.84	**1.00**
	Q14222	EEF1A	--	--	--	--	--	--	1.40	0.67	**1.03**	1.62	0.78	**1.20**
Protein Red	Q9UK43	CSA2	--	--	--	--	--	--	0.74	1.32	**1.03**	1.39	0.76	**1.07**
Developmentally-regulated GTP-binding protein 1	Q9Y295	DRG1	--	--	--	--	--	--	0.95	1.11	**1.03**	1.07	0.93	**1.00**
Probable ATP-dependent RNA helicase DDX5	J3KTA4	DDX5	--	--	--	--	--	--	0.93	1.12	**1.03**	1.76	0.90	**1.33**
Putative RNA-binding protein 15	A0A087WWP	RBM15	--	--	--	--	--	--	1.04	1.01	**1.03**	0.72	1.03	**0.88**
Phosphopantothenate--cysteine ligase	Q9HAB8	PPCS	--	--	--	--	--	--	1.15	0.89	**1.02**	0.95	0.76	**0.85**
Protein RCC2	A5PLK7	RCC2	--	--	--	--	--	--	0.94	1.10	**1.02**	1.34	1.13	**1.23**
Stress-induced-phosphoprotein 1	V9HW72	STIP1	--	--	--	--	--	--	0.96	1.07	**1.02**	0.93	0.95	**0.94**
DCN1-like protein	H0YCN4	DCUN1D5	--	--	--	--	--	--	0.88	1.13	**1.01**	0.85	1.04	**0.94**
ATP-dependent RNA helicase DDX24	G3V529	DDX24	--	--	--	--	--	--	0.65	1.37	**1.01**	2.45	1.51	**1.98**
Pre-rRNA-processing protein TSR1 homolog	Q2NL82	TSR1	--	--	--	--	--	--	0.89	1.12	**1.00**	1.26	1.09	**1.18**
Poly [ADP-ribose] polymerase 1	A0A024R3T8	PARP1	--	--	--	--	--	--	0.88	1.12	**1.00**	1.80	1.10	**1.45**
Gem-associated protein 4	Q9HBB9	GEMIN4	--	--	--	--	--	--	0.91	1.07	**0.99**	1.10	0.88	**0.99**
Myotubularin-related protein 6	Q9Y217	MTMR6	--	--	--	--	--	--	0.90	1.07	**0.99**	0.72	0.75	**0.73**
BUB3-interacting and GLEBS motif-containing protein ZNF207	J3QRS9	ZNF207	--	--	--	--	--	--	0.85	1.11	**0.98**	1.59	1.01	**1.30**
Transcription elongation factor SPT6	Q7KZ85	SUPT6H	--	--	--	--	--	--	0.80	1.15	**0.98**	1.05	1.20	**1.13**
Protein BUD31 homolog	C9JNV2	BUD31	--	--	--	--	--	--	0.84	1.10	**0.97**	0.86	1.03	**0.94**
La-related protein 1	Q6PKG0	LARP1	--	--	--	--	--	--	0.76	1.09	**0.93**	1.67	1.19	**1.43**
Probable ATP-dependent RNA helicase DDX47	A0A024RAS3	DDX47	--	--	--	--	--	--	0.68	1.17	**0.92**	1.08	1.03	**1.05**
Translation initiation factor eIF-2B subunit beta	Q53XC2	EIF2B2	--	--	--	--	--	--	0.77	1.07	**0.92**	1.12	0.78	**0.95**
mRNA turnover protein 4 homolog	A0A024RAE1	MRTO4	--	--	--	--	--	--	0.90	0.94	**0.92**	0.87	1.21	**1.04**
ATP-binding cassette sub-family F member 1	Q2L6I2	ABCF1	--	--	--	--	--	--	0.71	1.12	**0.92**	0.87	1.22	**1.04**
RNA-binding protein 14	A0A0S2Z4Z0	RBM14	--	--	--	--	--	--	0.89	0.91	**0.90**	1.77	0.80	**1.28**
Probable ATP-dependent RNA helicase DDX6	B2R858	DDX6	--	--	--	--	--	--	0.68	1.13	**0.90**	0.79	0.96	**0.88**
Peptidyl-prolyl cis-trans isomerase	B2R6X6	PPIF	--	--	--	--	--	--	0.69	1.09	**0.89**	0.99	1.10	**1.05**
Eukaryotic translation initiation factor 3 subunit D	O15371	EIF3D	--	--	--	--	--	--	0.75	1.03	**0.89**	1.56	1.22	**1.39**
Heterogeneous nuclear ribonucleoprotein F	A0A024R7T3	HNRPF	--	--	--	--	--	--	0.64	1.14	**0.89**	1.04	0.92	**0.98**
Insulin-degrading enzyme	P14735	IDE	--	--	--	--	--	--	0.89	0.89	**0.89**	1.13	0.80	**0.97**
Bcl-2-associated transcription factor 1	Q6DCA8	BCLAF1	--	--	--	--	--	--	0.87	0.91	**0.89**	1.11	1.81	**1.46**
RNA-binding protein FUS	Q59H57	FUS	--	--	--	--	--	--	0.77	1.00	**0.89**	0.69	0.76	**0.73**
THUMP domain-containing protein 1	A0A024R388	THUMPD1	--	--	--	--	--	--	0.75	1.01	**0.88**	0.72	0.79	**0.76**
Inosine-5-monophosphate dehydrogenase 2	P12268	IMPDH2	--	--	--	--	--	--	0.75	1.01	**0.88**	1.17	1.04	**1.10**
FACT complex subunit SPT16	Q9Y5B9	SUPT16H	--	--	--	--	--	--	0.84	0.91	**0.87**	1.20	1.30	**1.25**
Lamina-associated polypeptide 2, isoforms beta/gamma	A0A024RBE7	TMPO	--	--	--	--	--	--	1.02	0.73	**0.87**	0.81	1.11	**0.96**
A-kinase anchor protein 12	Q02952	AKAP12	--	--	--	--	--	--	1.16	0.59	**0.87**	0.93	0.65	**0.79**
Cullin-associated NEDD8-dissociated protein 1	A8K8U1	CAND1	--	--	--	--	--	--	0.89	0.85	**0.87**	1.13	0.77	**0.95**
40S ribosomal protein S6	Q96DV6	RPS6	--	--	--	--	--	--	0.69	1.01	**0.85**	1.07	1.09	**1.08**
Oxysterol-binding protein	B3KW33	OSBPL9	--	--	--	--	--	--	0.75	0.95	**0.85**	0.80	0.70	**0.75**
Replication protein A 70 kDa DNA-binding subunit	P27694	RPA1	--	--	--	--	--	--	0.74	0.95	**0.84**	1.59	0.88	**1.23**
Interferon-induced, double-stranded RNA-activated protein kinase	B7ZKK7	EIF2AK2	--	--	--	--	--	--	0.67	1.01	**0.84**	1.09	0.88	**0.98**
Transcription elongation factor B polypeptide 2	Q15370	TCEB2	--	--	--	--	--	--	0.72	0.95	**0.84**	0.75	0.74	**0.74**
Serrate RNA effector molecule homolog	Q9BXP5	SRRT	--	--	--	--	--	--	0.79	0.87	**0.83**	1.02	1.10	**1.06**
Glycylpeptide N-tetradecanoyltransferase	Q96HI4	NMT1	--	--	--	--	--	--	0.70	0.96	**0.83**	1.00	0.63	**0.81**
Microtubule-associated protein	E7EVA0	MAP4	--	--	--	--	--	--	0.98	0.67	**0.83**	1.38	0.73	**1.06**
ATP-dependent RNA helicase DDX3X	A0A0D9SFB3	DDX3X	--	--	--	--	--	--	0.61	0.99	**0.80**	1.19	1.08	**1.13**
SRSF protein kinase 1	A0A024RCU9	SRPK1	--	--	--	--	--	--	0.69	0.91	**0.80**	1.04	0.95	**0.99**
Regulator of nonsense transcripts 1	A0A024R7L5	UPF1	--	--	--	--	--	--	0.80	0.79	**0.80**	0.97	1.02	**0.99**

***S3D Table**. Cont.*

Protein name	Uniprot	Symbol	6 hpi			12 hpi			24 hpi			36 hpi		
			Ad2(L)/ Mock(H)	Mock(L)/ Ad2(H)	Average	Ad2(L)/ Mock(H)	Mock(L)/ Ad2(H)	Average	Ad2(L)/ Mock(H)	Mock(L)/ Ad2(H)	Average	Ad2(L)/ Mock(H)	Mock(L)/ Ad2(H)	Average
60S ribosomal protein L36	Q9Y3U8	RPL36	--	--	--	--	--	--	0.69	0.90	**0.80**	1.00	0.74	**0.87**
Activated RNA polymerase II transcriptional coactivator p15	Q6IBA2	PC4	--	--	--	--	--	--	0.85	0.74	**0.79**	1.12	0.60	**0.86**
40S ribosomal protein S27	P42677	RPS27	--	--	--	--	--	--	0.78	0.78	**0.78**	1.46	0.59	**1.03**
T-complex protein 1 subunit alpha	P17987	TCP1	--	--	--	--	--	--	0.75	0.81	**0.78**	1.01	1.31	**1.16**
FACT complex subunit SSRP1	A0A024R4Z6	SSRP1	--	--	--	--	--	--	0.66	0.89	**0.78**	0.82	1.14	**0.98**
Heat shock 70 kDa protein 4	P34932	HSPA4	--	--	--	--	--	--	0.72	0.83	**0.78**	0.84	0.75	**0.80**
SAP domain-containing ribonucleoprotein	P82979	SARNP	--	--	--	--	--	--	0.72	0.83	**0.78**	0.97	1.00	**0.98**
Protein arginine N-methyltransferase 5	O14744	PRMT5	--	--	--	--	--	--	0.75	0.78	**0.77**	0.94	0.62	**0.78**
Cullin-1	A0A090N7U0	CUL1	--	--	--	--	--	--	0.79	0.75	**0.77**	1.02	0.77	**0.90**
Protein argonaute-2	Q9UKV8	EIF2C2	--	--	--	--	--	--	0.75	0.78	**0.76**	0.95	0.77	**0.86**
Transportin-3	B2R6H7	TNPO3	--	--	--	--	--	--	0.65	0.86	**0.75**	0.83	0.75	**0.79**
40S ribosomal protein S28	B2R4R9	RPS28	--	--	--	--	--	--	0.66	0.84	**0.75**	0.89	0.61	**0.75**
Eukaryotic translation initiation factor 3 subunit C	B4DVQ5	EIF3C	--	--	--	--	--	--	0.65	0.84	**0.74**	1.33	1.04	**1.19**
Protein SCAF8	B7Z876	SCAF8	--	--	--	--	--	--	0.84	0.64	**0.74**	1.68	0.91	**1.29**
Putative RNA-binding protein Luc7-like 2	Q9Y383	LUC7L2	--	--	--	--	--	--	0.67	0.81	**0.74**	0.76	0.70	**0.73**
PERQ amino acid-rich with GYF domain-containing protein 2	A0A024R4A5	TNRC15	--	--	--	--	--	--	0.74	0.75	**0.74**	1.01	0.78	**0.89**
Serine/arginine-rich splicing factor 7	A0A0B4J1Z1	SRSF7	--	--	--	--	--	--	0.79	0.69	**0.74**	0.86	0.70	**0.78**
Importin subunit alpha	Q7Z726	KPNA2	--	--	--	--	--	--	0.63	0.84	**0.73**	0.85	0.85	**0.85**
Transcription elongation regulator 1	O14776	TCERG1	--	--	--	--	--	--	0.67	0.78	**0.72**	0.93	1.09	**1.01**
40S ribosomal protein S21	Q8WVC2	RPS21	--	--	--	--	--	--	0.61	0.84	**0.72**	0.97	0.77	**0.87**
Serine-threonine kinase receptor-associated protein	Q9Y3F4	STRAP	--	--	--	--	--	--	0.68	0.76	**0.72**	0.72	0.65	**0.68**
Eukaryotic translation initiation factor 2A	Q9BY44	EIF2A	--	--	--	--	--	--	0.70	0.73	**0.72**	1.20	0.85	**1.03**
tRNA (cytosine(34)-C(5))-methyltransferase	Q08J23	NSUN2	--	--	--	--	--	--	0.72	0.70	**0.71**	1.25	1.09	**1.17**
Serine/arginine repetitive matrix protein 2	A0A140VK53	SRRM2	--	--	--	--	--	--	0.60	0.80	**0.70**	1.17	0.83	**1.00**
RNA-binding protein PNO1	B2R823	PNO1	--	--	--	--	--	--	0.71	0.69	**0.70**	0.90	0.62	**0.76**
Eukaryotic translation initiation factor 2 subunit 1	Q53XC0	EIF2S1	--	--	--	--	--	--	0.67	0.71	**0.69**	0.82	0.65	**0.73**
60S ribosomal protein L23	Q9BTQ7	RPL23	--	--	--	--	--	--	0.61	0.76	**0.69**	0.78	0.68	**0.73**
Cullin-3	Q53S54	CUL3	--	--	--	--	--	--	0.61	0.74	**0.67**	0.94	0.62	**0.78**
Eukaryotic initiation factor 4A-III	A0A024R8W0	DDX48	--	--	--	--	--	--	0.70	0.62	**0.66**	0.67	0.71	**0.69**
Ribosome biogenesis protein BRX1 homolog	Q8TDN6	BRIX1	--	--	--	--	--	--	0.69	0.59	**0.64**	0.90	0.67	**0.78**
Prolyl 4-hydroxylase subunit alpha-2	Q05DA4	P4HA2	--	--	--	--	--	--	-0.59	-0.63	**-0.61**	-0.73	-0.92	**-0.83**
Keratin, type I cytoskeletal 18	F8VZY9	KRT18	--	--	--	--	--	--	-0.64	-0.62	**-0.63**	-1.19	-0.78	**-0.98**
Integrin alpha-5	A8K6A5	ITGA5	--	--	--	--	--	--	-0.68	-0.59	**-0.64**	-0.59	-0.64	**-0.61**
Delta-sarcoglycan	Q92629	SGCD	--	--	--	--	--	--	-0.64	-0.65	**-0.65**	-0.87	-1.16	**-1.01**
Catenin beta-1	B5BU28	CTNNB1	--	--	--	--	--	--	-0.68	-0.63	**-0.65**	-0.61	-0.79	**-0.70**
Ras-related protein R-Ras2	P62070	RRAS2	--	--	--	--	--	--	-0.68	-0.68	**-0.68**	-0.76	-0.80	**-0.78**
Ceroid-lipofuscinosis neuronal protein 5	B4E1V6	CLN5	--	--	--	--	--	--	-0.63	-0.78	**-0.71**	-0.81	-0.93	**-0.87**
Golgi integral membrane protein 4	O00461	GOLIM4	--	--	--	--	--	--	-0.85	-0.59	**-0.72**	-1.68	-1.38	**-1.53**
Tetraspanin	E9PMR4	CD151	--	--	--	--	--	--	-0.84	-0.64	**-0.74**	-1.56	-0.86	**-1.21**
Ras-related protein Ral-B	A0A024RAG3	RALB	--	--	--	--	--	--	-0.76	-0.74	**-0.75**	-1.03	-0.90	**-0.97**
Protein wntless homolog	Q5T9L3	WLS	--	--	--	--	--	--	-0.70	-0.82	**-0.76**	-1.12	-1.28	**-1.20**
Sulfhydryl oxidase 1	O00391	QSOX1	--	--	--	--	--	--	-0.77	-0.76	**-0.76**	-1.53	-1.77	**-1.65**
Dystonin	Q03001	DST	--	--	--	--	--	--	-0.78	-0.77	**-0.77**	-0.91	-0.95	**-0.93**
Myoferlin	Q9NZM1	MYOF	--	--	--	--	--	--	-0.88	-0.70	**-0.79**	-0.80	-0.77	**-0.78**
Neuroplastin	Q9Y639	NPTN	--	--	--	--	--	--	-0.83	-0.77	**-0.80**	-0.90	-1.10	**-1.00**
Ras-related protein Rab-3B	A8K6I8	RAB3B	--	--	--	--	--	--	-0.79	-0.81	**-0.80**	-1.28	-1.25	**-1.26**
Receptor protein-tyrosine kinase	B4DSE0	EPHB2	--	--	--	--	--	--	-1.03	-0.60	**-0.81**	-1.12	-0.79	**-0.95**
Calcium-transporting ATPase	A0A024R968	ATP2B4	--	--	--	--	--	--	-0.72	-0.92	**-0.82**	-0.82	-0.87	**-0.85**

***S3D Table**. Cont.*

Protein name	Uniprot	Symbol	6 hpi			12 hpi			24 hpi			36 hpi		
			Ad2(L)/ Mock(H)	Mock(L)/ Ad2(H)	Average	Ad2(L)/ Mock(H)	Mock(L)/ Ad2(H)	Average	Ad2(L)/ Mock(H)	Mock(L)/ Ad2(H)	Average	Ad2(L)/ Mock(H)	Mock(L)/ Ad2(H)	Average
Collagen alpha-3(VI) chain	D9ZGF2	COL6A3	--	--	--	--	--	--	-0.99	-0.68	**-0.84**	-1.02	-0.99	**-1.01**
Collagen alpha-2(VI) chain	P12110	COL6A2	--	--	--	--	--	--	-1.06	-0.65	**-0.85**	-1.02	-1.04	**-1.03**
Ras-related protein Rab-3D	A0A024R7G2	RAB3D	--	--	--	--	--	--	-0.90	-0.82	**-0.86**	-1.45	-1.27	**-1.36**
Histone H1x	Q92522	H1FX	--	--	--	--	--	--	-0.75	-0.99	**-0.87**	-1.38	-1.58	**-1.48**
Transforming growth factor-beta-induced protein ig-h3	A0A0S2Z4Q2	TGFBI	--	--	--	--	--	--	-1.06	-0.68	**-0.87**	-1.43	-1.12	**-1.28**
High mobility group protein HMGI-C	F5H2U8	HMGA2	--	--	--	--	--	--	-0.77	-0.98	**-0.87**	-0.87	-1.55	**-1.21**
Anion exchange protein	A0A0A0MST8	SLC4A7	--	--	--	--	--	--	-1.06	-0.70	**-0.88**	-0.79	-0.78	**-0.79**
	B4E3P1		--	--	--	--	--	--	-1.07	-0.75	**-0.91**	-1.39	-1.11	**-1.25**
Thy-1 membrane glycoprotein	Q59GA0	THY1	--	--	--	--	--	--	-0.98	-0.88	**-0.93**	-1.83	-1.56	**-1.69**
Tenascin	A0A024R884	TNC	--	--	--	--	--	--	-0.81	-1.05	**-0.93**	-1.53	-0.76	**-1.15**
Ras-related protein R-Ras	A0A024QZF2	RRAS	--	--	--	--	--	--	-0.93	-0.94	**-0.93**	-1.04	-1.07	**-1.06**
Collagen alpha-1(VI) chain	A0A087X0S5	COL6A1	--	--	--	--	--	--	-1.16	-0.71	**-0.94**	-1.07	-1.02	**-1.05**
Matrix metalloproteinase-14	K4RH61	MMP14	--	--	--	--	--	--	-1.04	-0.84	**-0.94**	-1.56	-1.42	**-1.49**
Fibronectin type III domain-containing protein 3B	Q53EP0	FNDC3B	--	--	--	--	--	--	-1.13	-0.77	**-0.95**	-1.59	-1.09	**-1.34**
	A0A024R5W6	TPM1	--	--	--	--	--	--	-0.96	-0.96	**-0.96**	-1.26	-1.47	**-1.37**
Junction plakoglobin	A0A0S2Z487	JUP	--	--	--	--	--	--	-0.95	-0.98	**-0.97**	-1.24	-1.71	**-1.48**
Reversion-inducing cysteine-rich protein with Kazal motifs	B2RCE6	RECK	--	--	--	--	--	--	-1.05	-0.89	**-0.97**	-1.13	-1.62	**-1.37**
Filamin A-interacting protein 1-like	Q4L180	FILIP1L	--	--	--	--	--	--	-1.29	-0.70	**-0.99**	-0.78	-0.67	**-0.72**
Protein-lysine 6-oxidase	Q96PQ9	LOX	--	--	--	--	--	--	-1.14	-0.86	**-1.00**	-1.39	-1.32	**-1.36**
Prolyl 3-hydroxylase 2	Q8IVL5	LEPREL1	--	--	--	--	--	--	-0.79	-1.22	**-1.00**	-1.20	-1.14	**-1.17**
Poliovirus receptor	B3KSS4	PVR	--	--	--	--	--	--	-1.13	-0.90	**-1.01**	-1.30	-1.21	**-1.26**
Cadherin-13	B7Z9B1	CDH13	--	--	--	--	--	--	-1.26	-0.79	**-1.02**	-2.37	-1.38	**-1.88**
Endosialin	Q9HCU0	CD248	--	--	--	--	--	--	-1.02	-1.02	**-1.02**	-1.87	-2.93	**-2.40**
CD59 glycoprotein	E9PNW4	CD59	--	--	--	--	--	--	-1.13	-0.98	**-1.05**	-1.75	-1.73	**-1.74**
Uveal autoantigen with coiled-coil domains and ankyrin repeats	F5H2B9	UACA	--	--	--	--	--	--	-1.23	-0.94	**-1.08**	-1.83	-1.31	**-1.57**
	E7ESP4	ITGA2	--	--	--	--	--	--	-1.43	-0.78	**-1.10**	-1.03	-1.01	**-1.02**
Integrin alpha-V	A5YM53	ITGAV	--	--	--	--	--	--	-1.07	-1.13	**-1.10**	-1.39	-1.25	**-1.32**
Histone H1.3	P16402	HIST1H1D	--	--	--	--	--	--	-1.09	-1.14	**-1.12**	-1.16	-1.79	**-1.48**
Integrin alpha-2	P17301	ITGA2	--	--	--	--	--	--	-1.07	-1.25	**-1.16**	-1.41	-1.38	**-1.39**
Thrombospondin type-1 domain-containing protein 4	Q6ZMP0	THSD4	--	--	--	--	--	--	-1.25	-1.10	**-1.17**	-0.67	-0.85	**-0.76**
DNA topoisomerase 2-alpha	P11388	TOP2A	--	--	--	--	--	--	-0.75	-1.63	**-1.19**	-1.09	-0.90	**-0.99**
EGF-like repeat and discoidin I-like domain-containing protein 3	O43854	EDIL3	--	--	--	--	--	--	-1.80	-0.60	**-1.20**	-1.49	-1.59	**-1.54**
Receptor protein-tyrosine kinase	A0A024QZA8	EPHA2	--	--	--	--	--	--	-1.73	-0.68	**-1.20**	-1.56	-1.48	**-1.52**
C-type mannose receptor 2	Q9UBG0	MRC2	--	--	--	--	--	--	-0.94	-1.53	**-1.24**	-2.09	-1.90	**-1.99**
Integrin alpha-3	B4E0H8	ITGA3	--	--	--	--	--	--	-1.54	-1.12	**-1.33**	-1.85	-1.36	**-1.61**
Collagen triple helix repeat-containing protein 1	Q96CG8	CTHRC1	--	--	--	--	--	--	-1.68	-1.01	**-1.35**	-2.07	-1.74	**-1.91**
Endoglin	Q5T9B9	ENG	--	--	--	--	--	--	-1.24	-1.49	**-1.36**	-1.25	-1.56	**-1.41**
Sodium-coupled neutral amino acid transporter 2	A8K6H9	SLC38A2	--	--	--	--	--	--	-1.46	-1.31	**-1.38**	-1.57	-1.33	**-1.45**
Trophoblast glycoprotein	Q13641	TPBG	--	--	--	--	--	--	-1.49	-1.38	**-1.44**	-2.13	-1.79	**-1.96**
Cadherin-6	P55285	CDH6	--	--	--	--	--	--	-1.19	-1.70	**-1.44**	-1.60	-2.01	**-1.81**
Cell surface glycoprotein MUC18	A0A024R3I5	MCAM	--	--	--	--	--	--	-1.44	-1.90	**-1.67**	-1.22	-1.76	**-1.49**
Insulin-like growth factor-binding protein 5	A0A024R433	IGFBP5	--	--	--	--	--	--	-1.87	-1.48	**-1.68**	-2.53	-1.91	**-2.22**
Vasorin	Q6EMK4	VASN	--	--	--	--	--	--	-1.51	-1.93	**-1.72**	-1.40	-1.57	**-1.48**
Neuropilin-1	Q59F20	NRP1	--	--	--	--	--	--	-2.45	-1.12	**-1.79**	-2.16	-0.98	**-1.57**
Chondroitin sulfate proteoglycan 4	Q6UVK1	CSPG4	--	--	--	--	--	--	-2.17	-1.87	**-2.02**	-3.39	-0.89	**-2.14**
Platelet-derived growth factor receptor beta	P09619	PDGFRB	--	--	--	--	--	--	-2.66	-1.68	**-2.17**	-2.94	-1.88	**-2.41**
CD166 antigen	Q13740	ALCAM	--	--	--	--	--	--	-2.19	-2.58	**-2.39**	-3.27	-2.70	**-2.99**
Receptor protein-tyrosine kinase	Q2TTR7	EGFR	--	--	--	--	--	--	-2.33	-2.74	**-2.53**	-3.00	-2.05	**-2.52**

S3D Table. *Cont.*

Protein name	Uniprot	Symbol	6 hpi			12 hpi			24 hpi			36 hpi		
			Ad2(L)/ Mock(H)	Mock(L)/ Ad2(H)	Average	Ad2(L)/ Mock(H)	Mock(L)/ Ad2(H)	Average	Ad2(L)/ Mock(H)	Mock(L)/ Ad2(H)	Average	Ad2(L)/ Mock(H)	Mock(L)/ Ad2(H)	Average
Interferon-induced guanylate-binding protein 1	Q5D1D5	GBP1	1.65	1.45	**1.55**	1.54	1.02	**1.28**	1.11	1.26	**1.18**	--	--	--
Aldo-keto reductase family 1 member C1	Q04828	AKR1C1	1.61	1.22	**1.42**	2.65	1.10	**1.87**	1.25	0.84	**1.04**	--	--	--
Annexin	A0A0S2Z377	ANXA6	1.37	1.45	**1.41**	1.36	1.08	**1.22**	1.16	1.00	**1.08**	--	--	--
Alpha/beta hydrolase domain-containing protein 14B	B4DNR3	ABHD14B	1.14	1.66	**1.40**	1.31	1.48	**1.40**	1.65	1.30	**1.47**	--	--	--
Tubulin beta-2B chain	Q9BVA1	TUBB2B	1.18	1.61	**1.40**	1.43	1.53	**1.48**	0.94	0.79	**0.86**	--	--	--
Asparagine synthetase [glutamine-hydrolyzing]	P08243	ASNS	1.19	1.45	**1.32**	1.31	1.16	**1.23**	1.29	1.21	**1.25**	--	--	--
ADP-ribosylation factor-like protein 3	P36405	ARL3	1.25	1.37	**1.31**	1.72	1.40	**1.56**	1.54	1.56	**1.55**	--	--	--
Tubulin alpha-1A chain	Q71U36	TUBA1A	1.12	1.49	**1.31**	1.51	1.21	**1.36**	0.81	0.86	**0.83**	--	--	--
	Q8IWP6		1.01	1.60	**1.30**	1.56	1.49	**1.53**	0.84	0.85	**0.85**	--	--	--
Pyruvate kinase	V9HWB8	PKM	1.14	1.32	**1.23**	1.38	1.08	**1.23**	0.94	0.80	**0.87**	--	--	--
Glutathione S-transferase Mu 2	Q0D2I8	GSTM2	1.19	1.25	**1.22**	--	--	--	1.22	1.32	**1.27**	1.45	0.85	1.15
Sorcin	C9J0K6	SRI	0.97	1.42	**1.20**	1.28	1.19	**1.23**	1.06	1.14	**1.10**	--	--	--
Tubulin beta chain	Q5JP53	TUBB	1.01	1.38	**1.19**	1.39	1.32	**1.36**	0.98	1.13	**1.05**	--	--	--
Selenium-binding protein 1	V9HWG1	SELENBP1	1.14	1.21	**1.17**	2.00	1.08	**1.54**	1.28	1.13	**1.21**	--	--	--
Pantothenate kinase 4	B4DHW9	PANK4	0.93	1.38	**1.15**	0.95	1.07	**1.01**	0.83	1.08	**0.95**	--	--	--
Phosphoserine phosphatase	C9JBI3	PSPH	1.11	1.19	**1.15**	1.35	1.13	**1.24**	1.31	0.66	**0.99**	--	--	--
Dual specificity protein phosphatase 3	B5BUI8	DUSP3	0.69	1.60	**1.15**	0.93	1.20	**1.06**	0.82	1.10	**0.96**	--	--	--
Tyrosine--tRNA ligase, cytoplasmic	A0A0S2Z4R1	YARS	1.16	1.13	**1.15**	1.12	1.12	**1.12**	0.69	1.04	**0.87**	--	--	--
Gelsolin	B7Z992	GSN	1.15	1.14	**1.14**	1.49	0.80	**1.14**	1.22	0.83	**1.03**	--	--	--
Aconitate hydratase	V9HWB7	ACO1	1.26	0.99	**1.13**	1.79	0.85	**1.32**	1.18	0.91	**1.04**	--	--	--
Calcium-regulated heat stable protein 1	Q9Y2V2	CARHSP1	1.18	1.07	**1.12**	1.54	0.96	**1.25**	--	--	--	1.72	0.79	1.26
Dihydropyrimidinase-related protein 3	Q6DEN2	DPYSL3	1.11	1.13	**1.12**	1.25	1.13	**1.19**	0.94	1.14	**1.04**	--	--	--
Chloride intracellular channel protein	Q5SRT3	CLIC1	0.90	1.33	**1.12**	0.95	1.30	**1.12**	0.81	0.78	**0.80**	--	--	--
Ubiquitin-conjugating enzyme E2 K	B4DIZ2	UBE2K	1.09	1.14	**1.12**	1.28	1.06	**1.17**	1.39	1.34	**1.36**	--	--	--
Stathmin	A2A2D0	STMN1	1.16	1.07	**1.12**	1.44	1.04	**1.24**	1.06	0.94	**1.00**	--	--	--
Annexin	Q5TZZ9	ANXA1	0.93	1.29	**1.11**	1.15	1.26	**1.21**	1.04	1.19	**1.12**	--	--	--
S-methyl-5-thioadenosine phosphorylase	B4DUC8	MTAP	0.96	1.25	**1.11**	1.23	1.03	**1.13**	1.03	1.01	**1.02**	--	--	--
Ubiquitin carboxyl-terminal hydrolase isozyme L3	A0A140VJZ4	UCHL3	0.88	1.33	**1.11**	1.19	1.05	**1.12**	1.25	0.82	**1.04**	--	--	--
TIP41-like protein	O75663	TIPRL	1.07	1.12	**1.09**	1.42	0.90	**1.16**	1.38	1.14	**1.26**	--	--	--
Puromycin-sensitive aminopeptidase	E9PLK3	NPEPPS	0.96	1.23	**1.09**	1.34	0.94	**1.14**	1.08	0.90	**0.99**	--	--	--
Argininosuccinate lyase	A0A024RDL8	ASL	1.25	0.92	**1.09**	1.58	0.76	**1.17**	1.13	0.85	**0.99**	--	--	--
Peroxiredoxin-1	Q06830	PRDX1	0.92	1.25	**1.08**	1.13	1.16	**1.14**	0.97	0.92	**0.94**	--	--	--
Cytosolic purine 5-nucleotidase	A8K6K2	NT5C2	0.92	1.23	**1.08**	0.67	1.24	**0.95**	0.98	1.16	**1.07**	--	--	--
cGMP-specific 3,5-cyclic phosphodiesterase	I6NLS4	PDE5A	0.97	1.17	**1.07**	1.05	1.02	**1.04**	0.73	0.59	**0.66**	--	--	--
Acylamino-acid-releasing enzyme	A0A024R2U9	APEH	1.26	0.88	**1.07**	1.04	0.99	**1.01**	0.75	0.69	**0.72**	--	--	--
Dihydropyrimidinase-related protein 2	Q59GB4	DPYSL2	0.86	1.25	**1.06**	1.28	1.01	**1.15**	0.95	1.13	**1.04**	--	--	--
Spermine synthase	P52788	SMS	0.96	1.12	**1.04**	1.25	0.95	**1.10**	1.18	0.89	**1.03**	--	--	--
D-3-phosphoglycerate dehydrogenase	O43175	PHGDH	0.88	1.20	**1.04**	1.20	1.08	**1.14**	0.90	0.64	**0.77**	--	--	--
Rho GTPase-activating protein 1	Q07960	ARHGAP1	0.84	1.23	**1.03**	1.21	1.09	**1.15**	0.96	0.97	**0.97**	--	--	--
Macrophage-capping protein	B4DU58	CAPG	0.81	1.25	**1.03**	1.13	1.38	**1.26**	1.08	1.43	**1.26**	--	--	--
3-hydroxybutyrate dehydrogenase type 2	A0A024RDG9	BDH2	0.65	1.41	**1.03**	1.44	1.10	**1.27**	1.41	0.65	**1.03**	--	--	--
Mannose-1-phosphate guanyltransferase beta	A0A024R2X1	GMPPB	1.00	1.04	**1.02**	1.08	1.00	**1.04**	1.16	0.82	**0.99**	--	--	--
Ubiquitin-conjugating enzyme E2 D3	A0A024RDH2	UBE2D3	0.65	1.40	**1.02**	--	--	--	1.38	1.72	**1.55**	1.55	1.11	1.33
Programmed cell death 6-interacting protein	Q8WUM4	PDCD6IP	0.84	1.20	**1.02**	0.95	0.97	**0.96**	0.74	0.82	**0.78**	--	--	--
UDP-glucose 6-dehydrogenase	O60701	UGDH	0.96	1.06	**1.01**	1.14	0.90	**1.02**	0.80	0.59	**0.69**	--	--	--
Serine/threonine-protein phosphatase 2A 56 kDa regulatory subunit delta	B4DSD7	PPP2R5D	0.95	1.04	**1.00**	1.06	0.75	**0.90**	1.11	1.07	**1.09**	--	--	--
Sorting nexin-12	Q3SYF1	SNX12	0.92	1.07	**0.99**	1.07	1.16	**1.11**	0.92	1.15	**1.03**	--	--	--
Calpain-1 catalytic subunit	A0A024R5A3	CAPN1	0.75	1.23	**0.99**	1.09	0.94	1.02	1.03	0.93	**0.98**	--	--	--

***S3D Table*. Cont.**

Protein name	Uniprot	Symbol	6 hpi			12 hpi			24 hpi			36 hpi		
			Ad2(L)/ Mock(H)	Mock(L)/ Ad2(H)	Average	Ad2(L)/ Mock(H)	Mock(L)/ Ad2(H)	Average	Ad2(L)/ Mock(H)	Mock(L)/ Ad2(H)	Average	Ad2(L)/ Mock(H)	Mock(L)/ Ad2(H)	Average
Exportin-T	A8KA19	XPOT	1.12	0.85	**0.99**	1.34	0.78	**1.06**	1.41	0.97	**1.19**	--	--	--
Nardilysin	B1AKJ5	NRD1	0.91	1.04	**0.97**	1.34	1.01	**1.18**	0.86	0.92	**0.89**	--	--	--
14-3-3 protein theta	P27348	YWHAQ	0.88	1.06	**0.97**	1.10	1.01	**1.06**	0.95	0.99	**0.97**	--	--	--
Importin-9	Q96P70	IPO9	0.85	1.08	**0.96**	1.10	0.94	**1.02**	0.70	1.06	**0.88**	--	--	--
Calpain small subunit 1	A0A0C4DGQ5	CAPNS1	0.60	1.33	**0.96**	0.85	1.34	**1.09**	1.07	0.86	**0.97**	--	--	--
Ubiquitin-like modifier-activating enzyme 6	A0A024RDB0	UBE1L2	0.95	0.97	**0.96**	0.98	0.74	**0.86**	0.64	0.75	**0.69**	--	--	--
Thioredoxin reductase 1, cytoplasmic	E9PIR7	TXNRD1	0.65	1.26	**0.96**	0.92	1.23	**1.07**	0.78	0.91	**0.84**	--	--	--
Isoamyl acetate-hydrolyzing esterase 1 homolog	H7C5G1	IAH1	0.96	0.95	**0.95**	1.02	0.88	**0.95**	1.10	0.59	**0.85**	--	--	--
Ubiquitin carboxyl-terminal hydrolase 5	A0A140VJZ1	USP5	0.96	0.95	**0.95**	1.26	0.68	**0.97**	0.94	0.79	**0.86**	--	--	--
Calpain-2 catalytic subunit	Q59EF6	CAPN2	0.70	1.21	**0.95**	1.00	1.09	**1.05**	0.96	0.99	**0.97**	--	--	--
Serine/threonine-protein kinase OSR1	A0A024R2M7	OXSR1	0.74	1.15	**0.94**	0.98	0.93	**0.96**	1.13	1.01	**1.07**	--	--	--
Galactokinase	V9HWE7	GALK1	1.14	0.72	**0.93**	--	--	--	1.71	1.04	**1.37**	2.09	1.05	**1.57**
Histidine--tRNA ligase, cytoplasmic	P12081	HARS	0.89	0.96	**0.92**	1.13	0.98	**1.06**	0.86	0.91	**0.89**	--	--	--
14-3-3 protein zeta/delta	D0PNI1	YWHAZ	0.76	1.08	**0.92**	0.94	0.99	**0.96**	0.77	0.67	**0.72**	--	--	--
Probable E3 ubiquitin-protein ligase HERC4	A0A024QZN8	HERC4	0.88	0.95	**0.91**	1.30	1.00	**1.15**	0.82	0.63	**0.72**	--	--	--
Tax1-binding protein 3	O14907	TAX1BP3	0.74	1.09	**0.91**	1.01	1.12	**1.07**	0.73	1.00	**0.86**	--	--	--
Vinculin	A0A024QZN4	VCL	0.79	1.03	**0.91**	0.97	0.82	**0.90**	0.75	0.81	**0.78**	--	--	--
Omega-amidase NIT2	V9HW91	NIT2	0.87	0.94	**0.90**	1.18	0.71	**0.95**	1.02	0.75	**0.89**	--	--	--
Cysteine--tRNA ligase, cytoplasmic	A0A024RCG3	CARS	0.79	1.00	**0.89**	0.96	0.79	**0.88**	0.62	0.62	**0.62**	--	--	--
Phosphatidylinositol transfer protein alpha isoform	V9HWC5	PITPNA	0.60	1.17	**0.89**	--	--	--	1.45	1.14	**1.30**	1.13	1.03	**1.08**
Proteasome activator complex subunit 1	A0A0K0K1L8	PSME1	0.73	1.04	**0.88**	0.85	1.00	**0.92**	0.84	0.66	**0.75**	--	--	--
Proteasome activator complex subunit 2	Q86SZ7	PSME2	0.83	0.92	**0.88**	0.86	0.95	**0.91**	0.94	0.75	**0.84**	--	--	--
Malignant T-cell-amplified sequence 1	Q9ULC4	MCTS1	1.05	0.69	**0.87**	1.14	0.92	**1.03**	0.98	0.91	**0.94**	--	--	--
14-3-3 protein gamma	P61981	YWHAG	0.74	0.98	**0.86**	0.88	0.89	**0.89**	0.74	0.59	**0.66**	--	--	--
Calcium-binding protein 39	A0A024R496	CAB39	0.68	1.03	**0.86**	0.85	0.83	**0.84**	1.08	1.11	**1.10**	--	--	--
Ribosome maturation protein SBDS	A0A0S2Z5I7	SBDS	0.64	1.06	**0.85**	0.71	1.08	**0.90**	0.61	0.66	**0.63**	--	--	--
Aflatoxin B1 aldehyde reductase member 2	H3BLU7	AKR7A2	0.74	0.94	**0.84**	1.25	0.73	**0.99**	1.12	0.79	**0.95**	--	--	--
Non-syndromic hearing impairment protein 5	A4FVA8	DFNA5	0.81	0.87	**0.84**	0.91	1.51	**1.21**	1.16	1.06	**1.11**	--	--	--
Transgelin-2	P37802	TAGLN2	0.65	1.02	**0.83**	0.84	1.08	**0.96**	0.61	0.82	**0.71**	--	--	--
Signal transducer and activator of transcription 1-alpha/beta	P42224	STAT1	0.64	1.02	**0.83**	0.84	0.92	**0.88**	0.73	0.73	**0.73**	--	--	--
Prefoldin subunit 3	Q6FH24	VBP1	0.85	0.79	**0.82**	1.02	0.77	**0.89**	0.75	0.82	**0.79**	--	--	--
Apoptosis regulator BAX	Q07812	BAX	0.71	0.92	**0.82**	1.18	0.84	**1.01**	1.12	1.00	**1.06**	--	--	--
Annexin	B4DT77	ANXA7	0.69	0.94	**0.81**	1.06	1.30	**1.18**	0.93	0.91	**0.92**	--	--	--
14-3-3 protein epsilon	V9HW98	YWHAE	0.73	0.89	**0.81**	0.94	0.83	**0.88**	0.92	0.76	**0.84**	--	--	--
Platelet-activating factor acetylhydrolase IB subunit alpha	P43034	PAFAH1B1	0.66	0.95	**0.81**	0.77	0.84	**0.81**	0.60	0.88	**0.74**	--	--	--
14-3-3 protein beta/alpha	V9HWD6	YWHAB	0.69	0.92	**0.81**	0.89	0.88	**0.88**	0.82	0.61	**0.71**	--	--	--
Cytosol aminopeptidase	V9HW38	LAP3	0.79	0.81	**0.80**	0.98	0.77	**0.87**	0.93	0.84	**0.89**	--	--	--
GMP reductase	Q86SZ5	GMPR2	0.69	0.88	**0.79**	1.11	1.05	**1.08**	1.75	1.05	**1.40**	--	--	--
Aspartyl aminopeptidase	A0A024R442	DNPEP	0.63	0.91	**0.77**	0.96	0.75	**0.86**	1.16	0.93	**1.04**	--	--	--
14-3-3 protein eta	A0A024R1K7	YWHAH	0.63	0.88	**0.76**	0.68	0.81	**0.75**	0.63	0.60	**0.61**	--	--	--
Ubiquitin carboxyl-terminal hydrolase	B2RD79	USP14	0.68	0.81	**0.74**	0.88	0.69	**0.79**	0.65	0.71	**0.68**	--	--	--
Drebrin-like protein	Q9UJU6	DBNL	0.60	0.89	**0.74**	0.80	0.81	**0.80**	0.72	1.18	**0.95**	--	--	--
28 kDa heat- and acid-stable phosphoprotein	Q13442	PDAP1	0.83	0.64	**0.74**	0.70	0.79	**0.74**	0.73	0.92	**0.82**	--	--	--
Leucine-rich repeat-containing protein 40	A0A140VJN3	LRRC40	0.62	0.84	**0.73**	--	--	--	0.91	1.05	**0.98**	1.13	0.68	**0.91**
Asparagine--tRNA ligase, cytoplasmic	O43776	NARS	0.69	0.75	**0.72**	0.82	0.70	**0.76**	0.65	0.62	**0.63**	--	--	--
Guanylate kinase	Q6IBG8	GUK1	0.83	0.61	**0.72**	1.01	0.78	**0.89**	0.81	1.00	**0.91**	--	--	--
Vacuolar protein sorting-associated protein VTA1 homolog	A0A087WY55	VTA1	0.66	0.78	**0.72**	1.06	0.83	**0.94**	1.22	1.03	**1.13**	--	--	--
Hsp90 co-chaperone Cdc37	A0A024R7B7	CDC37	0.60	0.81	**0.71**	--	--	--	0.89	1.09	**0.99**	1.11	1.02	**1.07**

S3D Table. *Cont.*

Protein name	Uniprot	Symbol	6 hpi			12 hpi			24 hpi			36 hpi		
			Ad2(L)/ Mock(H)	Mock(L)/ Ad2(H)	Average	Ad2(L)/ Mock(H)	Mock(L)/ Ad2(H)	Average	Ad2(L)/ Mock(H)	Mock(L)/ Ad2(H)	Average	Ad2(L)/ Mock(H)	Mock(L)/ Ad2(H)	Average
Heat shock protein beta-6	V9HWB6	HEL55	0.66	0.73	**0.69**	1.06	0.79	**0.93**	1.03	0.79	**0.91**	--	--	--
Sorting nexin-1	Q59GU6	SNX1	0.59	0.79	**0.69**	0.65	1.01	**0.83**	0.96	0.99	**0.97**	--	--	--
Hydroxyacylglutathione hydrolase, mitochondrial	H3BPK3	HAGH	0.62	0.71	**0.66**	0.94	0.96	**0.95**	0.86	0.65	**0.75**	--	--	--
Coatomer subunit gamma-1	Q9Y678	COPG1	0.66	0.66	**0.66**	0.89	0.65	**0.77**	0.66	0.66	**0.66**	--	--	--
NSFL1 cofactor p47	Q53FE8	NSFL1C	0.67	0.64	**0.66**	0.85	0.65	**0.75**	0.75	1.13	**0.94**	--	--	--
Partner of Y14 and mago	Q9BRP8	WIBG	0.63	0.66	**0.64**	--	--	--	0.96	1.22	**1.09**	1.11	1.26	**1.19**
Procollagen C-endopeptidase enhancer 1	Q15113	PCOLCE	-0.61	-1.11	**-0.86**	-0.84	-1.16	**-1.00**	--	--	--	-1.35	-1.01	**-1.18**
Connective tissue growth factor	B3KRV6	CTGF	-1.16	-0.94	**-1.05**	-1.55	-1.62	**-1.59**	-0.79	-2.21	**-1.50**	--	--	--
Integrin beta	L7RT22	ITGB5	-1.25	-1.19	**-1.22**	-1.83	-1.39	**-1.61**	-1.84	-2.09	**-1.96**	--	--	--
Thymosin beta-10	P63313	TMSB10	--	--	--	2.26	2.07	**2.16**	1.91	2.04	**1.97**	1.83	1.27	**1.55**
Creatine kinase B-type	V9HWH2	CKB	--	--	--	1.84	1.35	**1.59**	3.13	2.46	**2.80**	2.78	2.43	**2.61**
Caspase-3	P42574	CASP3	--	--	--	1.55	1.35	**1.45**	0.97	1.43	**1.20**	0.78	0.71	**0.74**
Thimet oligopeptidase	Q96CV8	THOP1	--	--	--	0.75	1.95	**1.35**	1.62	1.17	**1.39**	1.26	0.78	**1.02**
Fatty acid-binding protein, epidermal	E7DVW5	FABP5	--	--	--	1.92	0.77	**1.34**	3.10	2.01	**2.55**	3.34	2.80	**3.07**
Bisphosphoglycerate mutase	A0A024R782	BPGM	--	--	--	1.67	0.96	**1.32**	1.71	0.60	**1.15**	1.67	0.67	**1.17**
Dual specificity mitogen-activated protein kinase kinase 2	P36507	MAP2K2	--	--	--	1.45	1.14	**1.30**	1.40	1.46	**1.43**	1.42	1.02	**1.22**
Tubulin--tyrosine ligase-like protein 12	A0A024R4U3	TTLL12	--	--	--	1.17	1.25	**1.21**	1.24	1.28	**1.26**	1.69	1.11	**1.40**
60S ribosomal protein L38	A0A024R8P8	RPL38	--	--	--	0.97	1.38	**1.17**	1.92	1.84	**1.88**	2.21	2.13	**2.17**
Cysteine and histidine-rich domain-containing protein 1	A0A024R394	CHORDC1	--	--	--	1.33	1.01	**1.17**	2.05	1.94	**1.99**	2.36	1.90	**2.13**
Ubiquitin-fold modifier-conjugating enzyme 1	Q9Y3C8	UFC1	--	--	--	1.10	1.04	**1.07**	1.35	1.39	**1.37**	1.23	1.13	**1.18**
Serpin B6	A0A024QZX3	SERPINB6	--	--	--	1.19	0.95	**1.07**	1.18	1.17	**1.18**	0.98	0.75	**0.87**
Serine/threonine-protein phosphatase	Q9BPW0	PPP5C	--	--	--	0.99	1.15	**1.07**	1.52	1.52	**1.52**	0.72	1.28	**1.00**
Copine-3	A0A024R994	CPNE3	--	--	--	1.17	0.95	**1.06**	0.96	1.23	**1.09**	0.92	0.63	**0.77**
MOB kinase activator 1A	Q9H8S9	MOB1A	--	--	--	1.13	0.97	**1.05**	1.13	1.01	**1.07**	0.97	0.70	**0.84**
PDZ and LIM domain protein 2	B3KPU0	PDLIM2	--	--	--	0.97	1.08	**1.03**	1.25	1.03	**1.14**	1.55	0.88	**1.21**
Protein-L-isoaspartate O-methyltransferase	A0A0A0MRJ6	PCMT1	--	--	--	1.05	0.94	**0.99**	1.07	1.31	**1.19**	1.04	0.76	**0.90**
1,4-alpha-glucan-branching enzyme	Q59ET0	GBE1	--	--	--	0.94	1.05	**0.99**	0.94	1.04	**0.99**	0.84	0.70	**0.77**
Ubiquitin-like protein 5	B2R4N3	UBL5	--	--	--	0.76	1.20	**0.98**	1.63	2.17	**1.90**	1.55	1.52	**1.54**
L-aminoadipate-semialdehyde dehydrogenase-phosphopantetheinyl	Q9NRN7	AASDHPPT	--	--	--	0.79	1.15	**0.97**	1.66	2.02	**1.84**	1.63	1.47	**1.55**
Eukaryotic peptide chain release factor GTP-binding subunit ERF3A	H3BR35	GSPT1	--	--	--	1.04	0.84	**0.94**	0.96	1.06	**1.01**	1.15	0.87	**1.01**
Transcription factor p65	Q2TAM5	RELA	--	--	--	0.96	0.92	**0.94**	0.73	1.30	**1.02**	0.87	0.67	**0.77**
Triosephosphate isomerase	V9HWK1	TPI1	--	--	--	1.06	0.77	**0.91**	1.16	0.83	**1.00**	1.23	0.70	**0.96**
Importin-4	B3KQ33	IPO4	--	--	--	0.87	0.93	**0.90**	1.14	1.44	**1.29**	1.64	1.39	**1.51**
Farnesyl pyrophosphate synthase	P14324	FDPS	--	--	--	0.91	0.87	**0.89**	0.81	1.07	**0.94**	0.64	1.00	**0.82**
PEST proteolytic signal-containing nuclear protein	Q8WW12	PCNP	--	--	--	1.09	0.68	**0.89**	2.01	1.55	**1.78**	1.89	1.36	**1.62**
Protein unc-45 homolog A	Q9H3U1	UNC45A	--	--	--	0.80	0.97	**0.88**	0.73	1.09	**0.91**	1.09	0.79	**0.94**
Activator of 90 kDa heat shock protein ATPase homolog 1	O95433	AHSA1	--	--	--	1.04	0.64	**0.84**	1.61	1.62	**1.61**	1.83	1.83	**1.83**
Protein phosphatase methylesterase 1	A0A140VK39	PPME1	--	--	--	0.77	0.89	**0.83**	1.18	1.21	**1.19**	1.36	0.80	**1.08**
Plasminogen activator inhibitor 1 RNA-binding protein	Q8NC51	SERBP1	--	--	--	0.84	0.81	**0.83**	0.93	1.85	**1.39**	2.89	2.11	**2.50**
Sorting nexin-2	B3KN57	SNX2	--	--	--	0.87	0.77	**0.82**	1.19	1.19	**1.19**	1.59	0.80	**1.20**
IST1 homolog	A8K5S3	IST1	--	--	--	0.87	0.77	**0.82**	1.48	1.38	**1.43**	1.53	0.83	**1.18**
Growth factor receptor-bound protein 2	Q6ICN0	GRB2	--	--	--	0.74	0.90	**0.82**	0.85	1.25	**1.05**	0.94	0.86	**0.90**
Peptidyl-prolyl cis-trans isomerase FKBP4	Q02790	FKBP4	--	--	--	0.84	0.76	**0.80**	1.59	1.78	**1.69**	1.56	1.25	**1.40**
Elongation factor 1-alpha	Q6IPT9	EEF1A1	--	--	--	0.78	0.81	**0.80**	0.76	0.95	**0.86**	0.81	0.73	**0.77**
Leucine-rich repeat-containing protein 47	A0A024R4G1	LRRC47	--	--	--	0.98	0.61	**0.80**	0.87	0.65	**0.76**	1.05	0.62	**0.84**
S-adenosylmethionine synthase isoform type-2	A0A140VJP5	MAT2A	--	--	--	0.86	0.70	**0.78**	1.27	1.16	**1.22**	1.53	1.19	**1.36**
Transcription elongation factor B polypeptide 1	E5RHG8	TCEB1	--	--	--	0.73	0.78	**0.75**	0.75	0.92	**0.83**	0.97	0.63	**0.80**
Tyrosine-protein phosphatase non-receptor type 11	Q06124	PTPN11	--	--	--	0.73	0.75	**0.74**	0.93	1.21	**1.07**	1.19	0.75	**0.97**

S3D Table. *Cont.*

Protein name	Uniprot	Symbol	6 hpi			12 hpi			24 hpi			36 hpi		
			Ad2(L)/ Mock(H)	Mock(L)/ Ad2(H)	Average	Ad2(L)/ Mock(H)	Mock(L)/ Ad2(H)	Average	Ad2(L)/ Mock(H)	Mock(L)/ Ad2(H)	Average	Ad2(L)/ Mock(H)	Mock(L)/ Ad2(H)	Average
Translation initiation factor eIF-2B subunit delta	A0A087WTA5	EIF2B4	--	--	--	0.74	0.73	**0.73**	0.73	1.13	**0.93**	1.44	0.79	**1.12**
Heat shock 70 kDa protein 1B	A8K5I0	HSPA1B	--	--	--	0.86	0.60	**0.73**	3.65	3.59	**3.62**	3.36	3.31	**3.33**
Exportin-5	A8K5Y7	XPO5	--	--	--	0.83	0.63	**0.73**	1.23	1.25	**1.24**	1.60	1.22	**1.41**
ATP-binding cassette sub-family E member 1	P61221	ABCE1	--	--	--	0.74	0.71	**0.73**	0.88	1.21	**1.05**	1.17	1.06	**1.12**
Heat shock protein HSP 90-alpha	K9JA46	EL52	--	--	--	0.75	0.67	**0.71**	0.99	1.05	**1.02**	1.04	1.00	**1.02**
Coiled-coil domain-containing protein 124	A0A024R7M8	CCDC124	--	--	--	0.62	0.68	**0.65**	0.78	1.04	**0.91**	1.14	0.91	**1.03**
Alpha-taxilin	P40222	TXLNA	--	--	--	0.62	0.65	**0.64**	0.81	0.97	**0.89**	0.78	0.63	**0.70**
Ribose-phosphate pyrophosphokinase 1	Q53FW2	PRPS1	--	--	--	0.63	0.64	**0.63**	1.22	1.01	**1.11**	1.17	1.16	**1.16**
60S ribosomal protein L3	P39023	RPL3	--	--	--	0.63	0.61	**0.62**	0.82	0.97	**0.89**	0.84	1.04	**0.94**
Proliferation-associated protein 2G4	A0A024RB85	PA2G4	--	--	--	0.60	0.60	**0.60**	0.74	1.25	**1.00**	1.05	1.11	**1.08**
General transcription factor II-I	X5D2J9	GTF2I	--	--	--	-0.62	-0.78	**-0.70**	-1.06	-0.73	**-0.89**	-1.16	-2.00	**-1.58**
Collagen alpha-1(V) chain	B2ZZ86	COL5A1	--	--	--	-1.19	-0.78	**-0.98**	-1.47	-0.97	**-1.22**	-1.77	-1.55	**-1.66**
Metalloproteinase inhibitor 3	P35625	TIMP3	--	--	--	-1.36	-0.78	**-1.07**	-1.90	-2.04	**-1.97**	-2.25	-1.97	**-2.11**
Collagen alpha-1(XII) chain	D6RGG3	COL12A1	--	--	--	-1.45	-0.92	**-1.19**	-1.66	-1.25	**-1.45**	-2.05	-1.55	**-1.80**
	D3DTX7	COL1A1	--	--	--	-1.20	-1.28	**-1.24**	-1.90	-1.66	**-1.78**	-2.84	-2.64	**-2.74**
Thymosin beta-4	A2VCK8	TMSB4X	1.72	2.16	**1.94**	2.13	1.85	**1.99**	1.40	1.79	**1.60**	1.34	1.01	**1.17**
Glutamate--cysteine ligase catalytic subunit	Q14TF0	GCLC	1.90	1.73	**1.81**	1.94	1.40	**1.67**	1.62	1.29	**1.45**	1.69	0.60	**1.14**
SH3 domain-binding glutamic acid-rich-like protein	V9HW48	SH3BGRL	1.47	1.94	**1.71**	1.94	1.83	**1.88**	1.81	1.80	**1.80**	1.68	1.13	**1.40**
Thioredoxin domain-containing protein 17	A0A140VJY7	TXNDC17	1.39	1.96	**1.67**	1.83	1.75	**1.79**	1.90	1.85	**1.87**	2.03	1.39	**1.71**
Glutathione S-transferase Mu 3	Q6FGJ9	GSTM3	1.57	1.68	**1.62**	1.84	1.40	**1.62**	1.63	1.40	**1.52**	1.74	1.02	**1.38**
Peptidyl-prolyl cis-trans isomerase FKBP1A	P62942	FKBP1A	1.40	1.79	**1.59**	1.89	1.81	**1.85**	1.96	1.64	**1.80**	1.99	1.18	**1.59**
SH3 domain-binding glutamic acid-rich-like protein 3	Q5T123	SH3BGRL3	1.38	1.76	**1.57**	1.59	1.63	**1.61**	1.55	1.60	**1.58**	1.51	0.98	**1.25**
Cystatin-B	Q76LA1	CSTB	1.15	1.91	**1.53**	1.63	1.81	**1.72**	1.79	1.79	**1.79**	1.77	1.45	**1.61**
Glutaredoxin-1	A0A024RAM2	GLRX	1.06	1.99	**1.52**	1.58	1.87	**1.73**	1.63	1.58	**1.61**	1.66	1.21	**1.43**
Aspartate aminotransferase, cytoplasmic	A0A140VK69	GOT1	1.43	1.60	**1.52**	1.46	1.40	**1.43**	1.41	1.11	**1.26**	1.24	0.90	**1.07**
Rho GDP-dissociation inhibitor 1	J3KTF8	ARHGDIA	1.34	1.65	**1.50**	1.53	1.52	**1.53**	1.42	1.30	**1.36**	1.09	0.85	**0.97**
Phosphatidylethanolamine-binding protein 1	D9IAI1	PEBP1	1.31	1.68	**1.49**	1.93	1.63	**1.78**	1.78	1.98	**1.88**	2.01	1.30	**1.65**
Thioredoxin	H9ZYJ2	TXN	1.24	1.73	**1.49**	1.79	1.79	**1.79**	1.94	1.91	**1.92**	2.10	1.54	**1.82**
Histidine triad nucleotide-binding protein 1	P49773	HINT1	1.33	1.62	**1.47**	1.59	1.55	**1.57**	1.79	1.58	**1.68**	2.11	1.25	**1.68**
Myotrophin	Q69YG1	MTPN	1.29	1.66	**1.47**	1.74	1.58	**1.66**	1.92	1.74	**1.83**	1.51	1.14	**1.33**
Macrophage migration inhibitory factor	I4AY87	MIF	1.27	1.64	**1.45**	1.09	1.49	**1.29**	1.81	1.72	**1.76**	2.18	1.21	**1.70**
Latexin	Q9BS40	LXN	1.11	1.78	**1.45**	2.14	1.64	**1.89**	1.94	2.03	**1.98**	1.53	1.10	**1.32**
Profilin-1	P07737	PFN1	1.15	1.73	**1.44**	1.43	1.54	**1.49**	1.42	1.40	**1.41**	1.71	0.93	**1.32**
Rab GDP dissociation inhibitor alpha	A0A0S2Z3X8	GDI1	1.30	1.57	**1.44**	1.57	1.39	**1.48**	1.46	1.37	**1.42**	1.51	1.11	**1.31**
Tubulin-specific chaperone A	E5RIW3	TBCA	1.42	1.45	**1.43**	1.82	1.37	**1.59**	2.08	2.00	**2.04**	2.13	1.51	**1.82**
BolA-like protein 2	Q9H3K6	BOLA2	1.13	1.70	**1.41**	1.69	1.33	**1.51**	2.04	1.84	**1.94**	2.04	1.41	**1.73**
Rab GDP dissociation inhibitor beta	Q6IAT1	GDI2	1.28	1.51	**1.40**	1.57	1.38	**1.47**	1.37	1.35	**1.36**	1.39	0.80	**1.09**
Obg-like ATPase 1	J3KQ32	OLA1	1.18	1.61	**1.40**	1.66	1.44	**1.55**	1.48	1.73	**1.61**	1.80	1.26	**1.53**
D-dopachrome decarboxylase	Q53Y51	DDT	1.12	1.65	**1.38**	1.55	1.47	**1.51**	1.88	1.72	**1.80**	2.15	1.60	**1.87**
NEDD8	E9PL57	NEDD8	1.26	1.49	**1.38**	1.96	1.14	**1.55**	1.61	1.66	**1.64**	1.71	1.23	**1.47**
Tubulin alpha-1C chain	Q53GA7	TUBA1C	1.14	1.59	**1.37**	1.27	1.42	**1.34**	1.06	1.21	**1.14**	1.31	0.86	**1.08**
Branched-chain-amino-acid aminotransferase	A0A024RAV0	BCAT1	1.27	1.46	**1.36**	1.42	1.40	**1.41**	1.21	1.05	**1.13**	0.87	0.69	**0.78**
Phosphoserine aminotransferase	A0A024R222	PSAT1	1.27	1.45	**1.36**	1.53	1.34	**1.44**	1.61	1.41	**1.51**	1.74	1.13	**1.43**
Secernin-1	A0A090N7T9	SCRN1	1.08	1.61	**1.34**	1.52	1.43	**1.47**	1.12	1.18	**1.15**	1.02	0.66	**0.84**
Coactosin-like protein	Q14019	COTL1	1.18	1.49	**1.33**	1.38	1.38	**1.38**	1.47	1.38	**1.42**	1.54	0.94	**1.24**
Ran-specific GTPase-activating protein	A0A140VK94	RANBP1	1.30	1.33	**1.32**	1.66	1.26	**1.46**	2.16	1.65	**1.91**	2.04	1.75	**1.90**
Fascin	Q16658	FSCN1	1.17	1.46	**1.32**	1.54	1.36	**1.45**	1.30	1.39	**1.34**	1.47	0.90	**1.19**
Gamma-enolase	Q6FHV6	ENO2	1.11	1.52	**1.31**	1.51	1.31	**1.41**	1.27	1.06	**1.16**	1.20	0.90	**1.05**

***S3D Table**. Cont.*

Protein name	Uniprot	Symbol	6 hpi			12 hpi			24 hpi			36 hpi		
			Ad2(L)/ Mock(H)	Mock(L)/ Ad2(H)	Average	Ad2(L)/ Mock(H)	Mock(L)/ Ad2(H)	Average	Ad2(L)/ Mock(H)	Mock(L)/ Ad2(H)	Average	Ad2(L)/ Mock(H)	Mock(L)/ Ad2(H)	Average
Cytosolic non-specific dipeptidase	B4DV28	CNDP2	1.25	1.37	**1.31**	1.69	1.25	**1.47**	1.25	1.41	**1.33**	1.90	0.73	**1.31**
	B4DY32		1.30	1.32	**1.31**	1.27	1.34	**1.30**	1.18	1.21	**1.20**	1.54	0.86	**1.20**
Ubiquitin-conjugating enzyme E2 L3	A8K4W8	UBE2L3	1.20	1.41	**1.31**	1.52	1.28	**1.40**	1.44	1.19	**1.32**	1.54	0.64	**1.09**
Annexin A6	A0A0S2Z2Z6	ANXA6	1.12	1.49	**1.31**	1.37	1.23	**1.30**	1.10	1.05	**1.07**	1.00	0.76	**0.88**
Aldose reductase	A0A024R7A8	AKR1B1	1.28	1.32	**1.30**	1.91	1.29	**1.60**	1.95	1.58	**1.76**	2.09	1.29	**1.69**
Xaa-Pro aminopeptidase 1	Q9NQW7	XPNPEP1	1.24	1.36	**1.30**	1.31	1.17	**1.24**	1.25	1.13	**1.19**	1.32	0.70	**1.01**
Ubiquitin carboxyl-terminal hydrolase	V9HW74	UCHL1	1.17	1.42	**1.29**	1.48	1.38	**1.43**	1.47	1.22	**1.34**	1.60	0.95	**1.27**
Alcohol dehydrogenase [NADP(+)]	V9HWI0	AKR1A1	1.21	1.37	**1.29**	1.77	1.27	**1.52**	1.82	1.11	**1.47**	1.79	0.91	**1.35**
Rap1 GTPase-GDP dissociation stimulator 1	P52306	RAP1GDS1	1.21	1.36	**1.29**	1.26	1.25	**1.26**	1.01	1.29	**1.15**	0.96	0.63	**0.79**
Tubulin beta-3 chain	Q9BV28	TUBB3	0.93	1.63	**1.28**	1.56	1.56	**1.56**	1.05	1.46	**1.26**	1.30	0.61	**0.96**
Glucose-6-phosphate 1-dehydrogenase	P11413	G6PD	1.09	1.46	**1.28**	1.44	1.27	**1.35**	1.28	1.18	**1.23**	1.40	0.89	**1.15**
BH3-interacting domain death agonist	B1PL87	BID	0.91	1.65	**1.28**	1.40	1.49	**1.44**	1.54	1.78	**1.66**	1.48	1.23	**1.36**
Tubulin beta-6 chain	Q9BUF5	TUBB6	0.94	1.61	**1.27**	1.35	1.53	**1.44**	0.77	0.98	**0.88**	0.74	0.59	**0.66**
Protein S100	R4GN98	S100A6	1.13	1.41	**1.27**	1.50	1.36	**1.43**	1.51	1.43	**1.47**	1.31	1.01	**1.16**
Tubulin alpha-4A chain	P68366	TUBA4A	1.07	1.48	**1.27**	1.16	1.58	**1.37**	0.98	1.32	**1.15**	1.12	0.88	**1.00**
Isocitrate dehydrogenase [NADP]	V9HWJ2	IDH1	1.17	1.36	**1.27**	1.52	1.21	**1.37**	1.50	1.24	**1.37**	1.56	0.77	**1.16**
Fructose-bisphosphate aldolase	V9HWN7	ALDOA	1.15	1.38	**1.26**	1.44	1.24	**1.34**	1.33	1.22	**1.28**	1.49	0.98	**1.23**
Astrocytic phosphoprotein PEA-15	B1AKZ5	PEA15	0.90	1.63	**1.26**	1.11	1.57	**1.34**	1.23	1.42	**1.32**	0.87	0.74	**0.81**
Protein S100	V9HWH9	S100A11	0.97	1.55	**1.26**	1.37	1.45	**1.41**	1.43	1.38	**1.41**	1.42	1.09	**1.25**
Serpin B8	A0A024R2B1	SERPINB8	0.83	1.69	**1.26**	0.94	1.04	**0.99**	0.71	0.74	**0.72**	1.17	0.59	**0.88**
Tryptophan--tRNA ligase, cytoplasmic	A0A024R6K8	WARS	1.10	1.42	**1.26**	1.29	1.31	**1.30**	0.84	0.98	**0.91**	1.16	0.71	**0.93**
Adenylate kinase isoenzyme 1	Q6FGX9	AK1	0.99	1.52	**1.26**	1.58	1.48	**1.53**	1.51	1.69	**1.60**	1.45	0.72	**1.09**
Fumarylacetoacetase	P16930	FAH	1.29	1.21	**1.25**	1.41	0.97	**1.19**	1.31	1.00	**1.15**	1.26	0.73	**0.99**
Translationally-controlled tumor protein	A0A0P1J1R0	TPT1	1.09	1.41	**1.25**	1.36	1.36	**1.36**	1.69	1.85	**1.77**	2.04	1.52	**1.78**
Nuclear transport factor 2	A0A024R6Y2	NUTF2	1.07	1.43	**1.25**	1.54	1.37	**1.46**	1.31	1.31	**1.31**	1.53	0.89	**1.21**
Cystathionine beta-synthase	B7Z2D6	CBS	1.19	1.30	**1.25**	1.19	1.18	**1.19**	1.30	1.23	**1.26**	1.55	1.07	**1.31**
Phosphoglycerate mutase	Q6P6D7	PGAM1	1.10	1.40	**1.25**	1.47	1.29	**1.38**	1.48	1.09	**1.28**	1.48	0.80	**1.14**
Malate dehydrogenase	V9HWF2	MDH1	1.10	1.38	**1.24**	1.46	1.20	**1.33**	1.43	1.12	**1.27**	1.35	1.03	**1.19**
Alpha-enolase	A0A024R4F1	ENO1	1.10	1.38	**1.24**	1.49	1.23	**1.36**	1.21	1.30	**1.26**	1.12	1.03	**1.08**
Alanine--tRNA ligase, cytoplasmic	P49588	AARS	1.17	1.31	**1.24**	1.34	1.11	**1.23**	1.21	1.12	**1.17**	1.25	0.66	**0.96**
6-phosphogluconate dehydrogenase, decarboxylating	P52209	PGD	1.17	1.31	**1.24**	1.61	1.28	**1.45**	1.33	0.97	**1.15**	1.20	0.72	**0.96**
Glutathione S-transferase P	V9HWE9	GSTP1	1.14	1.33	**1.23**	1.53	1.21	**1.37**	1.23	1.33	**1.28**	1.50	0.75	**1.12**
Glucosamine-6-phosphate isomerase	A8K3S1	GNPDA1	1.07	1.39	**1.23**	1.66	1.26	**1.46**	0.74	1.12	**0.93**	1.12	0.63	**0.87**
Lactoylglutathione lyase	X5DNM4	GLO1	1.06	1.41	**1.23**	1.58	1.37	**1.48**	1.60	1.60	**1.60**	1.62	1.29	**1.45**
Glucose-6-phosphate isomerase	B4DVJ0		1.06	1.41	**1.23**	1.43	1.26	**1.35**	1.23	1.37	**1.30**	1.23	0.94	**1.08**
Purine nucleoside phosphorylase	V9HWH6	PNP	1.10	1.36	**1.23**	1.51	1.34	**1.42**	1.99	1.84	**1.92**	2.10	1.81	**1.96**
Spermidine synthase	P19623	SRM	1.09	1.37	**1.23**	1.38	1.30	**1.34**	1.35	1.23	**1.29**	1.36	0.92	**1.14**
Nicotinamide N-methyltransferase	Q6FH49	NNMT	0.93	1.53	**1.23**	1.33	1.41	**1.37**	1.23	0.93	**1.08**	0.89	0.69	**0.79**
S-(hydroxymethyl)glutathione dehydrogenase	Q6IRT1	ADH5	1.13	1.32	**1.23**	1.51	1.13	**1.32**	1.45	1.17	**1.31**	1.30	0.73	**1.01**
Peptidyl-prolyl cis-trans isomerase	V9HWF5	PPIA	1.09	1.35	**1.22**	1.46	1.34	**1.40**	1.39	1.55	**1.47**	1.55	1.15	**1.35**
6-phosphogluconolactonase	A0A0K0K1K7	PGLS	1.04	1.40	**1.22**	1.53	1.22	**1.38**	1.53	1.14	**1.33**	1.41	0.67	**1.04**
Fructose-bisphosphate aldolase	A0A024QZ64	ALDOC	1.07	1.37	**1.22**	1.42	1.12	**1.27**	1.38	1.26	**1.32**	1.56	0.77	**1.17**
Heme-binding protein 2	Q05DB4	HEBP2	0.96	1.48	**1.22**	1.46	1.07	**1.27**	1.61	1.02	**1.32**	1.24	0.72	**0.98**
Tetratricopeptide repeat protein 38	B4DUE0	TTC38	1.09	1.34	**1.22**	0.87	1.00	**0.93**	1.03	1.77	**1.40**	1.22	1.18	**1.20**
Phosphoglycerate kinase	V9HWF4	PGK1	1.04	1.39	**1.21**	1.54	1.21	**1.37**	1.52	1.27	**1.40**	1.63	1.13	**1.38**
Peroxiredoxin-6	V9HWC7	PRDX6	1.06	1.37	**1.21**	1.18	1.23	**1.20**	1.08	1.07	**1.08**	1.09	0.77	**0.93**
Adenylosuccinate synthetase isozyme 2	A0A024R5Q7	ADSS	1.02	1.40	**1.21**	1.66	1.33	**1.50**	1.45	1.42	**1.43**	1.44	0.98	**1.21**
Ubiquitin-conjugating enzyme E2 variant 1	Q13404	UBE2V1	1.14	1.28	**1.21**	1.44	1.16	**1.30**	1.55	1.36	**1.45**	1.66	0.85	**1.25**

***S3D Table**. Cont.*

Protein name	Uniprot	Symbol	6 hpi			12 hpi			24 hpi			36 hpi		
			Ad2(L)/ Mock(H)	Mock(L)/ Ad2(H)	Average	Ad2(L)/ Mock(H)	Mock(L)/ Ad2(H)	Average	Ad2(L)/ Mock(H)	Mock(L)/ Ad2(H)	Average	Ad2(L)/ Mock(H)	Mock(L)/ Ad2(H)	Average
Phosphoglucomutase-1	P36871	PGM1	1.19	1.23	**1.21**	1.40	1.11	**1.25**	1.22	1.33	**1.28**	1.51	1.16	**1.33**
Glyoxalase domain-containing protein 4	B4DX01	GLOD4	1.06	1.36	**1.21**	1.78	1.28	**1.53**	1.76	1.32	**1.54**	1.48	1.17	**1.33**
Nucleoside diphosphate kinase A	P15531	NME1	1.08	1.33	**1.21**	1.27	1.26	**1.26**	1.49	1.47	**1.48**	1.89	1.39	**1.64**
Glutathione S-transferase omega-1	V9HWG9	GSTO1	1.06	1.36	**1.21**	1.43	1.29	**1.36**	1.39	0.96	**1.17**	1.12	0.72	**0.92**
Profilin	C9J0J7	PFN2	1.10	1.31	**1.20**	1.44	1.30	**1.37**	1.28	1.28	**1.28**	1.24	0.82	**1.03**
UMP-CMP kinase	P30085	CMPK1	0.92	1.48	**1.20**	1.38	1.13	**1.26**	1.50	1.44	**1.47**	1.50	1.15	**1.32**
Sorbitol dehydrogenase	V9HW89	SORD	1.08	1.31	**1.20**	1.52	1.06	**1.29**	1.31	1.30	**1.30**	1.40	0.95	**1.18**
Prolyl endopeptidase	Q9UM02	PREP	1.21	1.18	**1.20**	1.59	0.99	**1.29**	1.28	1.13	**1.20**	1.38	0.88	**1.13**
Aminopeptidase B	Q9H4A4	RNPEP	1.26	1.12	**1.19**	1.55	0.80	**1.18**	1.47	1.07	**1.27**	1.48	0.59	**1.03**
Peroxiredoxin-2	V9HW12	PRDX2	1.12	1.24	**1.18**	1.49	1.21	**1.35**	1.33	1.41	**1.37**	1.40	0.67	**1.04**
26S proteasome non-ATPase regulatory subunit 9	J3KN29	PSMD9	1.11	1.24	**1.18**	1.48	1.05	**1.26**	1.22	1.14	**1.18**	0.97	0.61	**0.79**
Xaa-Pro dipeptidase	J3K000	PEPD	1.04	1.31	**1.18**	1.48	1.19	**1.34**	1.19	1.33	**1.26**	1.23	0.97	**1.10**
CTP synthase 1	P17812	CTPS1	1.04	1.30	**1.17**	1.14	0.96	**1.05**	1.46	1.28	**1.37**	1.63	1.34	**1.49**
Protein phosphatase 1F	A0M8Q2	PPM1F	1.08	1.26	**1.17**	1.35	1.37	**1.36**	1.14	1.52	**1.33**	1.33	1.13	**1.23**
Nicotinamide phosphoribosyltransferase	A0A024R718	PBEF1	1.29	1.04	**1.17**	2.26	0.83	**1.54**	2.14	1.52	**1.83**	1.98	0.90	**1.44**
Pyridoxal kinase	F2Z2Y4	PDXK	1.18	1.14	**1.16**	1.57	0.98	**1.28**	1.47	1.11	**1.29**	1.63	1.18	**1.41**
Nucleoside diphosphate kinase	Q6FHN3	NME2	0.97	1.35	**1.16**	1.21	1.29	**1.25**	1.47	1.45	**1.46**	1.47	1.26	**1.36**
Elongation factor 2	P13639	EEF2	0.99	1.31	**1.15**	1.24	1.17	**1.20**	1.51	1.46	**1.48**	1.90	1.42	**1.66**
Fructose-2,6-bisphosphatase TIGAR	Q9NQ88	TIGAR	0.94	1.36	**1.15**	1.44	1.33	**1.38**	1.52	1.40	**1.46**	1.73	1.20	**1.46**
L-lactate dehydrogenase	V9HWB9	LDHA	1.02	1.28	**1.15**	1.33	1.12	**1.23**	1.01	0.75	**0.88**	1.11	0.65	**0.88**
S-formylglutathione hydrolase	A0A140VJJ2	ESD	1.14	1.15	**1.15**	1.32	1.29	**1.30**	1.32	1.06	**1.19**	1.20	0.60	**0.90**
Phosphomannomutase 2	A0A0S2Z4J6	PMM2	1.09	1.21	**1.15**	1.56	1.19	**1.37**	1.69	0.79	**1.24**	1.60	1.12	**1.36**
Glutamine--fructose-6-phosphate aminotransferase [isomerizing] 1	Q06210	GFPT1	1.00	1.27	**1.14**	1.18	1.27	**1.23**	1.20	1.08	**1.14**	1.39	0.80	**1.10**
Tubulin-folding cofactor B	Q99426	TBCB	0.90	1.37	**1.14**	1.21	1.45	**1.33**	1.22	1.07	**1.15**	1.44	1.18	**1.31**
Threonine--tRNA ligase, cytoplasmic	Q53GX7	TARS	1.01	1.26	**1.13**	1.32	1.04	**1.18**	1.45	1.21	**1.33**	1.64	1.02	**1.33**
Ubiquitin-fold modifier 1	H0Y614	UFM1	0.94	1.32	**1.13**	1.37	1.30	**1.34**	1.49	1.35	**1.42**	1.06	1.12	**1.09**
Phosphatidylinositol transfer protein beta isoform	P48739	PITPNB	0.87	1.37	**1.12**	1.03	0.97	**1.00**	1.46	1.10	**1.28**	1.30	1.02	**1.16**
Serine/threonine-protein phosphatase 2A activator	A6PVN5	PPP2R4	0.93	1.30	**1.12**	1.49	1.11	**1.30**	1.43	1.39	**1.41**	1.23	0.87	**1.05**
Glia maturation factor beta	P60983	GMFB	0.99	1.23	**1.11**	1.24	1.33	**1.28**	1.53	1.61	**1.57**	1.62	1.24	**1.43**
Phosphoacetylglucosamine mutase	B3KN28	PGM3	0.94	1.28	**1.11**	1.49	1.12	**1.30**	1.39	1.05	**1.22**	1.26	0.83	**1.04**
L-lactate dehydrogenase	Q5U077	LDHB	1.10	1.11	**1.11**	1.51	1.05	**1.28**	1.53	1.03	**1.28**	1.70	1.18	**1.44**
Platelet-activating factor acetylhydrolase IB subunit beta	V9HW44	PAFAH1B2	1.13	1.08	**1.11**	1.52	0.87	**1.20**	1.36	1.06	**1.21**	0.94	0.63	**0.79**
Bifunctional purine biosynthesis protein PURH	V9HWH7	ATIC	1.02	1.19	**1.10**	1.28	1.00	**1.14**	1.29	1.29	**1.29**	1.62	1.15	**1.39**
Leukocyte elastase inhibitor	V9HWH1	SERPINB1	0.98	1.22	**1.10**	1.35	1.04	**1.19**	1.36	1.16	**1.26**	1.18	0.78	**0.98**
Adenine phosphoribosyltransferase	P07741	APRT	0.77	1.43	**1.10**	1.43	1.38	**1.41**	1.31	1.46	**1.38**	1.31	0.88	**1.10**
Trifunctional purine biosynthetic protein adenosine-3	Q3B7A7	GART	0.89	1.30	**1.09**	1.36	1.23	**1.29**	1.09	1.61	**1.35**	0.89	1.43	**1.16**
Annexin	V9HWE0	ANXA5	0.95	1.23	**1.09**	1.35	1.19	**1.27**	1.25	1.02	**1.14**	1.01	0.69	**0.85**
Glutathione synthetase	V9HWJ1	GSS	0.98	1.19	**1.09**	1.25	1.26	**1.26**	1.09	1.13	**1.11**	1.12	0.68	**0.90**
Platelet-activating factor acetylhydrolase IB subunit gamma	A0A024R0L6	PAFAH1B3	0.80	1.36	**1.08**	1.38	1.24	**1.31**	1.49	1.27	**1.38**	1.07	0.65	**0.86**
Dynamin-1-like protein	O00429	DNM1L	1.04	1.12	**1.08**	1.17	1.16	**1.16**	0.79	1.14	**0.96**	1.11	0.99	**1.05**
Dipeptidyl peptidase 3	Q53HL4	DPP3	1.02	1.13	**1.08**	1.39	0.94	**1.16**	1.01	1.04	**1.03**	1.19	0.77	**0.98**
Copine-1	F2Z2V0	CPNE1	0.98	1.17	**1.07**	1.36	0.98	**1.17**	1.34	1.08	**1.21**	1.34	0.73	**1.03**
Adenylosuccinate lyase	A0A1B0GWJ0	ADSL	0.96	1.18	**1.07**	1.13	1.03	**1.08**	1.02	0.79	**0.91**	1.18	0.59	**0.89**
Protein deglycase DJ-1	V9HWC2	PARK7	1.05	1.09	**1.07**	1.45	1.10	**1.27**	1.33	1.29	**1.31**	1.35	0.78	**1.07**
N-acetyl-D-glucosamine kinase	Q9UJ70	NAGK	0.96	1.18	**1.07**	1.25	1.01	**1.13**	0.96	0.95	**0.95**	0.89	0.86	**0.87**
ATP-dependent 6-phosphofructokinase, platelet type	Q01813	PFKP	0.85	1.29	**1.07**	1.07	1.06	**1.06**	1.01	0.99	**1.00**	1.33	0.96	**1.15**
Putative peptidyl-tRNA hydrolase PTRHD1	Q6GMV3	PTRHD1	0.90	1.24	**1.07**	1.17	1.12	**1.14**	1.42	1.57	**1.50**	1.42	0.94	**1.18**
	A0A090N8G0	GARS	0.93	1.20	**1.06**	1.05	1.02	**1.04**	0.86	0.86	**0.86**	1.04	0.61	**0.82**

S3D Table. *Cont.*

Protein name	Uniprot	Symbol	6 hpi			12 hpi			24 hpi			36 hpi		
			Ad2(L)/ Mock(H)	Mock(L)/ Ad2(H)	Average	Ad2(L)/ Mock(H)	Mock(L)/ Ad2(H)	Average	Ad2(L)/ Mock(H)	Mock(L)/ Ad2(H)	Average	Ad2(L)/ Mock(H)	Mock(L)/ Ad2(H)	Average
Serine--tRNA ligase, cytoplasmic	Q53HA4	SARS	0.91	1.21	**1.06**	0.99	1.12	**1.05**	0.95	1.08	**1.02**	1.02	0.87	**0.94**
Prostaglandin reductase 1	A0A024R172	LTB4DH	0.97	1.15	**1.06**	1.56	1.28	**1.42**	1.23	1.25	**1.24**	1.06	1.01	**1.04**
Four and a half LIM domains protein 1	B7Z6U8	FHL1	0.79	1.33	**1.06**	1.33	1.18	**1.25**	1.28	1.01	**1.14**	1.12	0.84	**0.98**
Ubiquitin-conjugating enzyme E2 N	V9HW41	UBE2N	0.89	1.22	**1.05**	1.23	1.10	**1.17**	1.38	1.26	**1.32**	1.51	0.95	**1.23**
Ubiquitin thioesterase OTUB1	F5GYN4	OTUB1	0.87	1.23	**1.05**	1.32	1.13	**1.22**	1.32	1.14	**1.23**	1.24	0.86	**1.05**
Carbonyl reductase [NADPH] 1	P16152	CBR1	0.91	1.18	**1.05**	1.42	1.31	**1.36**	1.48	1.04	**1.26**	1.33	0.65	**0.99**
Inositol monophosphatase 1	A0A024R830	IMPA1	0.63	1.46	**1.04**	1.42	1.22	**1.32**	1.35	1.51	**1.43**	1.22	0.79	**1.00**
ADP-sugar pyrophosphatase	A6NFX8	NUDT5	0.93	1.15	**1.04**	1.35	1.06	**1.20**	1.35	0.96	**1.16**	1.37	0.98	**1.18**
Annexin	V9HW59	ANXA4	0.92	1.16	**1.04**	1.38	1.27	**1.32**	1.32	1.17	**1.25**	1.15	0.86	**1.01**
Biliverdin reductase A	A0A140VJF4	BLVRA	0.81	1.27	**1.04**	1.15	1.19	**1.17**	1.34	1.15	**1.24**	1.33	0.76	**1.05**
Eukaryotic translation initiation factor 5A	I3L397	EIF5A	0.92	1.15	**1.03**	1.26	1.14	**1.20**	1.42	1.39	**1.40**	1.59	1.11	**1.35**
Phosphoribosylformylglycinamidine synthase	A8K9T9	PFAS	1.02	1.04	**1.03**	1.05	0.66	**0.86**	1.31	1.13	**1.22**	1.55	0.66	**1.11**
Low molecular weight phosphotyrosine protein phosphatase	Q59EH3	ACP1	0.93	1.13	**1.03**	1.35	1.10	**1.22**	1.48	1.14	**1.31**	2.10	1.46	**1.78**
Eukaryotic translation initiation factor 4H	Q15056	EIF4H	0.97	1.08	**1.03**	1.26	1.58	**1.42**	1.99	1.86	**1.93**	3.37	2.37	**2.87**
Multifunctional protein ADE2	A0A024RD93	PAICS	0.92	1.13	**1.02**	1.18	1.13	**1.16**	1.28	1.37	**1.32**	1.53	1.13	**1.33**
ATP-dependent 6-phosphofructokinase	A0A024R0Y5	PFKM	0.87	1.17	**1.02**	1.27	0.83	**1.05**	1.14	1.15	**1.14**	1.18	0.94	**1.06**
Ribonuclease inhibitor	A0A024RC87	RNH1	0.80	1.24	**1.02**	1.15	1.10	**1.13**	1.25	1.33	**1.29**	1.18	0.85	**1.02**
Adapter molecule crk	L7RT18	CRK	0.95	1.08	**1.01**	1.12	1.11	**1.11**	1.19	1.16	**1.17**	1.12	0.79	**0.96**
Serine/threonine-protein phosphatase 2A 55 kDa regulatory subunit B	A0A140VJT0	PPP2R2A	0.75	1.25	**1.00**	0.87	1.26	**1.06**	1.47	1.42	**1.45**	1.61	1.11	**1.36**
GDP-L-fucose synthase	A0A140VKC8	TSTA3	0.85	1.16	**1.00**	1.38	1.09	**1.23**	1.39	1.15	**1.27**	1.24	0.76	**1.00**
Guanine nucleotide-binding protein-like 1	B4DYK6	GNL1	0.78	1.21	**0.99**	1.13	0.72	**0.93**	1.21	1.12	**1.17**	0.93	0.91	**0.92**
N-alpha-acetyltransferase 10	P41227	NAA10	0.93	1.04	**0.98**	1.02	0.94	**0.98**	1.07	0.94	**1.01**	1.22	0.69	**0.96**
Destrin	V9HWA6	HEL32	0.75	1.19	**0.97**	0.85	1.19	**1.02**	0.89	1.03	**0.96**	0.97	0.66	**0.82**
Isochorismatase domain-containing protein 1	Q96CN7	ISOC1	0.98	0.95	**0.96**	1.23	0.87	**1.05**	1.41	1.00	**1.20**	1.42	0.65	**1.04**
Protein S100-A13	Q99584	S100A13	0.85	1.07	**0.96**	1.02	1.02	**1.02**	1.13	1.06	**1.09**	1.00	0.76	**0.88**
Protein CDV3 homolog	Q9UKY7	CDV3	0.81	1.11	**0.96**	1.07	0.86	**0.96**	1.11	1.12	**1.12**	1.01	1.11	**1.06**
Hematological and neurological expressed 1-like protein	Q9H910	HN1L	0.78	1.14	**0.96**	1.38	1.11	**1.25**	1.68	1.26	**1.47**	1.66	0.98	**1.32**
Nuclear migration protein nudC	Q9Y266	NUDC	0.91	1.00	**0.96**	1.02	0.91	**0.96**	1.66	1.43	**1.54**	1.55	1.51	**1.53**
Leukotriene A-4 hydrolase	A0A140VK27	LTA4H	1.18	0.73	**0.95**	1.12	0.63	**0.88**	1.17	1.06	**1.11**	1.66	0.71	**1.18**
Ester hydrolase C11orf54	A0A087WT99	C11orf54	0.91	0.99	**0.95**	1.74	0.92	**1.33**	1.33	1.44	**1.38**	1.25	0.64	**0.94**
Acetyl-CoA acetyltransferase, cytosolic	A8K4W5	ACAT2	0.71	1.19	**0.95**	1.28	1.11	**1.19**	1.32	1.01	**1.17**	1.25	0.92	**1.08**
Testin	B2RDR4	TES	0.98	0.91	**0.94**	1.32	0.99	**1.15**	0.93	1.03	**0.98**	1.33	0.69	**1.01**
ATP-dependent 6-phosphofructokinase, liver type	P17858	PFKL	0.68	1.19	**0.93**	1.17	0.82	**0.99**	0.83	0.77	**0.80**	1.14	0.68	**0.91**
Dual specificity mitogen-activated protein kinase kinase 1	B4DFY5	MAP2K1	0.92	0.94	**0.93**	1.57	1.02	**1.29**	1.51	1.23	**1.37**	1.44	1.06	**1.25**
Glyceraldehyde-3-phosphate dehydrogenase	V9HVZ4	GAPDH	0.91	0.95	**0.93**	1.28	0.89	**1.08**	0.94	0.93	**0.93**	0.95	0.59	**0.77**
Prefoldin subunit 1	E5RGS4	PFDN1	0.79	1.04	**0.92**	0.87	0.92	**0.90**	1.02	0.95	**0.98**	1.04	0.68	**0.86**
Prefoldin subunit 2	B1AQP2	PFDN2	0.67	1.15	**0.91**	0.83	0.99	**0.91**	1.12	1.07	**1.09**	0.98	0.77	**0.88**
Prostaglandin E synthase 3	A0A024RB32	PTGES3	0.73	1.07	**0.90**	1.02	1.10	**1.06**	1.51	1.45	**1.48**	1.45	1.13	**1.29**
Vacuolar protein sorting-associated protein 4B	A8K5D8	VPS4B	0.79	1.02	**0.90**	1.26	1.01	**1.14**	1.19	1.61	**1.40**	1.34	0.97	**1.16**
Adenosylhomocysteinase	P23526	AHCY	0.98	0.81	**0.90**	1.24	0.72	**0.98**	1.33	1.04	**1.19**	1.72	1.18	**1.45**
Oxysterol-binding protein 1	P22059	OSBP	0.87	0.91	**0.89**	1.03	0.94	**0.99**	0.79	1.07	**0.93**	0.83	0.81	**0.82**
N-alpha-acetyltransferase 50	C9J5D1	NAA50	0.83	0.95	**0.89**	0.98	0.93	**0.96**	1.21	1.34	**1.27**	1.27	0.95	**1.11**
Density-regulated protein	A0A024RBR3	DENR	0.84	0.92	**0.88**	1.41	0.95	**1.18**	1.23	1.27	**1.25**	1.39	0.87	**1.13**
Mannose-1-phosphate guanyltransferase alpha	A0A024R482	GMPPA	0.78	0.98	**0.88**	0.93	0.65	**0.79**	0.76	0.86	**0.81**	1.01	0.69	**0.85**
Inorganic pyrophosphatase	V9HWB5	PPA1	0.73	1.01	**0.87**	1.24	0.94	**1.09**	1.47	1.08	**1.28**	1.60	1.17	**1.39**
Cofilin-1	V9HWI5	CFL1	0.72	1.01	**0.87**	0.95	0.89	**0.92**	0.82	0.92	**0.87**	1.06	0.64	**0.85**
N-alpha-acetyltransferase 15, NatA auxiliary subunit	B2RBE5	NAA15	0.67	1.04	**0.85**	1.05	1.04	**1.04**	1.43	1.52	**1.48**	1.80	1.45	**1.63**
Echinoderm microtubule-associated protein-like 4	Q9HC35	EML4	0.72	0.99	**0.85**	0.90	0.84	**0.87**	1.21	1.02	**1.12**	0.83	1.58	**1.20**

***S3D Table**. Cont.*

Protein name	Uniprot	Symbol	6 hpi			12 hpi			24 hpi			36 hpi		
			Ad2(L)/ Mock(H)	Mock(L)/ Ad2(H)	Average	Ad2(L)/ Mock(H)	Mock(L)/ Ad2(H)	Average	Ad2(L)/ Mock(H)	Mock(L)/ Ad2(H)	Average	Ad2(L)/ Mock(H)	Mock(L)/ Ad2(H)	Average
Nascent polypeptide-associated complex subunit alpha	F8VZJ2	NACA	0.70	1.01	**0.85**	0.88	1.14	**1.01**	1.34	0.65	**0.99**	0.69	1.05	**0.87**
Superoxide dismutase [Cu-Zn]	V9HWC9	SOD1	0.70	0.99	**0.85**	1.28	0.62	**0.95**	1.11	1.24	**1.18**	1.16	0.81	**0.99**
Fatty acid synthase	P49327	FASN	0.90	0.80	**0.85**	0.70	0.74	**0.72**	0.79	1.11	**0.95**	1.07	1.04	**1.05**
Synembryn-A	Q9NPQ8	RIC8A	0.69	0.99	**0.84**	0.66	0.82	**0.74**	1.46	1.17	**1.31**	0.89	1.13	**1.01**
Cysteine and glycine-rich protein 1	B4DY28	CSRP1	0.64	1.04	**0.84**	0.83	1.00	**0.91**	0.74	1.16	**0.95**	0.89	0.67	**0.78**
C-1-tetrahydrofolate synthase, cytoplasmic	A0A024R652	MTHFD1	0.84	0.84	**0.84**	0.87	0.67	**0.77**	1.18	1.31	**1.25**	2.00	1.28	**1.64**
Protein TFG	Q7Z426	TFG	0.87	0.81	**0.84**	1.27	0.90	**1.09**	1.18	0.66	**0.92**	0.75	0.73	**0.74**
Ezrin	Q6NUR7	EZR	0.69	0.98	**0.84**	0.62	0.88	**0.75**	0.95	0.89	**0.92**	0.70	0.59	**0.65**
Cytosolic acyl coenzyme A thioester hydrolase	O00154	ACOT7	0.70	0.96	**0.83**	0.99	0.97	**0.98**	1.30	1.19	**1.24**	1.37	0.97	**1.17**
Ataxin-10	Q9UBB4	ATXN10	0.67	0.97	**0.82**	0.86	0.98	**0.92**	1.54	1.33	**1.43**	1.65	0.90	**1.27**
Glutaredoxin-3	A0A140VJK1	GLRX3	0.91	0.74	**0.82**	1.18	0.94	**1.06**	1.58	1.67	**1.63**	1.57	1.30	**1.43**
Mitogen-activated protein kinase	Q1HBJ4	MAPK1	0.72	0.91	**0.82**	1.05	0.85	**0.95**	1.09	1.13	**1.11**	0.86	0.66	**0.76**
Uroporphyrinogen decarboxylase	P06132	UROD	0.95	0.67	**0.81**	1.13	0.63	**0.88**	1.32	1.22	**1.27**	1.42	0.76	**1.09**
AH receptor-interacting protein	B7SBB1	AIP	0.65	0.97	**0.81**	0.95	0.65	**0.80**	1.30	1.16	**1.23**	1.35	1.14	**1.24**
Endophilin-A2	Q9UQD4	SH3GL1	0.65	0.96	**0.81**	0.84	0.87	**0.86**	0.63	0.84	**0.74**	0.86	0.71	**0.79**
Adenosine kinase	P55263	ADK	0.92	0.68	**0.80**	1.40	0.62	**1.01**	1.36	0.64	**1.00**	1.16	0.79	**0.98**
Exportin-2	P55060	CSE1L	0.82	0.77	**0.80**	1.05	0.70	**0.88**	1.16	1.07	**1.12**	1.14	0.65	**0.89**
ATP-dependent RNA helicase DDX19A	Q9NUU7	DDX19A	0.70	0.88	**0.79**	1.36	1.00	**1.18**	1.42	1.52	**1.47**	1.70	1.21	**1.45**
Cyclin-dependent kinase inhibitor 2A	J3QRG6	CDKN2A	0.78	0.77	**0.78**	1.14	0.93	**1.04**	1.14	1.45	**1.30**	1.48	1.20	**1.34**
Eukaryotic initiation factor 4A-I	A8K7F6	EIF4A1	0.60	0.94	**0.77**	0.93	1.01	**0.97**	0.94	1.31	**1.13**	1.12	1.26	**1.19**
Protein NOXP20	A0A024R9V7	FAM114A1	0.63	0.90	**0.77**	0.74	0.91	**0.82**	1.29	0.74	**1.01**	1.08	0.65	**0.87**
GTP-binding nuclear protein Ran	B5MDF5	RAN	0.64	0.90	**0.77**	1.09	0.91	**1.00**	1.09	1.06	**1.07**	1.02	0.88	**0.95**
Transcription factor BTF3	P20290	BTF3	0.66	0.87	**0.76**	0.73	1.25	**0.99**	1.68	1.56	**1.62**	1.92	1.68	**1.80**
Prefoldin subunit 5	Q99471	PFDN5	0.65	0.87	**0.76**	0.79	1.06	**0.92**	0.84	1.18	**1.01**	0.71	0.62	**0.67**
Ribosomal protein S6 kinase alpha-3	B4DKZ2	RPS6KA3	0.62	0.90	**0.76**	0.98	0.70	**0.84**	1.33	1.21	**1.27**	1.39	0.89	**1.14**
Protein transport protein Sec31A	D6REX3	SEC31A	0.68	0.84	**0.76**	0.83	0.73	**0.78**	0.74	0.89	**0.81**	1.04	0.71	**0.87**
Protein C10	Q99622	C12orf57	0.72	0.80	**0.76**	1.09	1.04	**1.06**	1.80	1.68	**1.74**	1.41	0.99	**1.20**
Putative heat shock protein HSP 90-beta 2	Q58FF8	HSP90AB2	0.60	0.90	**0.75**	0.70	0.76	**0.73**	0.84	1.22	**1.03**	1.04	1.10	**1.07**
NEDD8-conjugating enzyme Ubc12	A0A024R4T4	UBE2M	0.73	0.77	**0.75**	0.96	0.95	**0.96**	1.40	1.39	**1.39**	1.55	1.10	**1.33**
Eukaryotic peptide chain release factor subunit 1	P62495	ETF1	0.67	0.83	**0.75**	1.00	0.87	**0.94**	1.04	1.29	**1.17**	1.15	1.11	**1.13**
Programmed cell death protein 5	O14737	PDCD5	0.74	0.76	**0.75**	0.86	0.81	**0.84**	1.29	1.48	**1.38**	1.37	1.45	**1.41**
Nucleosome assembly protein 1-like 4	A0A024RCC9	NAP1L4	0.66	0.83	**0.74**	0.94	0.74	**0.84**	0.95	1.08	**1.02**	1.00	0.72	**0.86**
Heat shock protein HSP 90-beta	A0A024RD80	HSP90AB1	0.61	0.87	**0.74**	0.78	0.65	**0.72**	1.04	1.12	**1.08**	1.24	1.04	**1.14**
Small glutamine-rich tetratricopeptide repeat-containing protein alpha	O43765	SGTA	0.63	0.85	**0.74**	1.09	0.73	**0.91**	1.58	1.64	**1.61**	1.58	1.52	**1.55**
Nucleosome assembly protein 1-like 1	F8W020	NAP1L1	0.59	0.89	**0.74**	0.87	0.77	**0.82**	1.12	1.28	**1.20**	1.25	1.26	**1.25**
Calcyclin-binding protein	A0A024R904	CACYBP	0.68	0.79	**0.73**	0.84	0.74	**0.79**	1.21	1.22	**1.22**	1.11	1.02	**1.06**
Ubiquitin-like modifier-activating enzyme 1	A0A024R1A3	UBE1	0.72	0.73	**0.73**	0.93	0.64	**0.79**	0.98	0.85	**0.91**	1.08	0.61	**0.84**
Suppressor of G2 allele of SKP1 homolog	A8K5T7	SUGT1	0.67	0.78	**0.72**	0.99	0.76	**0.87**	1.30	1.42	**1.36**	1.31	1.27	**1.29**
Lipoma-preferred partner	B7ZLW0	LPP	0.66	0.76	**0.71**	0.92	0.80	**0.86**	1.30	1.00	**1.15**	1.71	0.82	**1.26**
Importin-5	A0A024RDY0	RANBP5	0.61	0.79	**0.70**	0.72	0.72	**0.72**	1.00	0.93	**0.96**	1.25	0.96	**1.10**
Importin-7	O95373	IPO7	0.59	0.79	**0.69**	0.77	0.69	**0.73**	0.94	1.18	**1.06**	0.99	1.10	**1.05**
Serine/threonine-protein phosphatase 2A 65 kDa regulatory subunit A	A8K7B7	PPP2R1A	0.60	0.72	**0.66**	0.79	0.65	**0.72**	0.94	0.98	**0.96**	0.74	0.62	**0.68**
Microtubule-associated protein RP/EB family member 1	B4DM33	MAPRE1	0.63	0.64	**0.64**	0.81	1.25	**1.03**	1.14	1.44	**1.29**	1.93	1.50	**1.71**
Platelet-derived growth factor receptor alpha	P16234	PDGFRA	-0.79	-0.81	**-0.80**	-1.14	-0.68	**-0.91**	-1.86	-1.22	**-1.54**	-1.65	-1.80	**-1.72**
Tissue factor	P13726	F3	-0.60	-1.08	**-0.84**	-1.35	-1.09	**-1.22**	-1.67	-2.12	**-1.90**	-2.52	-2.76	**-2.64**
Plasminogen activator inhibitor 1	A0A024QYT5	SERPINE1	-1.15	-0.60	**-0.87**	-1.86	-1.06	**-1.46**	-2.16	-1.80	**-1.98**	-3.52	-2.04	**-2.78**
Collagen alpha-2(I) chain	A0A087WTA8	COL1A2	-0.73	-1.14	**-0.93**	-1.36	-1.57	**-1.47**	-2.20	-1.66	**-1.93**	-2.34	-2.51	**-2.43**
Thrombospondin-1	P07996	THBS1	-1.10	-1.58	**-1.34**	-1.96	-2.36	**-2.16**	-1.64	-3.49	**-2.57**	-2.04	-2.61	**-2.32**
Collagen alpha-1(I) chain	P02452	COL1A1	-1.32	-2.29	**-1.81**	-1.72	-1.78	**-1.75**	-2.23	-1.92	**-2.08**	-3.31	-2.82	**-3.06**
