# Supplementary material for: Time-resolved proteomics of adenovirus infected cells
Source: PLoS One. 2018 Sep 25;13(9):e0204522. doi: 10.1371/journal.pone.0204522 (PMC6155545; doi:10.1371/journal.pone.0204522)
Supplement: S5 Table — (PDF) [file pone.0204522.s005.pdf]

S5 Table. Differentially altered phosphorylated sites found in IMR-90 cells post adenovirus type 2 infection (in log2 scale).

Position	Amino acid	Protein	Protein names	Gene name	6 hpi	12 hpi	24 hpi	36 hpi
82	S	V9HW43	Heat shock protein beta-1	HSPB1	1.02		1.83	
21	S	V9HWK1	Triosephosphate isomerase	TPI1	0.99	1.33	2.18	
141	S	Q13177	Serine/threonine-protein kinase PAK 2	PAK2		1.09		
2152	S	P21333	Filamin-A	FLNA		0.92	1.22	0.95
18	S	P16401	Histone H1.5	HIST1H1B		-1.96		
18	T	Q4VB24	Histone H1.4	HIST1H1E			1.01	
268	S	Q6DCA8	Bcl-2-associated transcription factor 1	BCLAF1			1.59	
39	S	V9HWE1	Vimentin	VIM			1.31	1.43
56	S	V9HWE1	Vimentin	VIM				-1.94
6	S	C9JKI3	Caveolin 1	CAV1			-0.95	-1.12
37	S	Q2TNI1	Caveolin 1	CAV1			-0.78	-0.95
112	S	P50479	PDZ and LIM domain protein 4	PDLIM4			-0.75	
44	S	F5H2U8	High mobility group protein HMGI-C	HMGA2				1.20
583	S	P27824	Calnexin	CANX				-0.85
390	S	P02545	Prelamin-A/C	LMNA				-2.55
392	S	P02545	Prelamin-A/C	LMNA			-0.89	
