# Supplementary figures and images for: Time-resolved proteomics of adenovirus infected cells
Source: PLoS One. 2018 Sep 25;13(9):e0204522. doi: 10.1371/journal.pone.0204522 (PMC6155545; doi:10.1371/journal.pone.0204522)

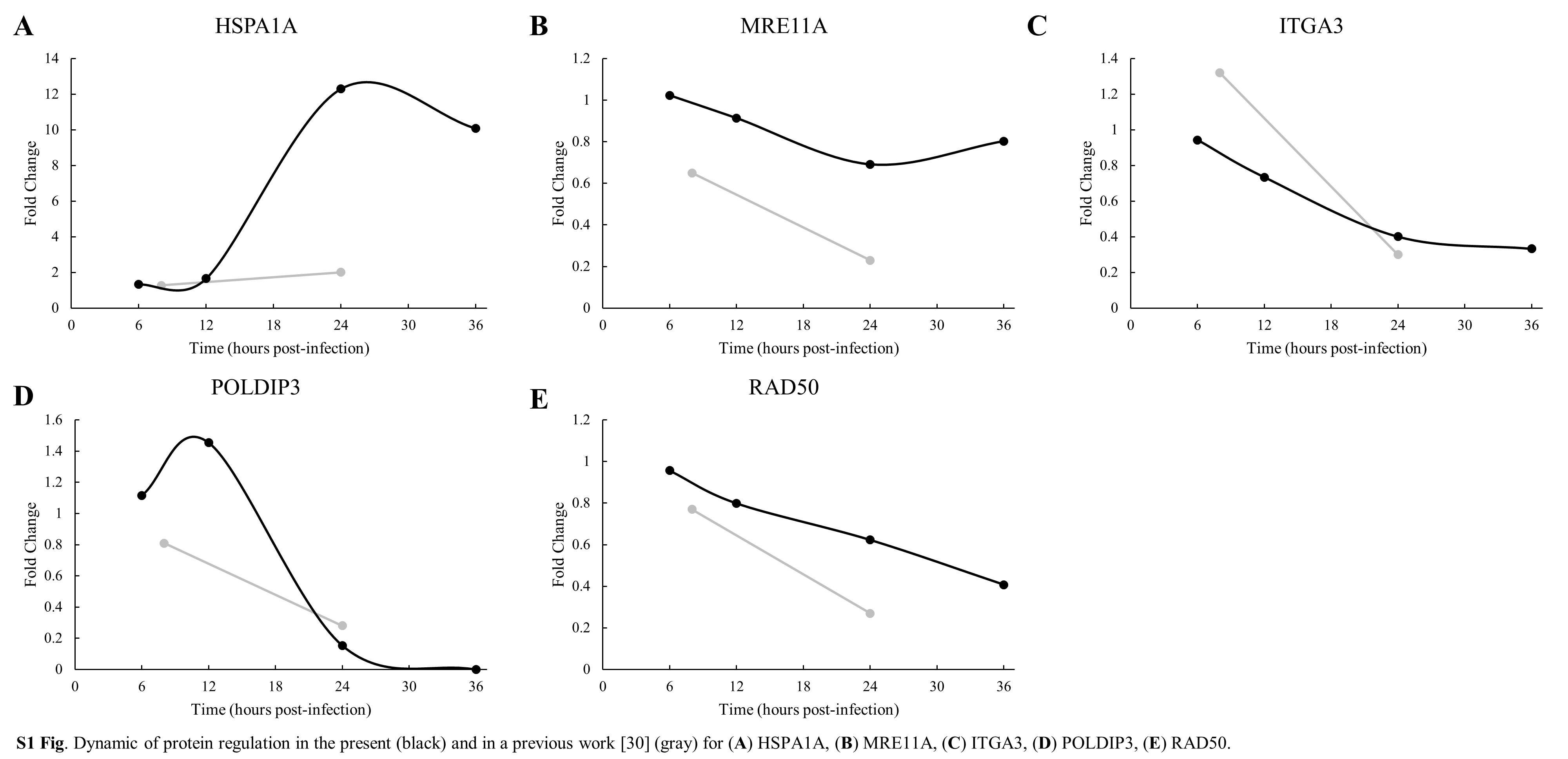

Supplement: S1 Fig — (TIF) [file pone.0204522.s006.TIF]

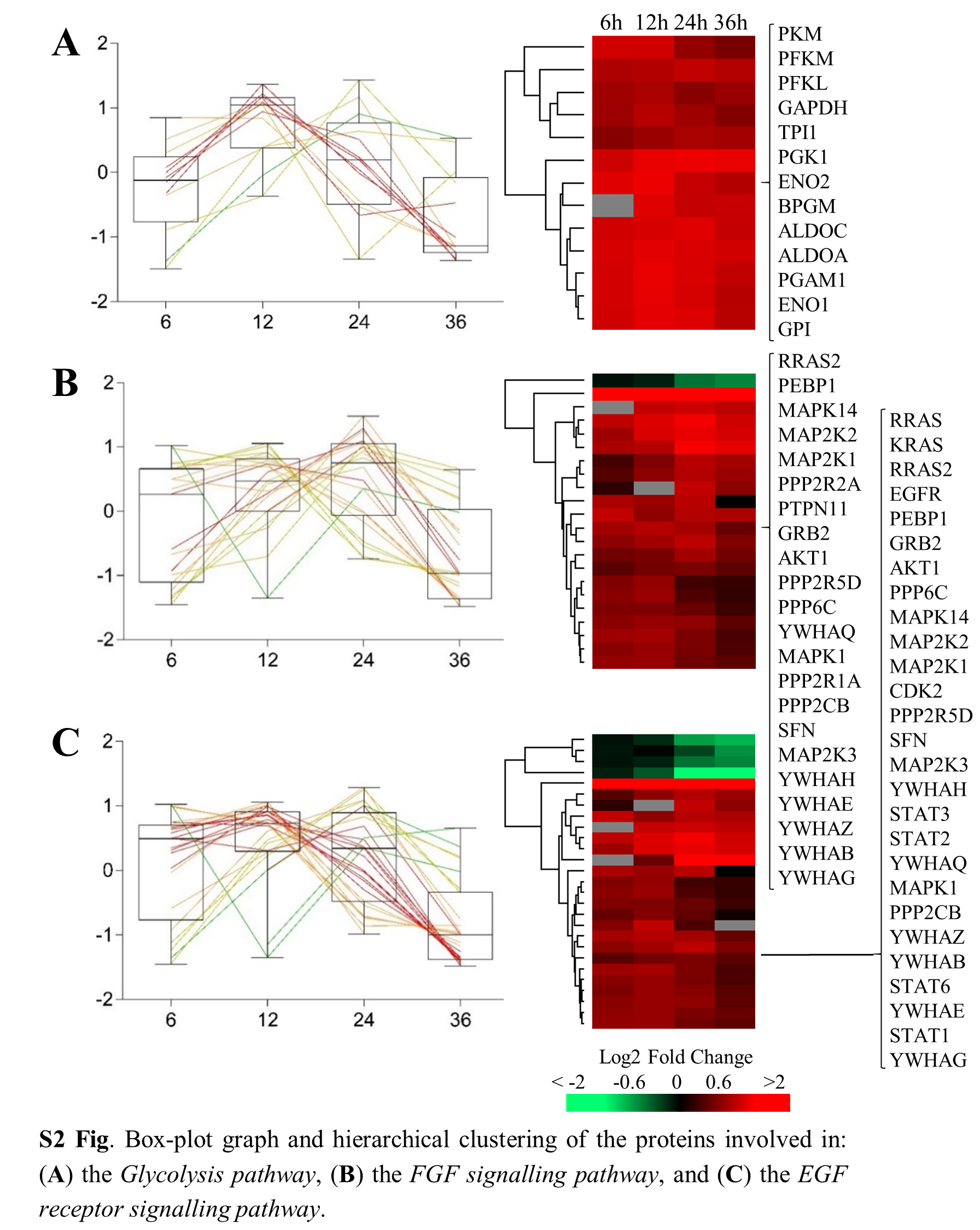

Supplement: S2 Fig — (TIF) [file pone.0204522.s007.TIF]

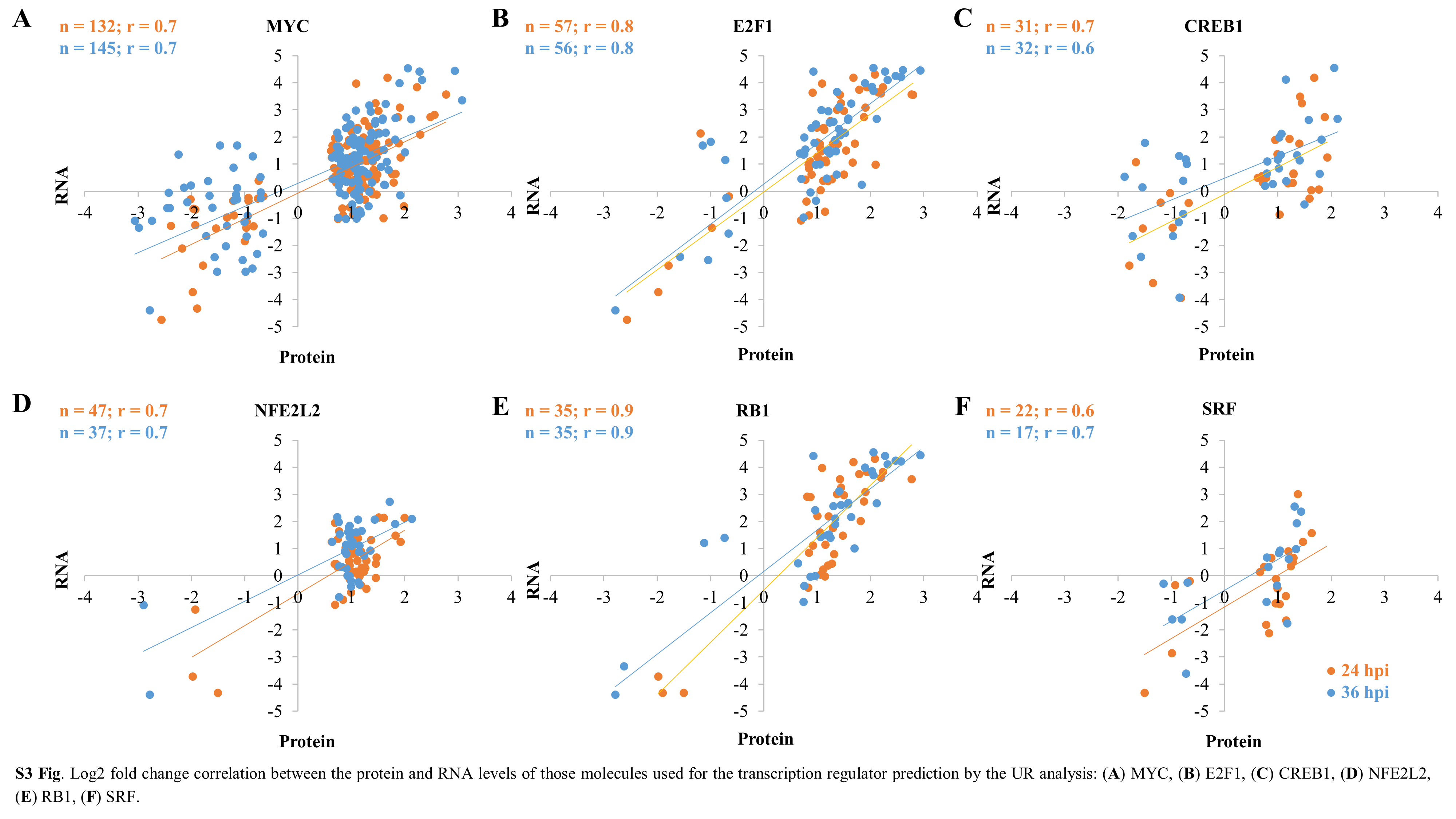

Supplement: S3 Fig — (TIF) [file pone.0204522.s008.TIF]
